# Rapid identification of causative insertions underlying *Medicago truncatula Tnt1* mutants defective in symbiotic nitrogen fixation from a forward genetic screen by whole genome sequencing

**DOI:** 10.1186/s12864-016-2452-5

**Published:** 2016-02-27

**Authors:** Vijaykumar Veerappan, Mehul Jani, Khem Kadel, Taylor Troiani, Ronny Gale, Tyler Mayes, Elena Shulaev, Jiangqi Wen, Kirankumar S. Mysore, Rajeev K. Azad, Rebecca Dickstein

**Affiliations:** Department of Biological Sciences, University of North Texas, 1155 Union Circle #305220, Denton, TX 76203 USA; Plant Biology Division, The Samuel Roberts Noble Foundation, Ardmore, OK 73401 USA; Department of Mathematics, University of North Texas, Denton, TX 76203 USA

**Keywords:** Whole genome sequencing, *Medicago truncatula*, Symbiotic nitrogen fixation, Nodulation, *Tnt1* mutants, Forward genetics, TAIL-PCR, *NIN*, *PLC-like*, *DNF2*

## Abstract

**Background:**

In the model legume *Medicago truncatula*, the near saturation genome-wide *Tnt1* insertion mutant population in ecotype R108 is a valuable tool in functional genomics studies. Forward genetic screens have identified many *Tnt1* mutants defective in nodule development and symbiotic nitrogen fixation (SNF). However, progress toward identifying the causative mutations of these symbiotic mutants has been slow because of the high copy number of *Tnt1* insertions in some mutant plants and inefficient recovery of flanking sequence tags (FSTs) by thermal asymmetric interlaced PCR (TAIL-PCR) and other techniques.

**Results:**

Two *Tnt1* symbiotic mutants, NF11217 and NF10547, with defects in nodulation and SNF were isolated during a forward genetic screen. Both TAIL-PCR and whole genome sequencing (WGS) approaches were used in attempts to find the relevant mutant genes in NF11217 and NF10547. Illumina paired-end WGS generated ~16 Gb of sequence data from a 500 bp insert library for each mutant, yielding ~40X genome coverage. Bioinformatics analysis of the sequence data identified 97 and 65 high confidence independent *Tnt1* insertion loci in NF11217 and NF10547, respectively. In comparison to TAIL-PCR, WGS recovered more *Tnt1* insertions. From the WGS data, we found *Tnt1* insertions in the exons of the previously described *PHOSPHOLIPASE C (PLC)-like* and *NODULE INCEPTION (NIN)* genes in NF11217 and NF10547 mutants, respectively. Co-segregation analyses confirmed that the symbiotic phenotypes of NF11217 and NF10547 are tightly linked to the *Tnt1* insertions in *PLC-like* and *NIN* genes, respectively.

**Conclusions:**

In this work, we demonstrate that WGS is an efficient approach for identification of causative genes underlying SNF defective phenotypes in *M. truncatula Tnt1* insertion mutants obtained via forward genetic screens.

**Electronic supplementary material:**

The online version of this article (doi:10.1186/s12864-016-2452-5) contains supplementary material, which is available to authorized users.

## Background

Symbiotic nitrogen fixation (SNF) in legumes takes place in nodules, specialized organs that initiate by differentiation of root cells during invasion of the root by soil bacteria collectively known as rhizobia. Ultimately, the rhizobia are deposited within host plant cells, separated by a plant-derived membrane. Within mature nodules, rhizobia convert atmospheric nitrogen to bioavailable forms which it exchanges for photoassimilates from the plant host. This mutually beneficial symbiosis provides legumes and subsequent crops with a renewable nitrogen source. Huge changes in gene expression in both the plant and rhizobia are observed during the differentiation to functional nodules. Genetics has uncovered many rhizobial genes required for SNF, but many plant genes essential to SNF have yet to be discovered [1]. Significant progress has been made via forward genetic studies in identifying essential plant genes required for the early Nod-factor signaling pathway [2]. This pathway initiates nodule-specific plant gene expression in response to rhizobial lipochitooligosaccharide molecules called nodulation (Nod) factors. Although some genes have been discovered that are required for steps after Nod-factor signaling, many still await discovery and characterization.

Forward genetics can identify new genes essential for SNF, not biased by our presuppositions. Successful forward genetics projects require an efficient mutagenesis technique to induce sufficient number of random mutations to saturate the genome and also fast and robust methods to identify the causative mutations in genes underlying mutant phenotypes.

*Medicago truncatula* (barrel medic) is an excellent model to study legume-rhizobia interactions during SNF because of its ease of laboratory manipulation and the availability of extensive genetic and genomic resources [3, 4]. In *M. truncatula*, a large collection of genome-wide insertion mutants has been developed using the tobacco *Tnt1* (transposable element of *Nicotiana tabacum*) retrotransposon [5]. There are 21,000 *Tnt1* insertion lines containing approximately 520,000 random insertions available as a community resource for functional genomics studies [6]. The *Tnt1* transposon is a 5.3 Kb long autonomous *copia*-like element first isolated from tobacco (*N. tabacum*) [7]. *Tnt1* sequences encode a capsid-related protein (GAG), a protease (PR), an integrase (INT), a reverse transcriptase (RT) and ribonuclease H (RH), and contain a 610 bp long-terminal repeat (LTR) flanking each end of *Tnt1* [8]. *Tnt1* transposes autonomously by a copy-and-paste mechanism through an RNA intermediate during somatic embryogenesis in tissue culture, thereby causing large numbers of random insertions across the genome [5, 8, 9]. Previous studies in *M. truncatula,* based on Southern blot analyses and flanking sequence tags (FSTs) isolated by TAIL-PCR, established an average of 25 insertions per *Tnt1* line, with individual lines containing 6 to 59 independent insertions [5]. *Tnt1* has also been successfully used in large-scale genome-wide insertional mutagenesis of several other heterologous plant species including lettuce [10], soybean [11] and potato [12].

High-copy numbers of *Tnt1* insertions in the *M. truncatula* mutant lines are advantageous because fewer lines need to be generated to saturate the genome and fewer plants need to be screened in forward genetic screens to find mutants defective in pathways of interest. Near-saturation mutagenesis also increases the success rates of reverse genetic screening to find *Tnt1* insertions in genes of interest [6]. However, high numbers of *Tnt1* insertions still pose significant challenges for forward genetic screens with recovery of FSTs a rate-limiting step. Traditional methods of FST identification, such as TAIL-PCR, adapter ligation PCR and plasmid rescue techniques, are not always efficient at identifying all the FSTs in individual *Tnt1* mutants.

In *M. truncatula*, numerous mutants that are defective in nodule development and symbiotic nitrogen fixation were identified by forward genetic screening of *Tnt1* insertion populations [13]. Despite the near-saturation mutagenesis of *Tnt1* insertion lines and the collection of mutants available for forward genetic screens, causative mutations for only a limited number of lines have been identified in this population by forward genetics [14, 15]. Whole genome sequencing (WGS) has revolutionized the identification of insertion mutations, caused by insertion of transposons or transfer-DNAs (T-DNAs), underlying the defective phenotypes of mutants from diverse organisms [16–19].

In this report, we demonstrate the successful use of WGS technology for the rapid identification of causative genes of *M. trunctaula Tnt1* mutants defective in nodulation identified from a forward genetic screen. We compare WGS results for *Tnt1* FSTs with those obtained by TAIL-PCR and find that the WGS approach is more efficient.

## Results and discussion

### Forward genetic screening for *M. truncatula* mutants with nodulation defects

**Fig. 1:**
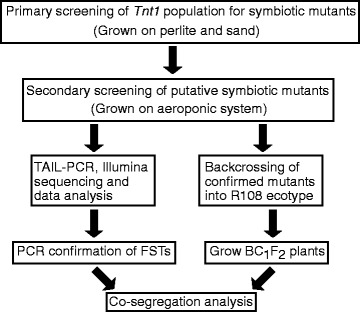
Overview of forward genetic screening for symbiotic mutants and identification of causative genes. Steps in forward genetic screening and identification of causative genes responsible for *M. truncatula Tnt1* mutants defective in nodule development and symbiotic nitrogen fixation are shown

To identify novel genes required for nodule development and SNF, we performed a forward genetic screen using the *Tnt1* insertion population in the *M. truncatula* R108 ecotype background [5]. Primary screening for mutants was conducted at the *M. truncatula* community mutant screening workshops at the S. R. Noble Foundation. Plants were grown on a mixture of perlite and sand (3:1) and regularly irrigated with media containing low nitrate (0.5 mM KNO_3_). Plants were inoculated with rhizobial strain *Sinorhizobium meliloti* Sm1021 [20] and screening was performed 4 weeks post inoculation (Fig. 1). When grown under low nitrate and symbiotic conditions, R108 wild-type (WT) plant shoots are green with roots having large ovoid pink nodules. The pink color of WT nodules is an indicator of efficient N_2_ fixation, caused by the abundant leghemoglobin protein [21]. In contrast, most SNF mutants show restricted shoot growth with anthocyanin accumulation in aerial parts and have small bumps (Nod+/-), spherical white nodules (Fix-) or pinkish white (Fix+/-) nodules on their roots instead of distinct pink nodules. Approximately twelve R_1_ plants per *Tnt1* line were screened for defective symbiotic phenotypes and categorized by the severity of the defects observed [13] to identify putative mutants.

Secondary screening was performed to confirm phenotypes of putative mutants, using an aeroponic system as described previously [22, 23]. The rhizobial strain *S. meliloti Rm41 hemA:lacZ* was used in the secondary screening because *Rm41* is more efficient in inducing nodulation and SNF in the R108 ecotype [24]. At 15 dpi, mutant plants were characterized for nitrogen deficiency phenotypes: leaf color and nodule shape, nodule color and rhizobial occupancy of sectioned nodules using X-Gal (5-Bromo-4-chloro-3-indolyl-P-galactopyranoside) staining for the *lacZ* gene contained in the rhizobial strain. Mutants with clear nodule defects and SNF phenotypes were selected for further characterization. Among the mutants chosen for further characterization were those from lines NF11217 and NF10547, both with Nod + Fix- phenotypes.

### Phenotypic characterization and segregation analysis of NF11217 and NF10547 mutants

**Fig. 2:**
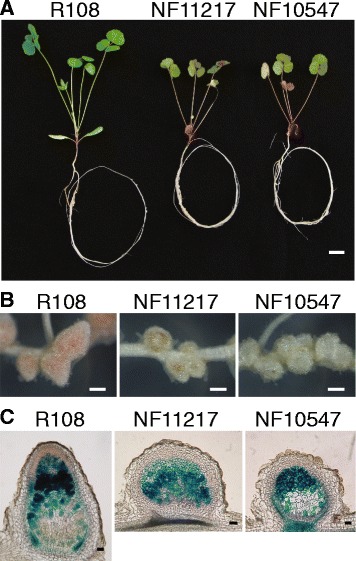
Phenotypic characterization of NF11217 and NF10547 plants. Plants were grown on aeroponic chambers in the presence of 5 mM ammonium nitrate for 5 days, followed by 5 days without nitrogen, and subsequently inoculated with *S. meliloti Rm41 hemA:lacZ*. Phenotyping was performed 15 days post inoculation. **a** Whole plants. WT leaves and petioles are green while the mutants’ leaves and petioles are reddish purple, caused by anthocyanin accumulation during nitrogen deficiency. Scale bar = 1 cm. **b** Visible nodule phenotype. WT nodules are pink; in comparison the mutants’ nodules are brownish or white. Scale bar = 0.5 mm. **c** Rhizobial occupancy nodule phenotype. The nodules were fixed and stained with X-Gal (5-bromo-4-chloro-3-indolyl-beta-D-galacto-pyranoside), sectioned to 100-micron-thickness, and imaged using bright field. Scale bar = 100 μm

Individual plants from NF11217 and NF10547 lines show reddish purple leaves and form small spherical, white ineffective Fix- nodules (Fig. 2a, b). X-Gal stained nodule sections show that NF11217 and NF10547 nodules failed to form distinct nodule zones and show reduced rhizobial occupancy (Fig. 2c). To study the inheritance and penetrance of Nod + Fix- phenotypes, we backcrossed the mutants into the parental R108 ecotype [23]. All the BC_1_F_1_ plants from successful crosses showed wild-type shoot and nodule characteristics. The BC_1_F_1_ plants were allowed to self-fertilize. Phenotyping of BC_1_F_2_ plants showed a 3:1 (wild-type: mutant) segregation ratio for phenotypes associated with SNF defects (Table 1). These results indicate that the defective symbiotic phenotypes in NF10547 and NF11217 mutants are governed by monogenic, recessive mutations.

**Table 1:** Segregation analyses of NF11217 and NF10547

Cross (Female × Male)	Generation	Total Plants	Phenotype		*χ*2 ^a^
			Wild-type (Pink nodule and green shoot)	Nod + Fix- mutant (White nodule and purple plant)	
NF11217 × R108	F_1_	8	8	0	-
	F_2_	328	258	70	2.756
NF10547 × R108	F_1_	9	9	0	-
	F_2_	335	248	87	0.168

^*^ *χ* ^2^ analysis was calculated based on the expected 3:1 (wild-type: mutant) ratio, *P* > 0.05

### Recovery of flanking sequence tags (FSTs) using TAIL-PCR

The *M. truncatula Tnt1* mutant database contains 333,482 high confidence flanking sequence tags (FSTs) and 406,299 low confidence FSTs (http://medicago-mutant.noble.org/mutant/database.php). These FSTs were recovered by TAIL-PCR from R_0_ hemizygote plants [5]. In an effort to identify the mutations responsible for the defective symbiotic phenotypes in the relevant mutants, we used the available FSTs to develop genetic markers to map and potentially identify the causative *Tnt1* insertions. To identify the genes with *Tnt1* inserts, we performed BLAST analysis of the FSTs using R108 BLAST web browser (http://www.medicagohapmap.org/tools/r108_blastform) which aligns the FST genomic sequences to the R108 draft genome as well as the reference A17 genome [25, 26]. Because we anticipated that potential causative *Tnt1* insertions might be in nodule-specific genes, we cross-referenced to the *M. truncatula* gene expression atlas (MtGEA) database (http://mtgea.noble.org/v3/; [27]) and the A17 genome browser (JBrowse Mtv4.0; http://jcvi.org/medicago/browsers.php) for nodule gene expression.

For line NF11217, we found a total of 31 high confidence FSTs in the *M. truncatula Tnt1* database (Additional file 1) that mapped to the A17 genome (Additional file 2: Table S1). In addition, we performed a second round of TAIL-PCR from a single NF11217 Nod + Fix- plant in the R_1_ generation and recovered a total of 39 FSTs (Additional file 1). Among the 39 FSTs, 30 were new and not recovered from R_0_ plants in the first round of TAIL-PCR. Among these 30 R_1_ FSTs, 27 mapped to the A17 and R108 genomes, while two mapped only to the R108 genome, and one matched neither (Additional file 2: Table S1 and Table 2). Among the recovered 61 FSTs in total, none of the annotated genes interrupted by *Tnt1* insertions were nodule-specific based on the MtGEA database and A17 genome browser. A third round of TAIL-PCR from two single BC_1_F_2_ NF11217 Nod + Fix- mutant plants recovered 18 FSTs, with 10 novel FSTs compared to the previous two rounds. As a whole, we isolated 71 unique FSTs by TAIL-PCR in three different generations including R_0_, R_1_ and BC_1_F_2_. Among them, 67 were mapped to unique sites in the A17 genome, 3 matched only to the R108 genome and there was one FST that did not match to either A17 or R108 genomes. None of the recovered FSTs were mapped to nodule-specific genes (Additional file 2: Table S1 and Table 2).

**Table 2:** Recovery of flanking sequence tags (FSTs) from *Tnt1* insertion mutants NF11217 and NF10547 using TAIL-PCR from different generations

*Tnt1* mutant line	Generation	Total FSTs	Novel FSTs^a^
NF11217	R_0_	31	31
	R_1_	39	30
	BC_1_F_2_	18	10
	Total	88	71
NF10547	R_0_	14	14
	R_2_	25	24
	Total	39	38

^a^Insertion locations of FSTs in the A17 genome from R_0_ versus R_1_, and R_0_ and R_1_ versus BC_1_F_2_ and R_0_ versus R_1_ were compared for NF11217 and NF10547, respectively to identify novel, non-redundant FSTs

For the NF10547 mutant, we obtained 14 FSTs (R_0_ generation) from *Tnt1* mutant database (Additional file 3) and among them 10 FSTs were mapped to the A17 genome. None of these FSTs were found to be obvious candidate genes based on nodule specific gene expression (Additional file 4: Table S2). We performed an additional TAIL-PCR from an individual R_2_ mutant and recovered 24 novel FSTs. As noted below, the same individual R_2_ mutant was subjected to WGS analysis. The TAIL-PCR analysis yielded a total of 38 non-redundant FSTs from NF10547 TAIL-PCR. Among them, only 34 FSTs were mapped to the A17 genome (Additional file 3; Additional file 4: Table S2 and Table 2).

### Whole genome sequencing to recover *Tnt1* insertion sites

WGS approaches have been shown to be more sensitive and efficient, and have identified the causative T-DNA insertions in *Arabidopsis thaliana* and fungal mutants obtained from forward genetic screens in which TAIL-PCR, plasmid rescue and adapter ligation PCR techniques failed [16, 17, 19]. Hence, we attempted WGS approach on the *M. truncatula* mutants using the Illumina Hiseq 2000 platform that provided 188 and 180 million 90 bp paired-end (PE) clean reads from 500 bp insert libraries representing 44X and 40X total genome coverages of NF11217 and NF10547 mutants, respectively (Table 3).

**Table 3:** Summary of whole genome sequencing data and coverage estimates

	*Tnt1* mutant line	
	NF11217	NF10547
Total number of raw reads (millions)	196.31	184.11
Total nucleotides of raw reads (Giga bases)	17.67	16.57
Total number of clean reads (millions)^a^	188.03	176.77
Total nucleotides of clean reads (Giga bases)	16.92	15.91
Total genome coverage of clean reads^b^	44X	41X

^a^After removing the adapter sequences and reads that contain >50 % low quality bases

^b^Total genome coverage was calculated based on the estimated *Medicago truncatula* A17 genome size of 384 Mb Krishnakumar et al., [26] using the formula clean bases/reference genome size

**Fig. 3:**
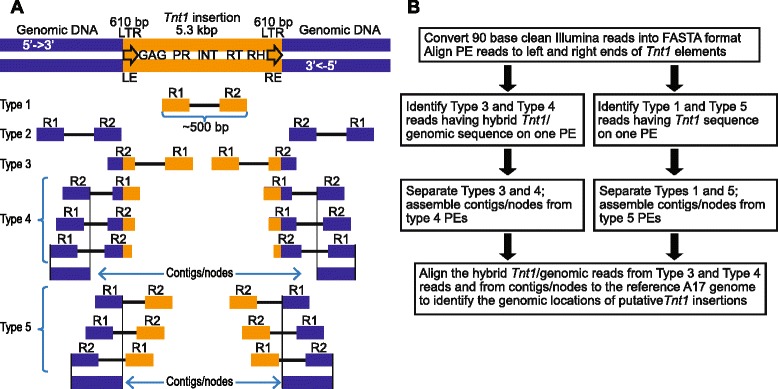
Strategy for bioinformatic analyses of whole genome sequencing (WGS) data to identify *Tnt1* insertion sites. **a** Tobacco (*Nicotiana tabacum*) type I retrotransposon (*Tnt1*) genomic structure. *Tnt1* transposon sequences contain 610 bp long terminal repeats (LTRs) on both left (LE) and right ends (RE), and encode a capsid protein (GAG), protease (PR), an integrase (INT), reverse transcriptase (RT) and RNAseH (RH). Paired-end (PE) sequencing reads obtained from *Tnt1* insertion mutants are classified into 5 different types: *Tnt1*-*Tnt1 (*type 1), genomic-genomic (type 2), hybrid-*Tnt1* or *Tnt1*-hybrid (type 3), genomic-hybrid or hybrid-genomic (type 4) and genomic-*Tnt1*or *Tnt1*-genomic (type 5) based on the sequence composition. Read types 3, 4, and 5 were used in our analyses. **b** Workflow for the identification of *Tnt1* insertion loci using the WGS data. To identify the *Tnt1* insertion loci, a local BLAST program [29] was used. Clean PE reads were aligned to 90 bp LE and RE *Tnt1* sequences to identify type 3 reads. These are putative “hybrids” containing *Tnt1*-genomic sequence junctions. Type 4 and 5 reads were used to develop contigs/nodes using the VELVET program [47] to assemble genomic fragments. Both hybrid reads and contigs/nodes were aligned to A17 reference genome by BLAST to obtain genomic co-ordinates of putative *Tnt1* insertion loci

Paired end (PE) sequencing generated two 90 bp short reads from both ends of a DNA fragment hereafter referred to as read 1 (R1) and read 2 (R2) (Fig. 3a). Each read from PE sequencing could match completely to the reference A17 genome (genomic), or *Tnt1* sequences only (*Tnt1*) or it could be a hybrid sequence comprised of parts of *Tnt1* element and R108 genome, thus representing the insertion site (hybrid). The PE reads (R1-R2) from WGS can be classified as follows based on the sequence composition: (i) Type 1: *Tnt1*-*Tnt1,* (ii) Type 2: genomic-genomic, (iii) Type 3: hybrid-*Tnt1* or *Tnt1*-hybrid, (iv) Type 4: genomic-hybrid or hybrid-genomic and (v) Type 5: genomic-*Tnt1* or *Tnt1*-genomic (Fig. 3a). Type 2 reads could be used to estimate the zygosity of insertion loci based on the genomic reads that mapped to the insertion junction sites [28]. The PE reads from Types 3, 4, and 5 are informative for the identification of the *Tnt1* insertion sites.

An overview of the data analysis of WGS data to identify the *Tnt1* insertion sites are outlined in Fig. 3b. To detect the *Tnt1* insertion sites from the WGS data, we employed a stand-alone BLAST program [29] set up on a Linux platform to align reads against the *Tnt1* sequence. This was done for all our BLAST analyses because the *M. truncatula* reference genome is from the A17 ecotype, while the *Tnt1* mutants are in R108 background, and therefore, alignment against *Tnt1* sequences alone circumvents the ambiguities because of polymorphisms between A17 and R108. We aligned the PE reads R1 and R2 to both the left and right ends of *Tnt1* and recovered Types 3, 4 and 5 reads (Fig. 3a). Type 4 and type 5 reads were assembled into contigs, called nodes. Subsequently, we aligned the hybrid reads and nodes against the A17 reference genome to identify the genomic co-ordinates of the *Tnt1* insertions. Ideally, each unique *Tnt1* insertion should be represented by all 6 types of supporting hybrid reads and nodes: Types 3, 4 and 5 for each side of the *Tnt1* insertion. Insertion loci with at least 3 supporting hybrid reads and/or nodes are considered high confidence (HC) whereas the rest of the loci are considered low confidence (LC). From this analysis, we identified 97 HC insertion loci for NF11217 with 89 mapped to the A17 reference genome and the remaining 8 mapped to the A17 scaffolds only (Additional file 5: Table S3 and Table 4). We also found 1078 LC insertion loci for NF11217 (Additional file 6: Table S4). For NF10547, we identified a total of 65 HC insertion loci. Among the HC loci, 52 were mapped to specific chromosomal locations while the remaining loci mapped to A17 scaffolds (Additional file 7: Table S5 and Table 4). For NF10547, 268 LC *Tnt1* loci were obtained (Additional file 8: Table S6). Mapping of some of these insertion loci showed that they are in the A17 scaffolds instead of chromosomal locations indicating gaps in the *M. truncatula* genome. The significance of the numerous LC insertion loci obtained for both lines is unclear.

**Table 4:** High confidence *Tnt1* insertion site statistics from whole genome sequencing

*Tnt1* mutant line	Total *Tnt1* insertion loci	*Tnt1* insertion loci	
		Mapped to A17 chromosomes	Mapped to A17 scaffolds
NF11217	97	89	8
NF10547	65	52	13

**Fig. 4:**
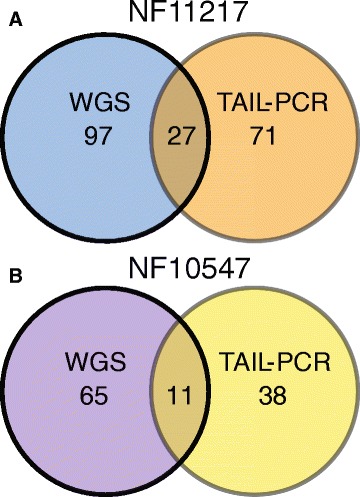
Comparison of *Tnt1* insertions obtained from TAIL-PCR and WGS. **a** Unique high confidence *Tnt1* insertion sites that were recovered from R_0_, R_1_ and BC_1_F_2_ generations of NF11217 by TAIL-PCR were compared to those obtained from a single BC_1_F_2_ plant by WGS. **b** Unique high confidence *Tnt1* insertion sites that were obtained from R_0_ and R_2_ generations of NF10547 were compared to those obtained by WGS from a single R_2_ plant

We attempted comparison between the HC *Tnt1* insertion locations obtained via TAIL-PCR and those obtained by WGS, although they are not strictly comparable because the TAIL-PCR *Tnt1* insertion locations were obtained from different generations of segregating and back-crossed populations, while the WGS data was obtained from only an individual BC_1_F_2_ (in the case of NF11217) or R_2_ (for NF10547) mutant plant. For NF11217, TAIL-PCR identified a total of 71 *Tnt1* insertions while WGS recovered 97 HC insertion loci, with 27 *Tnt1* insertion loci identified by both approaches (Additional file 9: Table S7 and Fig. 4a). For NF10547, 38 unique TAIL-PCR and 65 HC WGS *Tnt1* FSTs were found with 11 FSTs common to both datasets (Additional file 10: Table S8 and Fig. 4b). These data indicate that the WGS approach identified substantially more *Tnt1* insertion locations than TAIL-PCR. Additionally, the *Tnt1* insertion locations identified by WGS were from an individual BC_1_F_2_ or R_2_ mutant plant for NF11217 and NF10547 respectively, which have already lost some non-co-segregating *Tnt1* insertions present in the R_0_ and R_1_ generations that were subjected to TAIL-PCR.

### NF11217 and NF10547 mutants are novel *Tnt1* insertion alleles of *PLC-like* and *NIN* genes, respectively

**Fig. 5:**
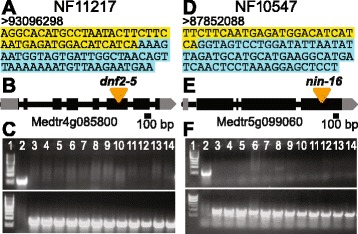
NF11217 and NF10547 contain novel *Tnt1* insertions in *NODULE INCEPTION* (*NIN*) and *PHOSPHOLIPASE C (PLC)-like*, respectively. **a** A hybrid read example obtained for NF11217 with the causative *Tnt1* insertion showing the *Tnt1* border sequence (*yellow*) and *PLC-like* sequence (*blue*). **b** Genomic structure of *PLC-like* indicating the *Tnt1* insertion site in NF11217. **c** Representative co-segregation data for *Tnt1* insertions in NF11217 co-segregating with Nod + Fix- plants. The top panel shows PCR products from PCR reactions with two primers specific for *PLC-like* genomic sequences, Medtr4g085800-1 F and Medtr4g085800-1R, of 585 bp; the lower panel shows PCR products with one *PLC-like* genomic primer, Medtr4g085800-1 F, and one *Tnt1* primer, Tnt-F1, of 805 bp. Lane 1 shows DNA markers (New England Biolabs, Beverly, MA; #N3232); lane 2, WT R108 DNA; and lanes 3-14, Nod + Fix- plants in the NF11217 BC_1_F_2_ population. **d** A hybrid read example obtained for NF10547 with the causative *Tnt1* insertion showing the *Tnt1* border sequence (yellow) and *NIN* sequence (*blue*). **e** Genomic structure of *NIN* indicating the *Tnt1* insertion site in NF10547. **f** Representative co-segregation data for *Tnt1* insertions in NF10547 co-segregating with Nod + Fix- plants. The top panel shows PCR products from PCR reactions with two primers specific for *NIN* genomic sequences, Medtr5g099060-1 F and Medtr5g099060-1R, of 503 bp; the lower panel shows PCR products with one *NIN* genomic primer, Medtr5g099060-1 F, and one *Tnt1* primer, Tnt-R1, of 810 bp. Lane 1 shows DNA markers; lane 2, WT R108 DNA; and lanes 3–14, Nod + Fix- plants in the NF10547 BC_1_F_2_ population. Additional PCR genotyping data for NF11217 and NF10547 WT-like and Nod + Fix- plants from BC_1_F_2_ can be found in Additional file 12: Table S9 and Additional file 13: Table S10, respectively. Genotyping primers are listed in Additional file 15: Table S11

To identify the causative *Tnt1* insertions underlying the nodulation defects in NF11217 and NF10547, we analyzed the genome coordinates of insertion loci from WGS data. One of the *Tnt1* insertion loci, Insertion-38, in NF11217 with 69 supporting reads/nodes (Additional file 5: Table S3; Additional file 9: Table S7) mapped to the previously characterized *DEFECTIVE IN NITROGEN FIXATION 2* (*DNF2*) gene encoding a PLC-like family protein (Medtr4g085800). BLAST analysis revealed this *Tnt1* insertion maps to the 6^th^ exon of *DNF2/PLC-like* (Fig. 5a). The PLC-like protein was reported to play an essential role in nodule development and symbiotic nitrogen fixation under non-permissive conditions [15, 30–32]. Mutant NF11217 plants have similar phenotypes (Fig. 2) to those of the previously characterized *dnf2* mutant NF0217 in our growth conditions (Additional file 11: Figure S1). Some *dnf2* mutant nodules have an apparent defense-like reaction producing brown-colored phenolic compounds, while others are white (Fig. 2b, c; Additional file 11: Figure S1), like the phenotypes described when plants are grown on media solidified with agar [15]. The reasons for the variation in nodule phenotype in *dnf2* mutant nodules is unknown [15, 32]. Genetic markers for the defective Medtr4g085800 allele were made and found to co-segregate with the defective SNF phenotype in the BC_1_F_2_ population, with all WT BC_1_F_2_ plants in the population carrying at least one WT allele of Medtr4g085800 (Fig. 5a; Additional file 12: Table S9). Hence, NF11217 is a new allele of *dnf2*, which we call *dnf2-5*.

Analysis of WGS data for NF10547 identified a *Tnt1* insertion in the fourth exon of a previously characterized nodule specific gene *NODULE INCEPTION* (*NIN*; Medtr5g099060). This insertion locus, Insertion-31, has 54 supporting reads/nodes (Additional file 7: Table S5; Additional file 10: Table S8) confirming the *Tnt1* insertion in *NIN* gene. *NIN* is a key transcriptional factor which plays a major role in the formation of infection threads, induction of cortical cell divisions and regulation of nodule formation [33–38]. In this case, the second round of TAIL-PCR obtained from a single NF10547 Nod + Fix- plant from R_2_ generation also identified a *Tnt1* insertion in *NIN* (Additional file 4: Table S2). To test whether the interrupted *NIN* gene is responsible for the underlying Nod + Fix- phenotype of NF10547 *Tnt1* mutant line, we performed co-segregation analysis using the BC_1_F_2_ population obtained from a NF10547 × R108 cross. PCR genotyping showed that all tested BC_1_F_2_ plants with a Nod + Fix- phenotype were homozygous for the *Tnt1* insertion in *NIN* gene whereas all the tested WT phenotype plants had at least one WT uninterrupted *NIN* allele (Fig. 5b and Additional file 13: Table S10). These data indicate that NF10547 contains a new *nin* allele, which we have named *nin-16.* Most of the previously reported *M. truncatula nin* mutants showed a Nod- phenotype [13, 34]. Conceptual translation of *nin-16* shows that it encodes a putative 795 amino acid hybrid protein, with the first 785 amino acid residues of NIN followed by ten amino acid residues encoded by *Tnt1* (Additional file 14: Figure S2). It has the first six conserved blocks of sequence found in NIN and NIN-like proteins, including the characteristic RWP-RKP domain thought to be important for dimerization and DNA binding activity in regulation of genes controlled by nitrogen status [33, 39], but lacks the last conserved sequence block containing the PB1 domain, putatively vital for hetero-dimerization [39, 40] (Additional file 14: Figure S2). Our genetic data lead us to hypothesize that the Fix- nodule phenotype in NF10547 is likely caused by the weak *nin-16* allele as opposed to a cryptic defect in a different, closely-linked gene. Other observations support the idea that a weak *nin* allele could have such a phenotype. First, there is a brief description of another *M. truncatula nin* allele, also a *Tnt1* insertion in the last exon of *NIN*, as Nod+/Fix-, while other *nin* alleles with *Tnt1* insertions in the last NIN exon are Nod- [13]. Second, in mature determinate *L. japonicus* nodules, *NIN* expression persists which indicates that NIN likely has a role later in nodule development as well as in nodule inception [33]. Third, *NIN* expression in indeterminate *M. truncatula* nodules has highest expression in the nodule infection zone and significant expression in the inter-zone and nitrogen fixing zones [41]. Our data indicate that NF10547 is expected to contain a partially functional NIN protein. This new allele could be valuable in further dissecting NIN’s function after nodule inception, in rhizobial infection and in nodule organogenesis.

Overall, our data show that while TAIL-PCR is useful in identifying the causative *Tnt1* FSTs in some mutants, WGS is more efficient in pinpointing the FSTs. In this work, with >40X total genome coverage, we were able to identify the mutations underlying the defects in two mutants. Previously, it was reported that 10X genome coverage is enough to identify 96 % of the insertions from *M. truncatula* [28]. The optimal sequencing coverage required to identify the causative insertions in mutants from a forward genetic screen was not reported previously for *M. truncatula*. WGS is increasingly becoming more cost-effective and accurate. The availability of several commercial vendors for genome sequencing and bioinformatics analysis has facilitated rapid data acquisition, allowing researchers to focus on the genetic characterization of identified genes and biochemical characterization of their encoded proteins, speeding up forward genetics. TAIL-PCR is still a valuable tool for forward genetics, but the success rate of WGS for forward genetic screens is significantly higher, as reported in previous studies in other organisms [16, 17, 19], and now established for *M. truncatula* by our study.

## Conclusions

In this work, we demonstrate that WGS is an efficient approach for the recovery of high numbers of *Tnt1* insertion sites from *M. truncatula Tnt1* insertion mutants. Our results demonstrated that WGS efficiency clearly surpassed that of TAIL-PCR. We also showed the utility of the WGS method in identifying relevant disrupted genes in two mutants isolated in a forward genetic screen as defective in SNF nodule development. This work uncovered a new *dnf2-5* allele with phenotypes similar to *dnf2* mutants previously described. It also described a new *nin-16* mutant that showed defects later in nodule development than other *nin* mutants. This weak *nin* allele is likely to be valuable in characterizing NIN’s functions later in nodule development, after nodule inception.

## Methods

### Plant materials, growth conditions and genetic crossing

*M. truncatula* plant growth conditions and genetic crossing procedures were as described [23].

### Nodule phenotyping, fixing of nodules, sectioning and X-Gal staining

Primary mutant screening was performed using *Tnt1* mutant population grown under low nitrate conditions (0.5 mM KNO_3_) on a mixture of perlite and sand (3:1) inoculated with a rhizobial strain *S. meliloti* Sm1021 as described in Yarce et al. [20]. Four weeks after inoculation, plants were uprooted and screened for visible defective symbiotic phenotypes. For secondary screening, putative mutants were grown on aeroponic chambers containing plant growth media [42] supplemented with 5 mM NH_4_NO_3_ for 5 days followed by 5 days of growth without any nitrogen source. Subsequently, plants were inoculated with *S. meliloti Rm41* carrying the *hemA:lacZ* reporter (gift from Dr. Pascal Ratet). Nodules were fixed and stained with X-Gal as described previously [43]. Nodule sections, 100 μm thick were obtained using a 1000 Plus model Vibratome (Vibratome, St. Louis, MO). Sections were observed and documented under an Olympus BX50 microscope using bright field settings.

### Preparation of genomic DNA and PCR genotyping

Genomic DNA (gDNA) from *M. truncatula* was prepared from mature leaves. gDNA for PCR genotyping and TAIL-PCR was prepared using an established method [44] and gDNA for whole genome sequencing was prepared using a modified CTAB method as described (www.monsanto.com/products/documents/dna-detection/dna_im.pdf). Genotyping primers used for co-segregation analysis were designed using R108 and A17 genomic sequences downloaded from the R018 BLAST browser from *M. truncatula* Hapmap website (http://www.medicagohapmap.org/tools/r108_blastform). PCR was performed in 20 μl reactions using Go-Taq Green Master Mix (Promega, Madison, WI; Cat. No. M7123). Primers sequences for genotyping and co-segregation analysis are listed in Additional file 15: Table S11.

### TAIL-PCR

Thermal asymmetric interlaced (TAIL)-PCR was performed as described [5, 45, 46]. For the primary PCR amplification, *Tnt1*-specific primers *Tnt1-F* (forward) or *Tnt1-R* (reverse) in combination with five different arbitrary degenerate (AD) primers AD1, AD2, AD3, AD5 and AD6 were used for each *Tnt1* individual mutant genomic DNA template. The 50-fold-diluted primary PCR products were used as templates for the secondary PCR. For the 2^nd^ PCR amplification, a nested *Tnt1*-specific primer which is close to the end of the *Tnt1* (*Tnt1-F1* or *Tnt1-R1*) was used for each individual template in combination with the same five AD primers that were used in the primary PCR amplification. After the 2^nd^ PCR amplification, PCR products were purified by Qiagen PCR Purification Kit (Qiagen, Valencia, CA; Cat. No. 28104), quantified by NanoDrop Spectrophotometer (Thermo Scientific, Wilmington, DE) and ligated to pGEM-T-Easy vector system (Promega, Madison, WI; Cat. No. A1360). Plasmids from 96 random white colonies were sequenced using Sanger sequencing from each side of the *Tnt1* for each mutant lines. Primers used in TAIL-PCR are listed in Additional file 15: Table S11.

### Library preparation and whole genome sequencing

Library preparation, whole genome sequencing and data analysis to obtain clean reads were performed by the Beijing Genomics Institute (BGI), China (http://bgi-international.com/us/?id=). Ninety bp PE reads were obtained from 500 bp insert libraries by sequencing on an Illumina HiSeq 2000 platform.

### Bioinformatics analysis

Alignments of PE reads to *Tnt1* sequences and the reference genome A17 were performed using BLAST programs [29] installed on a Linux machine. The BLAST output was parsed using a custom Perl script to identify the hybrid reads containing *Tnt1*-genomic junction sequences, as outlined in Fig. 3. Genomic reads obtained from PE sequences in which the other reads are either *Tnt1* or hybrid, were further developed into contigs/nodes using the VELVET program [47]. Hybrid reads and genomic contigs were aligned to the *M. truncatula* reference genome A17 using BLAST to identify the genome co-ordinates of putative *Tnt1* insertion sites. A Perl script was developed to cluster the hybrid reads and the VELVET-derived contigs from both the hybrid read paired-ends and the *Tnt1* paired-ends. The grouping of the reads was based on alignment to the A17 genome using BLAST. Each group or cluster (putative insertion) thus obtained was assigned a confidence level of either high or low, based on the number of supporting reads/nodes. Clusters derived from 3 or more reads/nodes were considered to be high confidence, otherwise they were annotated as low confidence. Clusters were sorted by their confidence level, then by chromosome number and position on the chromosome, and finally given unique insertion numbers based on this ordering.

### Availability of supporting data

WGS data that was used in identifying the *Tnt1* insertion sites were deposited in the NCBI Sequence Read Archive (SRA) (http://www.ncbi.nlm.nih.gov/sra) under the BioProject accession number PRJNA298564 with experiment accession numbers SRR2650316 (NF11217) and SRX1335995 (NF10547).

## Additional files

Additional file 1: List of sequences of all NF11217 flanking sequence tags (FSTs) obtained from R_0_, R_1_ and BC_1_ F_2_ generations by TAIL-PCR. (PDF 62 kb) Additional file 2: Table S1. Chromosomal locations of NF11217 TAIL-PCR FSTs recovered from R_0_, R_1_ and BC_1_F_2_ generations. (XLS 53 kb) Additional file 3: List of sequences of all NF10547 flanking sequence tags (FSTs) obtained from R_0_ and R_2_ generations by TAIL-PCR. (PDF 42 kb) Additional file 4: Table S2. Chromosomal locations of NF10547 TAIL-PCR FSTs recovered from R_0_ and R_2_ generations. (XLS 43 kb) Additional file 5: Table S3. List of high confidence *Tnt1* insertion loci and its supporting reads/nodes identified from NF11217 by WGS. (XLS 509 kb) Additional file 6: Table S4. List of low confidence *Tnt1* insertion loci and its supporting reads/nodes identified from NF11217 by WGS. (XLS 345 kb) Additional file 7: Table S5. List of high confidence *Tnt1* insertion loci and its supporting reads/nodes identified from NF10547 by WGS. (XLS 294 kb) Additional file 8: Table S6. List of low confidence *Tnt1* insertion loci and its supporting reads/nodes identified from NF10547 by WGS. (XLS 141 kb) Additional file 9: Table S7. Comparison of NF11217 high confidence *Tnt1* insertion loci identified from WGS vs TAIL-PCR. (XLS 48 kb) Additional file 10: Table S8. Comparison of NF10547 high confidence *Tnt1* insertion loci identified from WGS vs TAIL-PCR. (XLS 33 kb) Additional file 11: Figure S1. Comparison between nodule phenotypes of NF11217 (*dnf2-5*), NF0217 (*dnf2-2*) and R108 (WT). (PDF 673 kb) Additional file 12: Table S9. Co-segregation analysis of NF11217 Nod + Fix- phenotype with *Tnt1* insertion in *PLC-like* gene. (PDF 41 kb) Additional file 13: Table S10. Co-segregation analysis of NF10547 Nod + Fix- phenotype with *Tnt1* insertion in *NIN* gene. (PDF 42 kb) Additional file 14: Figure S2. Comparison of *Mtnin-16*′s encoded sequence with that of *Lotus japonicus NIN* and *M. truncatula NIN*. (PDF 797 kb) Additional file 15: Table S11. Sequences of primers used in this study. (PDF 7 kb)

## Abbreviations

AD: Arbitrary degenerate
BC: Back cross
BLAST: Basic local alignment search tool
DNA: Deoxyribonucleic acid
EMS: Ethyl methane sulfonate
FNB: Fast neutron bombardment
gDNA: Genomic DNA
HC: High confidence
INT: Integrase
LacZ: β-galactosidase
LC: Low confidence
LTR: Long-terminal repeat
*M. truncatula*: *Medicago truncatula*
MtGEA: *Medicago truncatula* gene expression atlas
*NIN*: *NODULE INCEPTION*
Nod: Nodulation
PCR: Polymerase chain reaction
PE: Paired-end
*PLC*: *PHOSPHOLIPASE C*
PR: Protease
R_0_: Regeneration_0_
R1: Read 1
RNA: Ribonucleic acid
RNase H: Ribonuclease H
RT: Reverse transcriptase
SNF: Symbiotic nitrogen fixation
TAIL-PCR: Thermal asymmetric interlaced (TAIL)-PCR
T-DNA: Transfer-DNA
*Tnt1*: Transposable element of *Nicotiana tabacum*
WGS: Whole genome sequencing
WT: Wild-type
X-Gal: 5-Bromo-4-chloro-3-indolyl-P-galactopyranoside
