# Supplementary material for: Rapid identification of causative insertions underlying Medicago truncatula Tnt1 mutants defective in symbiotic nitrogen fixation from a forward genetic screen by whole genome sequencing
Source: BMC Genomics. 2016 Feb 27;17:141. doi: 10.1186/s12864-016-2452-5 (PMC4769575; doi:10.1186/s12864-016-2452-5)
Supplement: Additional file 1: — List of sequences of all NF11217 flanking sequence tags (FSTs) obtained from R0, R1 and BC1 F2 generations by TAIL-PCR. (PDF 62 kb) [file 12864_2016_2452_MOESM1_ESM.pdf]

Additional file 1: List of sequences of all NF11217 flanking sequence tags (FSTs) obtained from R_0, R_1 and BC_1F_2 Generations by TAIL-PCR.
(Veerappan et al. 2015, submitted to BMC Genomics)

*The signature refers to the *Tnt1* border sequence flanking *M. truncatula* genomic sequence and its location at the 5' or 3' end of the individual sequence, if listed.

I. NF11217 TAIL-PCR FSTs (R_0 generation) obtained from *Tnt1* mutants database (http://medicago-mutant.noble.org/mutant/)

```
 >NF11217_high_1        signature: TGATGATGTCCAT 3' end
CGTCGAGTGAGATGAACGAGGGTAAGGTTATCTTGTGGTGCAGTGTTTTGAAGGGAAAATATAGCCGAGG
GATACTTGAGGAGAGGCATTGTTGCTAAACCCTCAGATTATTTATTTGCAAAGTAATGGTTAGCACTTGG
ACTAGTATTAGTCATTCTGAGTCGGTGATGTGCGATCAGTTAATGTCTGGAGAAAACAAATCACAAATGA
CAGGTGGCAACATTATTTTAGCTGATTGCATGGTGCAAGTTCACGATGACATTAGAAAATGGATAATCTC
TGATTTGGTCGGTATGGAGATGGCAATTGGAAGGTGAATATTTTGATGGTGTAGATGTGAGAGTGTAGTG
TAGCCAGGGAAATAGACTTGGGAATTTTTCAGAGTCAAGTGCGTATAACATGCATGCTACGAGGGTATCA
TGACCATTTTCCAAGTTCAGTGTGGAGGAGAATGTGGAATATGGATGTTCTGGAATGAAAACAAATGCTT
TATTTGGCATCAGTTATTAGCTAGGTTTGTTTGGAATCACTTGGTTCCTATGCAAGTTTAGAGTGGATTG
CTAATAATTTGAATGTTGATGTGTTTTTAAACATTGGCTTTGCAAGTTGTTAGATCACCAAGCTTTAGGG
TGTTTTAAAGGGGATTCGGTTAATAGCCAAGGGGTTTGCAAATGGACTCTCTAATTGTGGTGTGGTGAGG
AGTCTTAATGGGAAGGATGTTCGAAGTATTGGTGGCAGGAGCTTGATCAGAAAGATTCGTCATTTGATTT
GTGAAAAGAGTGATGATGTCCAT

>NF11217_high_2 signature: TGATGATGTCCATCTCAT  3' end
TGACATTTTATATTCTTCCTAGTTTGGAATCAGTCATACGTTTTAAGAATATGTTGCCGTCCTTGGCCTT
CCTAAAAAAAATTGAGAGCAACACATAATCGTCCTTTTCCAAATCAAATAACGAATATGATTTTGTTCAA
AAAAAATAACGAATATGATCAAACACAAACTACAATATAATTTCATCAATCTATAGTTTTTTCCTTCTTA
TATACGATACAATTCAGCAGCGTAACCAACATATTTTCAATTACTTACTACAACAAAAAAAAAAGGTTAC
AATAACATGGTTGATTACCAAGGTTACATCTTACTTTTCATCATATGGCTAGTATCAACAATTTTTGTAA
AAGCCATACTCACAAGAAAATACAAGAAATCGAAGTTACCTCCAAGTCCATTATCTTTACCAATCATTGG
ACATCTTCACCTTATTGGTTCAATACCTCACCAAGGACTTCATAAACTTTCAACAAAATATGGACCTATA
ATACACCTTTTCCTAGGTTCCATGCCTTGTGTTGTAGCTTCCACACCAGAATCCGCTAAAGAGTTTCTTA
AAACTCACGAAACCTATTTCTCCAACCGTCCTCAAAGTTCAGCTGTTGATTATCTAACATATGGCTCACA
AGATTTTTCCTTTGCTCCATATGGACCTTATTGGAAATTTATCAAGAAAATATGCATGTCTGAACTTCTT
GGTGGTAACACATGATGATGTCCATCTCAT

>NF11217_high_3 signature: ATGAGATGGACATCATCA  5' end
ATGAGATGGACATCATCAAATCCCTCAACACTTGGCCAATTATTCTCCGTGTATATTTTCCATACTCCTT
CCTCATTCCCCAACTTTTCTGAAAAATGTTCTCTTAGGCCTATAAAAACTACCTTTTGATCAATCAGATC
CATATTCTCAGTTCACTAACCAAAACGCCATTCTTCTTTTGTTCTCTCCTCTCAAATTAAGTGCAATGGC
AACAAGAAAAAGCAAAGCCATAGGCATTGACCTTGGTACAAGCTACAGCTGCATTGCAGTGTGGCGAAAC
AATCGTGTTGAGATCATTCCAAACGACCAAGGGAACCGTGTCACCCCTTCTTACGTTGCCTTCACCGACA
CTGAAAGATTAATAGGCGATGCTGCCAAAAACCAATTAGCCAAAAATCCCCACAACACTGTTTTTGATGC
CAAACGTCTGATTGGCCGTCGATTCTCTGACCAAACAGTCCAACAAGACATAAAGCTTTGGCCTTTTAAA
GTTGTTCCGAACCACAAAGACAAACCCATGATTGTTGTCAATTACAAAGGCCAAGAGAAACATTTTTCAC
CTCAAGAGATATCTTCCATGGTGTTGTCTAAGTTAAAGGAAGTTGCTGAAACTTATTTGGGTCATGAAGT
GAACAATGCTGTTATCTCTGCTCCTCCTCA

>NF11217_high_4 signature: TGATGATGTCCATCTCA  3' end
AACCTAGAGTTTCTTGCTGATCAAAAGGATAGATTGGCACTTAACAAATTTACTAGTGGTGAATGTGAAA
TTGGAGTCTTGAAATTGTTAAGAGAATTTAGAGCTGTATCGAAAAGTTGTAATGACATATTTGTTGGTAA
AGATGGTAGAGTCATCCCCCGGATATTACCAACTGCACCTTTGATGGATAAATCTGCGGCGTATGGTAGG
GAGCATGAGATAGAGGAAATGACTGAATTTTTACTTTCAGACAGTTACAACCGGCTATCTGTAATCAGTA
TAGTGGGTCTGAAGGGGATGGGTAAGACAGCCCTTGCCCAGCTTGTTTACAATGACCACAGGATACAGGA
```

GCAGTTTGAATTTAGATCCTGGGTCCATGTTTCTGAATCTTTTGACTGTCTTCGTCTCATGAAAGAAATA
CGCGATCTTCAACCGCAACATTGGCAAGCAGGGAGTGAATATTTACTTGTTCTGGATGATGTCTGTATTA
AAAATAGTTATTATATGTCGGAGTATTTACTACCTCTTTGTAACCAGGGATCTTTTCGAGGCAAGATAAT
TGTGACGACAAATGATAAAGCAGTAGCATCAGTCATGAGATCGACCCAGATAATTTATTTAAGGCAATTG
GAGAAGAGTTGATGATGTCCATCTCA

>NF11217_high_5 signature: TGATGATGTCCATCTCAT 3' end
TCTTGAGGAGGAGCAGAGAATATGTGGGATGACATGTAGAAACCCAACAAAGACCCTACTGATGCTAATA
GTTGATCGTTCCTGTGTGTGTGGAAACGCGATTCACTTTGGTTGTAATTGCTAATTAACCATTTGTAACG
AAGGGAACGTGATTTGTTTGTTATTATTATGTATTTTTATTAAATAAATATTTGTTTTTTAATTTAAAAT
CTGCCACATGTCTTACATCCAAACTCTAAATGGAGTAAATAGAGTTTAGCCTAAATGGAGCATGCCCGAC
TCCAATACGCCAACCCAAAAATAGTCATAGCTGAACAACAGGAGAAAGAAATAAAGTAGAATCAACCGGA
GTACAATACGTTCAATCACATATGGTTCCTAACCCAACCACACCCTATACCCTCCATGGATACAATCACC
AAAAAAATTATACAAACAATGTAGATTTTGATGACCTATTTTCAAATTTAATTCCCAATTTTTTTAAAAT
GATTGAACTTTTCCCCATACATTTTGAGGCTGAATTTTAGGGAAGCTTTAAGTTATACCGTATGAAGGAA
GGAACTTGATGATGTCCATCTCAT

>NF11217_high_6 signature: TGAGATGGACATCATCA 5' end
TGAGATGGACATCATCATGTAACTCACCATGACAGAGCTAGTGTGTGGCATATTAATTTAGTGACCATGT
TTTCTGTCTCTAAAAAATATTTCTTCTTTCACTAATCTCCTACAGTGAGGTAAATATAATTTCCTCTTGG
ACCAAGCATAATCCCCTTATGTTAAATTTTTTCTTATAACAAAAAAATTAAATCAAAGAATGGCAAAGTG
GAAGATGAAAGGAAAAAAGCAGGAAAGAACAAGGGAACAGTTAGCTACTATACATTTATGTTAAAGTGGT
GAGCTTTCTTCCTGCTGTTCCCTAGTTATGTAGGCTTCAATATGAAAAATATGATGTGTCTCTCTCAACT
CAACCATCTAGAATCTAGCTTTGTCCAGTGCTATGAGTACTTTGCTGGATTGAGATTTAAATGTAGTGGA
CCATTAGTTTAATTCTTCCCCTATGTCTAACTCCAAGCTTATTCATATACGGGTAGCATTTTGGTGATAT
AGAATTGGTGATATTGGTGACATTTGAGGTTGGCCTGTGATTGGTTTGGGATCTTGGAGTTTGCTCCTC

>NF11217_high_7 signature: No signature
TCATCAACTGCTAACTATGCACAAGTTGAACATGGAGTTCCTCGAAAAGCAATAGAACCAAGTTACAAAA
TAATAACAAGAAAACCCTACAATACGATATTGATGTATAGTGAAGTCCTTAATATTATTGAGATTACATC
TCTTTGAATACAATTGTACATAAGTAGTTTACAAATATTGATGTATAGTGATGTACCTTCAAGATGACAA
CCTCTTTATATTATAGGTTCGATTCCTTTAAAGTAAATAGGTCTTCTGGTTGAGACTAATTCTAAGAGAA
TGTCTACTTGGAAACCTTTGTTGGATAATCAGGTAACACTTTTAAGCTCGTCGAGTAATAGATTGCCAGT
TTGGGTGGAAGTGTGATTTTTTTTAAAGGGACCTTTGTGGAATGATATCTAGTTGAGTGTTACTCCTTTG
AGGATTTGTTACCCCATGCTCTTTTAGGTCTCTATTCATTAAAAATAAAGTGATTGGTATGACTTTGAGA
GAGGGATATGGGTGGGTTTGGGATCTTAAGTGGAATCGC

>NF11217_high_8 signature: No signature
TCAATGAGATGGACATCATCAGCGTCGACATTGCAATCCACTTCACTGGCTACCAGTCTAATCCACCCTT
CTTGACTATGATCTGTCATTTCCAATGTTGTGGTATTCTCCTGGTTCTCTCCATTCTCAGACATACGCGG
ACCTACTGCCACTGAAGTAAGACATCACGCACCATACACACTGAGATACATGGTTGTGTTTCGACGTCAT
TCCACAACTTGTCGTTTTGTCGTTTCCAGATGCATCAAAGGGTCAGTACGAAATCAAAGAGTTGTTGGTG
TTGTAGAGATTCTACTAACTGAAAGTAAAGAGCTACAAAACCATATGTCGTATCCAAGTTGTCTTGAATA
ATGTTCTATAGACCTGCTACACTTCACACCTCCTCTAGTTTTGCACATCCGAAAAAGACATGTCATTCAT
TCTCATATGAGCTTTCACAAGGCACACAACAATCACAACAATGTACCCCGTGAGTTTAAAGCCCGTGTCT
CGTGGGTAAATACCTCTTCTCTGCTCCTCCTCA

>NF11217_high_9 signature: TGATGATGTCCATCTCA 3' end
TGAGCTTGTTGGTTCTAAATATTGTGTGTTATGTAGGTAAACAGTTTGTAAGCACCACATGGTCTTCTCT
TTAAGGTAATAGACGTATTCTAGATATTCTGCTTTAAGTGATCAACAAATCCTAAGCACCAAACAATTAT
TCGTTTATTCTTGACTTCATTTTGATAGTATATAATATGCACACTTTTGAAAACGTTAGAGGATAAAATA
TCTTTTTGTTATCATCAAAATTCGAAGATAACCCATATTTTGAATCTAACCATATTAACATGGTATACAT
TCAATCTAACATTCACATTCTTGTTAGATGAAACTCATATGGGTTACTCCACTCTATGTAGGCACCATTT
AAAAAGTGTGAGATTTAAATTAATTTTATCCAATAAAAAAGTGTTAGATATCGTATTTAAAAGAGAGTGT
TCATAACACTTCTCATTTTTATGATAATGATATATCTGACGGAAACCTTGCTCACATGAGCATCACACCT
GATGATGTCCATCTCA

>NF11217_high_10 signature: TGATGATGTCCATCTC 3' end
GTCGAGTGAGATGAATAAAGGATCACTTAAATTAGTACGGAGAGAGTATTAAAAAAACATGGGTGCTCGT
GTTTTTTTTAAATAGTAGTATAGTAGAAATTCATCATTTATATTCAATCATGTTTTTTAAACTAATGGTC
ATTCACAATGGAGTTTGACAATGGTTTTGGAAAAAAAGGAAGGAATGAATGGAAGAAATATATGACATTG
AGAGGGGAAAATATTGGGGTTTCATTTTTCTTCATAATACAAAGCCCTCATCATTTGAGGGAACTAAAAT
ATTGTATTATTGAGGAGGATTTTGCAGGGTTTACATGAATTTTTTAAATTTAATCTAACCAGTTATAATA
TTCTTAAAATTAAAAATATATTAATCATAAGCATTAACTTATCATTCTCTAAAAAATTAATCTATAAAAA
AAATACGAAAGATTTTACTATTTTCCCCTATATTTGATGATGTCCATCTC

>NF11217_high_11 signature: TGAGATGGACATCATCA 5' end
TGAGATGGACATCATCAGATTCAAGAACAATCAACCAATGTAAGCATTTATATTTAGCTATACACTTCAT
TACATATAATTCAACTGATATCCATGCCGAGCTGAAATCCTAAGGATACTCTTTTTCTGTGTCAGTATAG
TAACCGGTGTCCGTGTCCGTGTCAGTCCTTCATAATATTGATTTTGTTAATGATAAATGTACTACAGGAA
GGGCTTGAAACTGAGATAAACCTTGGAAGCCAAGCTAGTTGGTGGAAGCAACTCAAAACACTGACAAGGA
GATCATTTGTGAACATGTGTAGAGATGTTGGTTACTACTGGCTGAGGATTATGATCTATATCATTGTATC
TATATGTGTTGGAACCATCTATTTTGATGTTGGCTACGGCTACACTTCCATCTTGGCTCGTGGTGCCTGT
GGTGCATTTATATCAGGATTTATGACTTTCATCTCACTCGACC

>NF11217_high_12 signature: AGATGGACATCATCA 5' end
AGATGGACATCATCAGTTTGAACCCATATCTCAATGTTAAAAGTAAGAACTTAAGGTGTTAGCAGCCAAC
ACTCGGTGGATTTAGTCCCACATCGGATAGATAGGATTCTTGAGAAGAGTTTATAAGGAGTATGAGTTAG
GCCTGGATTTACGTGACATAAGGAACCTACAAATTATATGTGTGTGTCCCCACAAGACAGAACACTATCC
TATATATCTAAAGAACTTAGCAAACTTTGCCGGAAACTTTACGCCAAGCTTTAATTGAGAATATTCTAGA
CAACCAAATCCAAATCAGATAACTAAGTGTTAATGGGTTAGGATTTTCAATTTTCTACTTTGTTTAACCC
AATGTGACGGGGACTTGGGTATTATGGGCTGCCCAAGTTCCACATCGAGTAGTATGGAACGTTCCGTGAG
TAGCTTAGTTCTTCCCCTTTGCTTCTCC

>NF11217_high_13 signature: TGAGATGGACATCATCA 5' end
TGAGATGGACATCATCATATATTTTAGATCGAATTTACAATATAAAATTCTTCTACTATGGAGAATTGGA
AAATATTATAATCAATCTATAATATCACATAGATATATTAAAAAATATAACTGACCATTTCCATTGATCA
CCATTGAGACTTATATACTTCATCTATTATTTCATAATACAACTTGCAATGTCATGCATAATAGAATTTA
GGCTTAAATAAGAAAAAGGTTCATGTAAGTTCGCGCATTTTTTGTTTTAGTTATTGTATCTTTTTTTGTT
GCGAAACAACCCCTGTATTTACTTAATATTTTGACATTCGTCCCTGCCGTCCATTTCCGTAAAAAAAAAC
GCTTCTCTGGCTAACGGACTAATATGTGGCATATGATGAGCTGACAATTGGCTATTAGAGGGACTAAAAC
CAAAAATGCAAATTTACATAGGGAC

>NF11217_high_14 signature: TGATGATGTCCATCT 3' end
TGAGGAGGAGCAGAGAAGTTGTGGTCACATAAGATGGTTGGATTAGTATTTGGATGTGCTTTCCTGTCCT
TGCTGTTCTAGATCGATACCTCTGTCTTCTCTTCAGAACTCAGATTGTTATTTTTACCTCTATATATTAT
TATTATTTTCTCGATGTGATATGGGAGTTTATTACTACATACATTGTTTGCATTCTTGAGACATTCATTT
CAGGGGAGGTTTTGTATTAATTGAGGACACTTATTTTGTATCTCACTATTACTATTATAGTTTATCCATC
ATTCGTTGTTTTCTTTGAATATTTTATTGATTACATTTTTCTGTTAATTCTTCTAATCCTAACTTTTCAG
CCAATGTTTCTATTTCTATTTACATTCACGTAGTTCTGTAACTTAGGGAGACTCTACAACAGGAACCATT
AGATTTGATGATGTCCATCT

>NF11217_high_15 signature: TGATGATGTCCATCTCAT 3' end
CTAGTGGAGAAGCAGAGATAAACGGATTGACCATTTGGGATGTTAAAGAACGAATGCAAAACAACAAAAT
TATAATTGAAAAGTTATGTGGTCAAATTTAATATGAACATTCTTTTATTACTTTTATCTTGCAATATGGA
AAACGAACAATCATGAGTGTATTTTTCCTAAAACCTGAGAATGAATTTACGAAATATTGTTTATATCTTC
TTCGTAGTTATTTATTAAAGTAATAAAAATAATTATGTTTAAATAATAGGTAAAGTGAATCATAGTATCA
AGTTTTACTTTAGAAAATATTATTTTTAATTTGATAAGTTGAACAATATGGGGTGGTAAAGTGGTTTTGT
TTTCTTTAATGATTTTTAAAATTTTTAATATCAGCTACAATCATAAGAATTCATGATGATGTCCATCTCA
T

>NF11217_high_16 signature: TGATGATGTCCATCTC 3' end
GTCGAGTGAGATGAAACTCATGCAAAGGCCAAGAATGTATACTGAAATTTTCTCAATGTCATCTTTCAAG

TCCTCAGAAAGGCCAAGAATGCATACCTTTTGGATTGTGATAGTGATATCATTTGTATCAGATAGCCATG
GTGAGGGAGGAGAAGAAATGAGAAATGTGGTAGCTGTAGTCGATCATAATGGGAGTAATGAGATTTATAT
ATACCATTTCTTTTAAGGAAGTGACAAAAAAAACTTGAGAGTGTGTTTGGATGAGGGAATATTTTGAGGC
AATGTAATGTTTTGAGGGAATTCAATTATTTTGAGGTGGATTCAAACAATAGAATTCACCTCAAAGTAAT
TGAATTCGCTCAAAACATTACATTCCCTCAAAACTGATGATGTCCATCTC

>NF11217_high_17 signature: TGATGATGTCCATCTCA 3' end
AGATGGACATCATCAAATAAAACAACATTAACCACCTTTTTCTTGAATCAAAACTATGGTATCATTGCTC
AATGCTTGAAACTTAAGAATTTTATTGTACCATTTAAACCAGATCTGATGTCTTTGAAAACTTTGTGGCA
CAACAGTATCGTTTCTTGCTGACAGATCGGGAGTTTGATATTGGACTTAAATATTCGATTCGCCTCCAAA
AGTCTTCAAGAACACTGCTGCACACATATGCAGGACTAGGAGGTACCTCAGTGTAGTTCTCCTAAAGAGC
ATGTAGCAGAAAATCAAATGACCGTTTATACAACACCTCTGTATGAAATATAAAGGTCATCATGGTCAAC
AGGCAAAAATAGTATGAACGAATTGATGATGTCCATCTCA

>NF11217_high_18 signature: TGATGATGTCCATCTCATT 3' end
TGAGGAGGAGCAGAGACAGGCAAATTTGCAGACTTTCTTTGAGAAGGCTCTTCAGGATTCTTTTATTGTG
GAACTTCTTTCCCGCAAAATCGAATCCATGGATTTGGCATCTGATAATAAGAAAAGGCGATTGTCTCAGG
TTGATCGCATACAGCCTGTTGTCGAAGGTATTTTGCTTGACAATCCTTGCAGTTTCATACTCGAATTTGG
TAATGTTTTCTATCAAGATATCTCAAATAAACTAAGATTAGAATTATCACTAGCTGACTTAGATATGAAC
TTGATATCAGGTAGCACACAGGGTTCAAATGAAGATGAGGATGATGATGTCCATCTCATT

>NF11217_high_19 signature: TGATGATGTCCATCTCAT 3' end
TGAGGAGGAGCACAGACTTGCATCTGAACGACGAGTGGGTCCACCAATACCTAACAGAAAAGTTCAATAT
GCTGATCTTCATATAGATAAGCGTACAAGAGAGTCACCCTGTAATGATTTATCTGCTACCAAATCTGTAC
CTGTTGCTGTTTCAGGTAGGATACTTTTCTAGTTATAATGGACTTTTCGAATTGTCTCTGTTTTTTTTCT
CGTAGCATCTCAATATATATGTGGTAAAGCATCTTGGTATGAATTGTGATATTGTCATTTGAGCCTGTCA
GATTTGAACCAATCATCTCTAATCTTATTGATGATGTCCATCTCAT

>NF11217_high_20 signature: TGATGATGTCCATCTC 3' end
GTCAGATTTTTGTGTGGGTGTCAAGGGGTGTTTATGTATCATAACTCTTAAAAAAAAACGAACCTTCCAA
CCCCACGACACCTATAATTAGCCAGAAGCAGTCATGCGAGGCAACACTAACATCAGCTGAAACAAAGAAG
ACATACAAGGTAAACTTGATATTCTGAACCTTTCTATTATGAAATTCTCTTCCAATTTCCTACTCATATC
TTTATTCTTCCCTTTATATTTTGTAGATTATTTATTATATACTGTTTCAAGTTGATGTTCAATGATGGGA
GCAAGACTAAATTGTATATCTTGATGATGTCCATCTC

>NF11217_high_21 signature: TGATGATGTCCATCTC 3' end
TGAGGAGGAGCACAGAGAGCTCTGTTGCTGTAGTGTCTGAGCCACAAAATGCCGTCCAATCATTTACTAC
CATGCCAGATGCTATTACTTCACTACCGCGATTGACAGTCCCTGCAACCAACGGAATTTGAAGAAGTGTA
GAGAGTTCATCCAAGTCTTCAACAGAAGTATGAGGGTGGACCTGCATTCAGTGTATATGATCAAAAGATA
GAACATTTATTCAAGTTGAAACATTCAAAAGGACATGTCTTGCACCTACCAAACCACCTCTGTTGGACAG
AGCACAGTAACTGCCAGAAAGTGATGATGTCCATCTC

>NF11217_high_22 signature: TGATGATGTCCATCTC 3' end
AAACTACTATATTCAAGCTGCAAGAACATTGATCATGTTATATCGCTATTTATTTTTATTTTTTTGCCAC
ATTAGAGTGATGTTGTGGAAAGTTCTTGTTCCAGCTTAACATTTTTTTGCTTTTTCAGACGATCTAAGCC
CTTTTTAAACAATCACTTTCCACGTTTGGCTTAGGCGTACTATGCAGTTTCTCAAAAGAGGTTTAATCAA
AGTTTGGTAGTGCCTATAAATGGATAAGATCATTAAGCAAATTGTCAACTTTTTTTTCTGAAACTTTTCA
AAAGTGATGATGTCCATCTC

>NF11217_high_23 signature: AATGAGATGGACATCATCA 5' end
AATGAGATGGACATCATCAGTTGATAGAGTGTTCATCGAGGCCACAGAAGAAATCAACTGCACATCACAT
GAAGACGAGGCCCAGAAAATCATCACTGTCAGATAGGAATCGGAAACCAACAGAGTATGCTCCGTTGCCA
CCACTGCCTCCTGATTTTACAATTGTCATTTCTGCTGATGCATCTTCAACGGCTACTGTTGTTCCTCCAC
CCCCTACTCCTTCTACTTGATTCGGTACTTTTTTTTATTTTATTTTATTTTTAAAGTATCTGTGCTGCTC
CTCAAGAT

>NF11217_high_24	signature: TGATGATGTCCATCTCATT	3' end
AAAAGAGGTAAAACTGCATTTTTATGACCGTGTTCAGTTTAGTTCATGTTGTAGCAGTTTAGATTGTAAA
TGTGAAAGCTGGTGTAGTTTTCATAATCCACTAGTAGCCTTGTTCTCTAATAGTTTTCAAAGACTGCTTC
ATCTAGAATTATTTCTTACTGATATAACTCTTGAAACTTAGGTTACGAAAAGGGCATGCAAATCTGTTAA
ATTTTAACAAGTCTTGCTTGTTTTTGTTATGCCCTGAGGTTGTAGGACTTTATTGATGATGTCCATCTCA
TT

>NF11217_high_25	signature: No signature
TGAGGAGGAGCACAGACACAACTTTACCAACTTTTTCCTAGTGAGAGAGGAGAGAGTCAATTCAATATGG
TGCAAGGCTTAATTTTGAAGGGGTTCAAGACAATGAAAATATAGTAAAATAGGTGTAAATATGAAAAGTT
GAGAAGGTGCAATTGAACCCCTGGCCACAACGTGGCTCCGACAATGTGTCTAATATAAATCCCGACATTT
TTTTGATTAAAATAGGTTATCAATCATGGTCTCTCGTTGGATGAAATGAAGGAAGCAATGAGTGTTTT

>NF11217_high_26	signature: ATGAGATGGACATCATCA 5' end
ATGAGATGGACATCATCATAACTAAATAAATTACATATAATGGAGAGAACCTATAAAAAAAATACCGTAA
CTTATTTGATCATATACTCTAAAAGACATTTATTCCTTCTCAAATTTTTTTTTTTGGTACATCCTTCTCA
ATATTGTTATTAGTTATAAGGTGCAAAGAGAGTTAACTAGTAAAATTAATTAAGAAAATTGCATCAGTTA
GCTGATTGCTGATTATATGGTGTTACTATTAATATTCACCTTCATCCTTTGCTTCTCCTCT

>NF11217_high_27	signature: No signature
ATTCCTCCAACCTACCCATTGCATAAGGATGATAAGTAACCCTAGTACCACTTTTCACTTTTGTACCATC
TGGTAAAACATCATCATCCAAACAAAACTTTGAATCAAATTGAATTGGTGGATATAGTCTCATACTTTCA
TGTGCTGCTGCTTGTAAATAATGCAATTTCCTAAGTTGTTCAAAATTCGTTACACCAAAAATATTGTTTT
CATCATCATGTGGTCCAATTACTCTCTCTGCTTCTCCACT

>NF11217_high_28	signature: No signature
CTGAAAGTAAAAATTTGATTATTTCCTCTTTCTCACGTTCTCTACCATATATGATAGATTCATCCATGAA
TGATGAAGTCGCAAAATCATTGGCTCCATTGTCAAAATAACTAGTTAATTCAACAAGTTTCAATCTAGAT
TTTTCCTTTGCAAGAAACTCTAGCCTCCTGACCAATACTTTAATCCTAGATTGAAATCGATTGATAGAAG
CTGAAAGAAAGCATTGTATCTTTCCCTTTGCTTCTCCTCT

>NF11217_high_29	signature: No signature
TAACACACACTAAGTTTGATGAAATCATGGTGGATTCTGGGATTCTTTTGAAGCTCGAAGTGTAATTTTT
GCTAAATTTGCGGCGACACATTGCACTAAGTTTGACGAAATCAATGTGGTAATTGAGATTTTGTTGAAGT
TTCAAAGTGCAGTTTTTTGCTAAAATCACGGTGGCACAACTTAAATATTCCAAACACACAACTCCAATGT
TTGGTATTCCTTTCTCACGTCCCTATGCTACTCCACT

>NF11217_high_30	signature: No signature
TAACTGATTAAATAATATGCAATTCATTCGCACTTCTTAGTCTATAAGGTCTAACTCAATCAATAAAATT
CTCAAAATTGTTATACCGAATATTGTGACTGCAGTTTAAAACCTCAGTTACTCCACTTATGTGTGTGAGT
TTTCGATGGATATTGTCATGTTGTCTATCTACCCAAAAAAAAAAAAATTCGCACTTTAGACCGTGTAAAA
ATATTTGCGTTAGAGAATGATGTCCATCTCATTGC

>NF11217_high_31	signature: TGATGATGTCCATCTC	3' end
AGGGTTTGTATTGGTAAAGAAATGGCTTTGATGGAACTTAAAAGTGTGGCAATTTCTTTGCTTAGAAAGT
TTCATATTGAATTGGAAATGGAAGATAGAACAATGTTTCATGGTAACCCAAGATTCTCTCCTGGACTAAC
TGCCACTTTTGCTTTTGGTCTTCCTGTATTTGTTCGTCCAAGAGGAACCAAGTGAATAATCCGTACTACT
ATATATATGTATCTGATGATGTCCATCTC

>NF11217_high_32	signature: No signature
AGAGAAGCTCAAACGACTATGGTAGAAGATCTGAAGAAAGGTCAACCATAAGTCAACATATTGAATCAGA
AGAAAATGACGTCTCTTAGAAGCTGAAGATTTGCTTCATTAGACTATGTGGAGATCATCAGACTCAGACG
ACCTAGCAGACGCAGAAGACCCCATCAAACTTGGAAGACCTTCAGACTTTAGAAGCACTCGAGGGACCCA
AACTCCATGTCAAATTTGTCGTC

>NF11217_high_33	signature: AATGAGATGGACATCATCA	5' end
AATGAGATGGACATCATCAGATCTGCCATGTCCTTTCGCCGTGTTTATTCATTTTTGCCGTCAGCTCAAC

TGCTACTGTGATTTTCTTGATTGATACTCATACATTCTTTATCAAAGTAGAGTCATTTAAAATCTTATTA
TCTGCATATGATTTGTATTTTTATCTACATGTATTAACACTTGAGTTTCCCCATAAAAACAATCTCTTCT
GTGCTCCTCCTCAAGATC

>NF11217_high_34 signature: TGATGATGTCCATCTC 3' end
AGGTATGAGACTGCATGGTTGAAGGAAGGCTTATAAGAATTGTTTGGTACTACCTATATCAACATGATGC
ATCTTCTTTTCGGTAGCACATTTCATAAGAACTCCACAATTACGCGTGCTTGACTTGGAGAAATTTTGGG
ATGTGTGACCTACCGAGAAGTTTCCCGGTAAGCATGCGTGTGAAAAGACTCGTGTTGGTTTATAGGTGAT
GATGTCCATCTC

>NF11217_high_35 signature: TGAGATGGACATCATCA 5' end
TGAGATGGACATCATCAAAATGTAGTTGGCATTTGGCAATTTTTTAAGGGTTTCCAACTGACTCAACCTT
CATTTTTAACACATTTCTCTTTTTTTACTCCTAAAGAAATTCTAGCATCTCTAGCAATTTTCCTTTCCTA
GTTTCAAATGGACTGGTAAAAGCTGAGAAGCTTACTACCTTGCTAGTAGTACATTCAACTTCTCTGCTCC
TCCTCAAGA

>NF11217_high_36 signature: TGATGATGTCCATCTCATT 3' end
CGATCTTGAGGAGGAGCACAGAAGGAACAACAATTCATGAAAAAGAAAATCAATTTCATGGACAAAAGAT
AATGAAGAAAAACACCCAAATTCAAATTTTACAAAGAATCAATTCAAGAAAAGATGTTAGGGGAACTTTC
GTCTTTTTGACCAAAATTTATGTCATTCTCTCGCTTTTTTCAGTGATGATGTCCATCTCATT

>NF11217_high_37 signature: TGATGATGTCCATCTC 3' end
ATCTAGTGGAGTAGCAAAGGTAGTATAAATACAAACATGTTTTCTCATTCCATATTCATTTTCCTTTTCC
ACAAAGAAACATCAATACAACAAGTTTTTTTCCATTCATCATTCTTTAATTCTTTCATTATATTCTATTC
TACACTTCATTTAGTTTATTGTTTGGGTTCAATTCATAATAACATTTGATGATGTCCATCTC

>NF11217_low_1 signature: TGATGATGTCCAT 3' end
CGTCGAGTGAGATGAACGAGGGTAAGGTTATCTTGTGGTGCAGTGTTTTGAAGGGAAAATATAGCCGAGG
GATACTTGAGGAGAGGCATTGTTGCTAAACCCTCAGATTATTTATTTGCAAAGTAATGGTTAGCACTTGG
ACTAGTATTAGTCATTCTGAGTCGGTGATGTGCGATCAGTTAATGTCTGGAGAAAACAAATCACAAATGA
CAGGTGGCAACATTATTTTAGCTGATTGCATGGTGCAAGTTCACGATGACATTAGAAAATGGATAATCTC
TGATTTGGTCGGTATGGAGATGGCAATTGGAAGGTGAATATTTTGATGGTGTAGATGTGAGAGTGTAGTG
TAGCCAGGGAAATAGACTTGGGAATTTTTCAGAGTCAAGTGCGTATAACATGCATGCTACGAGGGTATCA
TGACCATTTTCCAAGTTCAGTGTGGAGGAGAATGTGGAATATGGATGTTCTGGAATGAAAACAAATGCTT
TATTTGGCATCAGTTATTAGCTAGGTTTGTTTGGAATCACTTGGTTCCTATGCAAGTTTAGAGTGGATTG
CTAATAATTTGAATGTTGATGTGTTTTTAAACATTGGCTTTGCAAGTTGTTAGATCACCAAGCTTTAGGG
TGTTTTAAAGGGGATTCGGTTAATAGCCAAGGGGTTTGCAAATGGACTCTCTAATTGTGGTGTGGTGAGG
AGTCTTAATGGGAAGGATGTTCGAAGTATTGGTGGCAGGAGCTTGATCAGAAAGATTCGTCATTTGATTT
GTGAAAAGAGTGATGATGTCCAT

>NF11217_low_10 signature: TGATGATGTCCATCTC 3' end
GTCGAGTGAGATGAATAAAGGATCACTTAAATTAGTACGGAGAGAGTATTAAAAAAACATGGGTGCTCGT
GTTTTTTTTAAATAGTAGTATAGTAGAAATTCATCATTTATATTCAATCATGTTTTTTAAACTAATGGTC
ATTCACAATGGAGTTTGACAATGGTTTTGGAAAAAAAGGAAGGAATGAATGGAAGAAATATATGACATTG
AGAGGGGAAAATATTGGGGTTTCATTTTTCTTCATAATACAAAGCCCTCATCATTTGAGGGAACTAAAAT
ATTGTATTATTGAGGAGGATTTTGCAGGGTTTACATGAATTTTTTAAATTTAATCTAACCAGTTATAATA
TTCTTAAAATTAAAAATATATTAATCATAAGCATTAACTTATCATTCTCTAAAAAATTAATCTATAAAAA
AAATACGAAAGATTTTACTATTTTCCCCTATATTTGATGATGTCCATCTC

>NF11217_low_11 signature: TGAGATGGACATCATCA 5' end
TGAGATGGACATCATCAGATTCAAGAACAATCAACCAATGTAAGCATTTATATTTAGCTATACACTTCAT
TACATATAATTCAACTGATATCCATGCCGAGCTGAAATCCTAAGGATACTCTTTTTCTGTGTCAGTATAG
TAACCGGTGTCCGTGTCCGTGTCAGTCCTTCATAATATTGATTTTGTTAATGATAAATGTACTACAGGAA
GGGCTTGAAACTGAGATAAACCTTGGAAGCCAAGCTAGTTGGTGGAAGCAACTCAAAACACTGACAAGGA
GATCATTTGTGAACATGTGTAGAGATGTTGGTTACTACTGGCTGAGGATTATGATCTATATCATTGTATC
TATATGTGTTGGAACCATCTATTTTGATGTTGGCTACGGCTACACTTCCATCTTGGCTCGTGGTGCCTGT

GGTGCATTTATATCAGGATTTATGACTTTCATCTCACTCGACC

>NF11217_low_12 signature: AGATGGACATCATCA 5' end
AGATGGACATCATCAGTTTGAACCCATATCTCAATGTTAAAAGTAAGAACTTAAGGTGTTAGCAGCCAAC
ACTCGGTGGATTTAGTCCCACATCGGATAGATAGGATTCTTGAGAAGAGTTTATAAGGAGTATGAGTTAG
GCCTGGATTTACGTGACATAAGGAACCTACAAATTATATGTGTGTGTCCCCACAAGACAGAACACTATCC
TATATATCTAAAGAACTTAGCAAACTTTGCCGGAAACTTTACGCCAAGCTTTAATTGAGAATATTCTAGA
CAACCAAATCCAAATCAGATAACTAAGTGTTAATGGGTTAGGATTTTCAATTTTCTACTTTGTTTAACCC
AATGTGACGGGGACTTGGGTATTATGGGCTGCCCAAGTTCCACATCGAGTAGTATGGAACGTTCCGTGAG
TAGCTTAGTTCTTCCCCTTTGCTTCTCC

>NF11217_low_13 signature: TGAGATGGACATCATCA 5' end
TGAGATGGACATCATCATATATTTTAGATCGAATTTACAATATAAAATTCTTCTACTATGGAGAATTGGA
AAATATTATAATCAATCTATAATATCACATAGATATATTAAAAAATATAACTGACCATTTCCATTGATCA
CCATTGAGACTTATATACTTCATCTATTATTTCATAATACAACTTGCAATGTCATGCATAATAGAATTTA
GGCTTAAATAAGAAAAAGGTTCATGTAAGTTCGCGCATTTTTTGTTTTAGTTATTGTATCTTTTTTTGTT
GCGAAACAACCCCTGTATTTACTTAATATTTTGACATTCGTCCCTGCCGTCCATTTCCGTAAAAAAAAAC
GCTTCTCTGGCTAACGGACTAATATGTGGCATATGATGAGCTGACAATTGGCTATTAGAGGGACTAAAAC
CAAAAATGCAAATTTACATAGGGAC

>NF11217_low_14 signature: TGATGATGTCCATCT 3' end
TGAGGAGGAGCAGAGAAGTTGTGGTCACATAAGATGGTTGGATTAGTATTTGGATGTGCTTTCCTGTCCT
TGCTGTTCTAGATCGATACCTCTGTCTTCTCTTCAGAACTCAGATTGTTATTTTTACCTCTATATATTAT
TATTATTTTCTCGATGTGATATGGGAGTTTATTACTACATACATTGTTTGCATTCTTGAGACATTCATTT
CAGGGGAGGTTTTGTATTAATTGAGGACACTTATTTTGTATCTCACTATTACTATTATAGTTTATCCATC
ATTCGTTGTTTTCTTTGAATATTTTATTGATTACATTTTTCTGTTAATTCTTCTAATCCTAACTTTTCAG
CCAATGTTTCTATTTCTATTTACATTCACGTAGTTCTGTAACTTAGGGAGACTCTACAACAGGAACCATT
AGATTTGATGATGTCCATCT

>NF11217_low_15 signature: TGATGATGTCCATCTCAT 3' end
CTAGTGGAGAAGCAGAGATAAACGGATTGACCATTTGGGATGTTAAAGAACGAATGCAAAACAACAAAAT
TATAATTGAAAAGTTATGTGGTCAAATTTAATATGAACATTCTTTTATTACTTTTATCTTGCAATATGGA
AAACGAACAATCATGAGTGTATTTTTCCTAAAACCTGAGAATGAATTTACGAAATATTGTTTATATCTTC
TTCGTAGTTATTTATTAAAGTAATAAAAATAATTATGTTTAAATAATAGGTAAAGTGAATCATAGTATCA
AGTTTTACTTTAGAAAATATTATTTTTAATTTGATAAGTTGAACAATATGGGGTGGTAAAGTGGTTTTGT
TTTCTTTAATGATTTTTAAAATTTTTAATATCAGCTACAATCATAAGAATTCATGATGATGTCCATCTCA
T
>NF11217_low_16 signature: TGATGATGTCCATCTC 3' end
GTCGAGTGAGATGAAACTCATGCAAAGGCCAAGAATGTATACTGAAATTTTCTCAATGTCATCTTTCAAG
TCCTCAGAAAGGCCAAGAATGCATACCTTTTGGATTGTGATAGTGATATCATTTGTATCAGATAGCCATG
GTGAGGGAGGAGAAGAAATGAGAAATGTGGTAGCTGTAGTCGATCATAATGGGAGTAATGAGATTTATAT
ATACCATTTCTTTTAAGGAAGTGACAAAAAAAACTTGAGAGTGTGTTTGGATGAGGGAATATTTTGAGGC
AATGTAATGTTTTGAGGGAATTCAATTATTTTGAGGTGGATTCAAACAATAGAATTCACCTCAAAGTAAT
TGAATTCGCTCAAAACATTACATTCCCTCAAAACTGATGATGTCCATCTC

>NF11217_low_17 signature: TGATGATGTCCATCTCA 3' end
AGATGGACATCATCAAATAAAACAACATTAACCACCTTTTTCTTGAATCAAAACTATGGTATCATTGCTC
AATGCTTGAAACTTAAGAATTTTATTGTACCATTTAAACCAGATCTGATGTCTTTGAAAACTTTGTGGCA
CAACAGTATCGTTTCTTGCTGACAGATCGGGAGTTTGATATTGGACTTAAATATTCGATTCGCCTCCAAA
AGTCTTCAAGAACACTGCTGCACACATATGCAGGACTAGGAGGTACCTCAGTGTAGTTCTCCTAAAGAGC
ATGTAGCAGAAAATCAAATGACCGTTTATACAACACCTCTGTATGAAATATAAAGGTCATCATGGTCAAC
AGGCAAAAATAGTATGAACGAATTGATGATGTCCATCTCA

>NF11217_low_18 signature: TGATGATGTCCATCTCATT 3' end
TGAGGAGGAGCAGAGACAGGCAAATTTGCAGACTTTCTTTGAGAAGGCTCTTCAGGATTCTTTTATTGTG
GAACTTCTTTCCCGCAAAATCGAATCCATGGATTTGGCATCTGATAATAAGAAAAGGCGATTGTCTCAGG
TTGATCGCATACAGCCTGTTGTCGAAGGTATTTTGCTTGACAATCCTTGCAGTTTCATACTCGAATTTGG

TAATGTTTTCTATCAAGATATCTCAAATAAACTAAGATTAGAATTATCACTAGCTGACTTAGATATGAAC
TTGATATCAGGTAGCACACAGGGTTCAAATGAAGATGAGGATGATGATGTCCATCTCATT

>NF11217_low_19 signature: TGATGATGTCCATCTCAT 3' end
TGAGGAGGAGCACAGACTTGCATCTGAACGACGAGTGGGTCCACCAATACCTAACAGAAAAGTTCAATAT
GCTGATCTTCATATAGATAAGCGTACAAGAGAGTCACCCTGTAATGATTTATCTGCTACCAAATCTGTAC
CTGTTGCTGTTTCAGGTAGGATACTTTTCTAGTTATAATGGACTTTTCGAATTGTCTCTGTTTTTTTTCT
CGTAGCATCTCAATATATATGTGGTAAAGCATCTTGGTATGAATTGTGATATTGTCATTTGAGCCTGTCA
GATTTGAACCAATCATCTCTAATCTTATTGATGATGTCCATCTCAT

>NF11217_low_2 signature: TGATGATGTCCATCTCAT 3' end
TGACATTTTATATTCTTCCTAGTTTGGAATCAGTCATACGTTTTAAGAATATGTTGCCGTCCTTGGCCTT
CCTAAAAAAAATTGAGAGCAACACATAATCGTCCTTTTCCAAATCAAATAACGAATATGATTTTGTTCAA
AAAAAATAACGAATATGATCAAACACAAACTACAATATAATTTCATCAATCTATAGTTTTTTCCTTCTTA
TATACGATACAATTCAGCAGCGTAACCAACATATTTTCAATTACTTACTACAACAAAAAAAAAAGGTTAC
AATAACATGGTTGATTACCAAGGTTACATCTTACTTTTCATCATATGGCTAGTATCAACAATTTTTGTAA
AAGCCATACTCACAAGAAAATACAAGAAATCGAAGTTACCTCCAAGTCCATTATCTTTACCAATCATTGG
ACATCTTCACCTTATTGGTTCAATACCTCACCAAGGACTTCATAAACTTTCAACAAAATATGGACCTATA
ATACACCTTTTCCTAGGTTCCATGCCTTGTGTTGTAGCTTCCACACCAGAATCCGCTAAAGAGTTTCTTA
AAACTCACGAAACCTATTTCTCCAACCGTCCTCAAAGTTCAGCTGTTGATTATCTAACATATGGCTCACA
AGATTTTTCCTTTGCTCCATATGGACCTTATTGGAAATTTATCAAGAAAATATGCATGTCTGAACTTCTT
GGTGGTAACACATGATGATGTCCATCTCAT

>NF11217_low_20 signature: TGATGATGTCCATCTC 3' end
GTCAGATTTTTGTGTGGGTGTCAAGGGGTGTTTATGTATCATAACTCTTAAAAAAAAACGAACCTTCCAA
CCCCACGACACCTATAATTAGCCAGAAGCAGTCATGCGAGGCAACACTAACATCAGCTGAAACAAAGAAG
ACATACAAGGTAAACTTGATATTCTGAACCTTTCTATTATGAAATTCTCTTCCAATTTCCTACTCATATC
TTTATTCTTCCCTTTATATTTTGTAGATTATTTATTATATACTGTTTCAAGTTGATGTTCAATGATGGGA
GCAAGACTAAATTGTATATCTTGATGATGTCCATCTC

>NF11217_low_21 signature: TGATGATGTCCATCTC 3' end
TGAGGAGGAGCACAGAGAGCTCTGTTGCTGTAGTGTCTGAGCCACAAAATGCCGTCCAATCATTTACTAC
CATGCCAGATGCTATTACTTCACTACCGCGATTGACAGTCCCTGCAACCAACGGAATTTGAAGAAGTGTA
GAGAGTTCATCCAAGTCTTCAACAGAAGTATGAGGGTGGACCTGCATTCAGTGTATATGATCAAAAGATA
GAACATTTATTCAAGTTGAAACATTCAAAAGGACATGTCTTGCACCTACCAAACCACCTCTGTTGGACAG
AGCACAGTAACTGCCAGAAAGTGATGATGTCCATCTC

>NF11217_low_22 signature: TGATGATGTCCATCTC 3' end
AAACTACTATATTCAAGCTGCAAGAACATTGATCATGTTATATCGCTATTTATTTTTATTTTTTTGCCAC
ATTAGAGTGATGTTGTGGAAAGTTCTTGTTCCAGCTTAACATTTTTTTGCTTTTTCAGACGATCTAAGCC
CTTTTTAAACAATCACTTTCCACGTTTGGCTTAGGCGTACTATGCAGTTTCTCAAAAGAGGTTTAATCAA
AGTTTGGTAGTGCCTATAAATGGATAAGATCATTAAGCAAATTGTCAACTTTTTTTTCTGAAACTTTTCA
AAAGTGATGATGTCCATCTC

>NF11217_low_23 signature: AATGAGATGGACATCATCA 5' end
AATGAGATGGACATCATCAGTTGATAGAGTGTTCATCGAGGCCACAGAAGAAATCAACTGCACATCACAT
GAAGACGAGGCCCAGAAAATCATCACTGTCAGATAGGAATCGGAAACCAACAGAGTATGCTCCGTTGCCA
CCACTGCCTCCTGATTTTACAATTGTCATTTCTGCTGATGCATCTTCAACGGCTACTGTTGTTCCTCCAC
CCCCTACTCCTTCTACTTGATTCGGTACTTTTTTTTATTTTATTTTATTTTTAAAGTATCTGTGCTGCTC
CTCAAGAT

>NF11217_low_24 signature: TGATGATGTCCATCTCATT 3' end
AAAAGAGGTAAAACTGCATTTTTATGACCGTGTTCAGTTTAGTTCATGTTGTAGCAGTTTAGATTGTAAA
TGTGAAAGCTGGTGTAGTTTTCATAATCCACTAGTAGCCTTGTTCTCTAATAGTTTTCAAAGACTGCTTC
ATCTAGAATTATTTCTTACTGATATAACTCTTGAAACTTAGGTTACGAAAAGGGCATGCAAATCTGTTAA
ATTTTAACAAGTCTTGCTTGTTTTTGTTATGCCCTGAGGTTGTAGGACTTTATTGATGATGTCCATCTCA
TT

>NF11217_low_25 signature: No signature
TGAGGAGGAGCACAGACACAACTTTACCAACTTTTTCCTAGTGAGAGAGGAGAGAGTCAATTCAATATGG
TGCAAGGCTTAATTTTGAAGGGGTTCAAGACAATGAAAATATAGTAAAATAGGTGTAAATATGAAAAGTT
GAGAAGGTGCAATTGAACCCCTGGCCACAACGTGGCTCCGACAATGTGTCTAATATAAATCCCGACATTT
TTTTGATTAAAATAGGTTATCAATCATGGTCTCTCGTTGGATGAAATGAAGGAAGCAATGAGTGTTTT

>NF11217_low_26 signature: ATGAGATGGACATCATCA 5' end
ATGAGATGGACATCATCATAACTAAATAAATTACATATAATGGAGAGAACCTATAAAAAAAATACCGTAA
CTTATTTGATCATATACTCTAAAAGACATTTATTCCTTCTCAAATTTTTTTTTTTGGTACATCCTTCTCA
ATATTGTTATTAGTTATAAGGTGCAAAGAGAGTTAACTAGTAAAATTAATTAAGAAAATTGCATCAGTTA
GCTGATTGCTGATTATATGGTGTTACTATTAATATTCACCTTCATCCTTTGCTTCTCCTCT

>NF11217_low_27 signature: No signature
ATTCCTCCAACCTACCCATTGCATAAGGATGATAAGTAACCCTAGTACCACTTTTCACTTTTGTACCATC
TGGTAAAACATCATCATCCAAACAAAACTTTGAATCAAATTGAATTGGTGGATATAGTCTCATACTTTCA
TGTGCTGCTGCTTGTAAATAATGCAATTTCCTAAGTTGTTCAAAATTCGTTACACCAAAAATATTGTTTT
CATCATCATGTGGTCCAATTACTCTCTCTGCTTCTCCACT

>NF11217_low_28 signature: No signature
CTGAAAGTAAAAATTTGATTATTTCCTCTTTCTCACGTTCTCTACCATATATGATAGATTCATCCATGAA
TGATGAAGTCGCAAAATCATTGGCTCCATTGTCAAAATAACTAGTTAATTCAACAAGTTTCAATCTAGAT
TTTTCCTTTGCAAGAAACTCTAGCCTCCTGACCAATACTTTAATCCTAGATTGAAATCGATTGATAGAAG
CTGAAAGAAAGCATTGTATCTTTCCCTTTGCTTCTCCTCT

>NF11217_low_29 signature: No signature
TAACACACACTAAGTTTGATGAAATCATGGTGGATTCTGGGATTCTTTTGAAGCTCGAAGTGTAATTTTT
GCTAAATTTGCGGCGACACATTGCACTAAGTTTGACGAAATCAATGTGGTAATTGAGATTTTGTTGAAGT
TTCAAAGTGCAGTTTTTTGCTAAAATCACGGTGGCACAACTTAAATATTCCAAACACACAACTCCAATGT
TTGGTATTCCTTTCTCACGTCCCTATGCTACTCCACT

>NF11217_low_3 signature: ATGAGATGGACATCATCA 5' end
ATGAGATGGACATCATCAAATCCCTCAACACTTGGCCAATTATTCTCCGTGTATATTTTCCATACTCCTT
CCTCATTCCCCAACTTTTCTGAAAAATGTTCTCTTAGGCCTATAAAAACTACCTTTTGATCAATCAGATC
CATATTCTCAGTTCACTAACCAAAACGCCATTCTTCTTTTGTTCTCTCCTCTCAAATTAAGTGCAATGGC
AACAAGAAAAAGCAAAGCCATAGGCATTGACCTTGGTACAAGCTACAGCTGCATTGCAGTGTGGCGAAAC
AATCGTGTTGAGATCATTCCAAACGACCAAGGGAACCGTGTCACCCCTTCTTACGTTGCCTTCACCGACA
CTGAAAGATTAATAGGCGATGCTGCCAAAAACCAATTAGCCAAAAATCCCCACAACACTGTTTTTGATGC
CAAACGTCTGATTGGCCGTCGATTCTCTGACCAAACAGTCCAACAAGACATAAAGCTTTGGCCTTTTAAA
GTTGTTCCGAACCACAAAGACAAACCCATGATTGTTGTCAATTACAAAGGCCAAGAGAAACATTTTTCAC
CTCAAGAGATATCTTCCATGGTGTTGTCTAAGTTAAAGGAAGTTGCTGAAACTTATTTGGGTCATGAAGT
GAACAATGCTGTTATCTCTGCTCCTCCTCA

>NF11217_low_30 signature: No signature
TAACTGATTAAATAATATGCAATTCATTCGCACTTCTTAGTCTATAAGGTCTAACTCAATCAATAAAATT
CTCAAAATTGTTATACCGAATATTGTGACTGCAGTTTAAAACCTCAGTTACTCCACTTATGTGTGTGAGT
TTTCGATGGATATTGTCATGTTGTCTATCTACCCAAAAAAAAAAAAATTCGCACTTTAGACCGTGTAAAA
ATATTTGCGTTAGAGAATGATGTCCATCTCATTGC

>NF11217_low_31 signature: TGATGATGTCCATCTC 3' end
AGGGTTTGTATTGGTAAAGAAATGGCTTTGATGGAACTTAAAAGTGTGGCAATTTCTTTGCTTAGAAAGT
TTCATATTGAATTGGAAATGGAAGATAGAACAATGTTTCATGGTAACCCAAGATTCTCTCCTGGACTAAC
TGCCACTTTTGCTTTTGGTCTTCCTGTATTTGTTCGTCCAAGAGGAACCAAGTGAATAATCCGTACTACT
ATATATATGTATCTGATGATGTCCATCTC

>NF11217_low_32 signature: No signature
AGAGAAGCTCAAACGACTATGGTAGAAGATCTGAAGAAAGGTCAACCATAAGTCAACATATTGAATCAGA

AGAAAATGACGTCTCTTAGAAGCTGAAGATTTGCTTCATTAGACTATGTGGAGATCATCAGACTCAGACG
ACCTAGCAGACGCAGAAGACCCCATCAAACTTGGAAGACCTTCAGACTTTAGAAGCACTCGAGGGACCCA
AACTCCATGTCAAATTTGTCGTC

>NF11217_low_33 signature: AATGAGATGGACATCATCA 5' end
AATGAGATGGACATCATCAGATCTGCCATGTCCTTTCGCCGTGTTTATTCATTTTTGCCGTCAGCTCAAC
TGCTACTGTGATTTTCTTGATTGATACTCATACATTCTTTATCAAAGTAGAGTCATTTAAAATCTTATTA
TCTGCATATGATTTGTATTTTTATCTACATGTATTAACACTTGAGTTTCCCCATAAAAACAATCTCTTCT
GTGCTCCTCCTCAAGATC

>NF11217_low_34 signature: TGATGATGTCCATCTC 3' end
AGGTATGAGACTGCATGGTTGAAGGAAGGCTTATAAGAATTGTTTGGTACTACCTATATCAACATGATGC
ATCTTCTTTTCGGTAGCACATTTCATAAGAACTCCACAATTACGCGTGCTTGACTTGGAGAAATTTTGGG
ATGTGTGACCTACCGAGAAGTTTCCCGGTAAGCATGCGTGTGAAAAGACTCGTGTTGGTTTATAGGTGAT
GATGTCCATCTC

>NF11217_low_35 signature: TGAGATGGACATCATCA 5' end
TGAGATGGACATCATCAAAATGTAGTTGGCATTTGGCAATTTTTTAAGGGTTTCCAACTGACTCAACCTT
CATTTTTAACACATTTCTCTTTTTTTACTCCTAAAGAAATTCTAGCATCTCTAGCAATTTTCCTTTCCTA
GTTTCAAATGGACTGGTAAAAGCTGAGAAGCTTACTACCTTGCTAGTAGTACATTCAACTTCTCTGCTCC
TCCTCAAGA

>NF11217_low_36 signature: TGATGATGTCCATCTCATT 3' end
CGATCTTGAGGAGGAGCACAGAAGGAACAACAATTCATGAAAAAGAAAATCAATTTCATGGACAAAAGAT
AATGAAGAAAAACACCCAAATTCAAATTTTACAAAGAATCAATTCAAGAAAAGATGTTAGGGGAACTTTC
GTCTTTTTGACCAAAATTTATGTCATTCTCTCGCTTTTTTCAGTGATGATGTCCATCTCATT

>NF11217_low_37 signature: TGATGATGTCCATCTC 3' end
ATCTAGTGGAGTAGCAAAGGTAGTATAAATACAAACATGTTTTCTCATTCCATATTCATTTTCCTTTTCC
ACAAAGAAACATCAATACAACAAGTTTTTTTCCATTCATCATTCTTTAATTCTTTCATTATATTCTATTC
TACACTTCATTTAGTTTATTGTTTGGGTTCAATTCATAATAACATTTGATGATGTCCATCTC

>NF11217_low_4 signature: TGATGATGTCCATCTCA 3' end
AACCTAGAGTTTCTTGCTGATCAAAAGGATAGATTGGCACTTAACAAATTTACTAGTGGTGAATGTGAAA
TTGGAGTCTTGAAATTGTTAAGAGAATTTAGAGCTGTATCGAAAAGTTGTAATGACATATTTGTTGGTAA
AGATGGTAGAGTCATCCCCCGGATATTACCAACTGCACCTTTGATGGATAAATCTGCGGCGTATGGTAGG
GAGCATGAGATAGAGGAAATGACTGAATTTTTACTTTCAGACAGTTACAACCGGCTATCTGTAATCAGTA
TAGTGGGTCTGAAGGGGATGGGTAAGACAGCCCTTGCCCAGCTTGTTTACAATGACCACAGGATACAGGA
GCAGTTTGAATTTAGATCCTGGGTCCATGTTTCTGAATCTTTTGACTGTCTTCGTCTCATGAAAGAAATA
CGCGATCTTCAACCGCAACATTGGCAAGCAGGGAGTGAATATTTACTTGTTCTGGATGATGTCTGTATTA
AAAATAGTTATTATATGTCGGAGTATTTACTACCTCTTTGTAACCAGGGATCTTTTCGAGGCAAGATAAT
TGTGACGACAAATGATAAAGCAGTAGCATCAGTCATGAGATCGACCCAGATAATTTATTTAAGGCAATTG
GAGAAGAGTTGATGATGTCCATCTCA

>NF11217_low_5 signature: TGATGATGTCCATCTCAT 3' end
TCTTGAGGAGGAGCAGAGAATATGTGGGATGACATGTAGAAACCCAACAAAGACCCTACTGATGCTAATA
GTTGATCGTTCCTGTGTGTGTGGAAACGCGATTCACTTTGGTTGTAATTGCTAATTAACCATTTGTAACG
AAGGGAACGTGATTTGTTTGTTATTATTATGTATTTTTATTAAATAAATATTTGTTTTTTAATTTAAAAT
CTGCCACATGTCTTACATCCAAACTCTAAATGGAGTAAATAGAGTTTAGCCTAAATGGAGCATGCCCGAC
TCCAATACGCCAACCCAAAAATAGTCATAGCTGAACAACAGGAGAAAGAAATAAAGTAGAATCAACCGGA
GTACAATACGTTCAATCACATATGGTTCCTAACCCAACCACACCCTATACCCTCCATGGATACAATCACC
AAAAAAATTATACAAACAATGTAGATTTTGATGACCTATTTTCAAATTTAATTCCCAATTTTTTTAAAAT
GATTGAACTTTTCCCCATACATTTTGAGGCTGAATTTTAGGGAAGCTTTAAGTTATACCGTATGAAGGAA
GGAACTTGATGATGTCCATCTCAT

>NF11217_low_6 signature: TGAGATGGACATCATCA 5' end
TGAGATGGACATCATCATGTAACTCACCATGACAGAGCTAGTGTGTGGCATATTAATTTAGTGACCATGT

TTTCTGTCTCTAAAAAATATTTCTTCTTTCACTAATCTCCTACAGTGAGGTAAATATAATTTCCTCTTGG
ACCAAGCATAATCCCCTTATGTTAAATTTTTTCTTATAACAAAAAAATTAAATCAAAGAATGGCAAAGTG
GAAGATGAAAGGAAAAAAGCAGGAAAGAACAAGGGAACAGTTAGCTACTATACATTTATGTTAAAGTGGT
GAGCTTTCTTCCTGCTGTTCCCTAGTTATGTAGGCTTCAATATGAAAAATATGATGTGTCTCTCTCAACT
CAACCATCTAGAATCTAGCTTTGTCCAGTGCTATGAGTACTTTGCTGGATTGAGATTTAAATGTAGTGGA
CCATTAGTTTAATTCTTCCCCTATGTCTAACTCCAAGCTTATTCATATACGGGTAGCATTTTGGTGATAT
AGAATTGGTGATATTGGTGACATTTGAGGTTGGCCTGTGATTGGTTTGGGATCTTGGAGTTTGCTCCTC

>NF11217_low_7 signature: No signature
TCATCAACTGCTAACTATGCACAAGTTGAACATGGAGTTCCTCGAAAAGCAATAGAACCAAGTTACAAAA
TAATAACAAGAAAACCCTACAATACGATATTGATGTATAGTGAAGTCCTTAATATTATTGAGATTACATC
TCTTTGAATACAATTGTACATAAGTAGTTTACAAATATTGATGTATAGTGATGTACCTTCAAGATGACAA
CCTCTTTATATTATAGGTTCGATTCCTTTAAAGTAAATAGGTCTTCTGGTTGAGACTAATTCTAAGAGAA
TGTCTACTTGGAAACCTTTGTTGGATAATCAGGTAACACTTTTAAGCTCGTCGAGTAATAGATTGCCAGT
TTGGGTGGAAGTGTGATTTTTTTTAAAGGGACCTTTGTGGAATGATATCTAGTTGAGTGTTACTCCTTTG
AGGATTTGTTACCCCATGCTCTTTTAGGTCTCTATTCATTAAAAATAAAGTGATTGGTATGACTTTGAGA
GAGGGATATGGGTGGGTTTGGGATCTTAAGTGGAATCGC

>NF11217_low_8 signature: No signature
TCAATGAGATGGACATCATCAGCGTCGACATTGCAATCCACTTCACTGGCTACCAGTCTAATCCACCCTT
CTTGACTATGATCTGTCATTTCCAATGTTGTGGTATTCTCCTGGTTCTCTCCATTCTCAGACATACGCGG
ACCTACTGCCACTGAAGTAAGACATCACGCACCATACACACTGAGATACATGGTTGTGTTTCGACGTCAT
TCCACAACTTGTCGTTTTGTCGTTTCCAGATGCATCAAAGGGTCAGTACGAAATCAAAGAGTTGTTGGTG
TTGTAGAGATTCTACTAACTGAAAGTAAAGAGCTACAAAACCATATGTCGTATCCAAGTTGTCTTGAATA
ATGTTCTATAGACCTGCTACACTTCACACCTCCTCTAGTTTTGCACATCCGAAAAAGACATGTCATTCAT
TCTCATATGAGCTTTCACAAGGCACACAACAATCACAACAATGTACCCCGTGAGTTTAAAGCCCGTGTCT
CGTGGGTAAATACCTCTTCTCTGCTCCTCCTCA

>NF11217_low_9 signature: TGATGATGTCCATCTCA 3' end
TGAGCTTGTTGGTTCTAAATATTGTGTGTTATGTAGGTAAACAGTTTGTAAGCACCACATGGTCTTCTCT
TTAAGGTAATAGACGTATTCTAGATATTCTGCTTTAAGTGATCAACAAATCCTAAGCACCAAACAATTAT
TCGTTTATTCTTGACTTCATTTTGATAGTATATAATATGCACACTTTTGAAAACGTTAGAGGATAAAATA
TCTTTTTGTTATCATCAAAATTCGAAGATAACCCATATTTTGAATCTAACCATATTAACATGGTATACAT
TCAATCTAACATTCACATTCTTGTTAGATGAAACTCATATGGGTTACTCCACTCTATGTAGGCACCATTT
AAAAAGTGTGAGATTTAAATTAATTTTATCCAATAAAAAAGTGTTAGATATCGTATTTAAAAGAGAGTGT
TCATAACACTTCTCATTTTTATGATAATGATATATCTGACGGAAACCTTGCTCACATGAGCATCACACCT
GATGATGTCCATCTCA

II. TAIL-PCR FSTs obtained from a single Nod+Fix- NF11217 *Tnt1* mutant in R_1 generation

>NF11217-F-Insertion-1
CCCAACAGTATGCTGATGTAAATTTTTTTATTTTTTATTTTTTTTCTATAAATGCAAATTTTGATGATAAGTTAGTT
TCATAAATGCAAATGATATGTGGACTAGTCTTTTTTTATTTTATAAATTGGCTAGTATTTTTTATTCTTACTAGATAC
GGTATTAGCATTTTTACACACTACAATCAATATAATATTGAGCAGGGGGTAAAAATAGTAGTCAACACAAACCCGTT
TATAGAACTGATCAAAATACTGGTTCATACTAATCAGTGCAGTTTTCTAATGTAAGCTGATTTTGTACACTCGAAGT
TTACTCATGCCTACAGTTAAGTTAAAGGAATTGATGATTTTGAAAACTAGTTATGTGCAAATTCAGATAGACTTGTG
ATGACAACATTGTAACCATAATCTTATAACATGAATGTCAAGCGAAGTGCTATCAATACATTCTTTAACACTAAGAT
TGATTGAATTTCATGTAAGCCCCTCCATTTTGGAAATGACTTCCAAGCACTTAGGAAATAACTCCCAATAACTAGTG
GGACAATCAATGTTGAAAACAGAATATTAGAATGTGTTACAAGTGCTTCTTGATATTTAATATACATCCTTGGGTGG
GATTCAGATTATATGCAGTAAGAAAATCCATGCAATAACACAATTTACAAGTCTGGACTGTTCAATCTTGATTGCAC
ATTTCAATTAACAATCTGAGCTGTGCATACTTGCTATGTATTCTGAGCCGTCACATTGTCCGACTTTCCGACTGCAG
GATCAAAGTTTGAGATGTGCAATCAAGATTGGATTTGCCCTGAAATCGTAACTAAGTTTCCAACTTGCTAACTGTGT
GACTGCATAAAATCACATTTCCTTTACCGCATAAAAATCTGGAACACCTTTGGAGAGAGTTGCATTAGACTGAGAAG
CAGAATAAAAACTATCTTATTTTGGTATGCATCTTAGAAAATTATCTCTGCACATATATGTTATAGTAGAGTGAATT
GGTTGCTAGAGAACAAACGAAGTAGTTATTCACAGGCTA

>NF11217-F-Insertion-2
CCCAACACCTCTAACACTCACCCTCTTATTACGTGAAATTATGTTGGTTTAACTATTTTGAAAATTGGCCTCACATA
AGAACATTTCTACATGAATTTATCCTATTTCTGTTGCATGATGAATCAATCTTAATTTTATCATAGGTTGGAGGGTA
AGGTTGCTATAGTGACTGGGGGAGCAAGCGGAATAGGGGCGGAGACAGTGAAGACATTCGTGGAAAATGGAGCATTT
GTAGTTATAGCAGATATCAATGATGAATTTGGCCAGCAAGTTGTAACTTCAATTGGGTTAGACAAAGTGAGTTACCA
CCATTGTGATGTGAGAGATGAAAAACAAGTTGAAGAAACAGTAGCTTTCGCATTGGAAAAATATGGAACCCTAGACA
TTATGTTTAGCAACGCTGGAATTGAAGGTGGTATGTCTAGCAGCATACTAGAATTTGATCTGAATGAATTTGACAAC
ACCATGGCCATTAATGTTCGTGGATCACTCGCAGCTATCAAGCACGCTGCACGTTTCATGGTGGAGAGAAAAATCCG
TGGATCCATAATTTGTACTGCCAGTGTAGCTGCCTCTGTTGCTGGTTATCGTGGCTATGATTATGTTTCGTCCAAAC
ATGGTCTCTTAGGACTTGTACGTTCAACGTGCGGTGAGCTTGGAGCTTACGGAATTAGAGTCAATTCAATCTCACCG
AATGGTGTTGCCACGCCTCTTGCATGTAGAGCACTGAATATGGAGATGAGTGAAGTTGAAGCTAATATGAAAGATTC
TGCTAATTTGAAAGGAATTACACTAAAGGCTACGCATATTGCAGAAGCTGCTCTCTTTCTTGCATCGGAAGAATCGG
CTTATATTAGTGGACACAATTTGGGTGGTTCGATGGAGGCTCTCTGTTATAATAGGTTGTGTTCCTACTACTATTAA
AAAAAATAAGCGGTTCCAAATTTGGGAATGTGATAAAATATCTACATTCTGTTTCTAAGGTTGTGTAATTGGGAATG
CGTCTTACTATTAGAAAGGAAAGGC

>NF11217-F-Insertion-3
CCCAACACTTTGCGCTCTTGTCAACTTTGTTCTTGTTAGGCCCGAGCTCATCATAAGAAGTGATGGGGAAAACTATC
TTTAAAGGTGACATGACATGATTATGCACATACTGTAGAGTTACATTAGGATCCTTGGAGGCATTTCTACCAGTTTC
ATAGATAGTAACCTACTCCTCTTCAGACATGATGGTATGCTCCTTATGACATAACAAAGCCTCCCCTTTAGAAAGTT
CACACTCAGTGTCAAGCATATGAAATAAGTTATGAAGAGTCAAAGAAGGATCTGCCTCCATATCCTTGTTGATTACT
GGTCCAACAGTTTCATCAACATTGACTCCTTCCTTCATCAAATTTCTATCCACAATTGAAACAACCTCATTATTAAA
AACTTTCTCTGATGCAAAATTATAACTAGCTCAGTGACCTGTTCCTCACCACAATGTGTGGAGGGCATGGCAGGTTG
TTTTTCAGTCAAGCTAACATGTGCTTTGTTTTTAGTTTTCTGAATTTCAGCATTACCTTTCTTAGATATAGTCTCAA
AACCTTCCTTCTGATTAGTATAACCAAGGTTTTACAACTCGAGAGTTTACCTCAACTCGTTTTAGCTATGTAAACTC
GAATCGTAAACTCGTACGAGTTTACCTGAAATTAAATTATAGAAAATTTATTATAACATATGTAAGCTAATATTTAA
ACAAAACATAACCAATTAACCACATTTAAATATTAAACTCTAACAATAAAAAACAAAATTTATCAGTACATTTAAAC
TCTGTTTCATCTTCAGTTGTAACTTTAACTTCAATTGTCCCATTTTATCATTGAAATACTTGAGCAAGTAATATATT
TGAATGTTTTCAAATCAATCAATAATAACAAGCTTCTGAACGCATTTCCCTAAAATTTCATGTGTATGTCATTGTCA
CCGCAATGGGGTAGCTTTCTGTACATAACAAGCATGGTAAAAAACGAGGCCATGTTACCGGTAAGGCAACATTCAGC
ACTTGGAATGGAAAAAGAAT

>NF11217-F-Insertion-4
CCCAACACTTCACTTTGTATTTACCATCCTTTAACTCCTTATAAGGTTTGTCAGAGTATTCCTCAATCTCAGACTCA
CTGATATCCGACTCTTCTTCAGAGCTGTAGTCCATTGAAGCTCTGCATACAATTACAAATGTCAGTTCATAAGTATA
AAGTAGCATATAATTTGATGAGGAATATCAATCAACAATGAGCAATAGCATGTAATTTAAATGAAGAAAAGAACATT
AATCAACAATATACAGTTGCATATAATTGAAAGGAAAAAACTAATAATAACGGAACAAAGGTTAAATTTTAGAATAA
CTCAGATGGGATATTTTTGTTTGGTAGTAAAGGGTGGATGGAATATGCTAGTTAATTTTTAAAAAATAGATATCACA
TTCCCATCTATAACACCAATCCACATACCCTAAAAAGGTCACTTTCACAGAATATGAATAGGTGAAAATAAATAAA
GTTGGTTGAAAAAGAAAACAGAACTTGTGATCGACAGTTTGATTTCTGGAATGGTGTGATTCTAAACAACAAATTTT
AAATTGCGGAGGAATTTGTCTCAAACATCTGCAGATGCCCGATAGAGAACAAATTCAAAGAAGCACACCAAATACAC
GGGTGACGGGTATATAGAAGCATCTCTGGGTAAGAGCATAAAATATGTTTTTCATAAAAGAAAACAACATTACAGTG
AGATAGATGGACATGAGAGATATATATGATGCAATTAGAAAAAAGAATAAGATATTCCCCAAAGCGAAACAAACTGC
ATAAGTGCATAACATGCATTGTGCACAGTTTAGAAATACAAATTCAGAACATATAAAGGAAAACTACAGCCATGCAT
GAAGCAGAGTTACATCAAAATGCACTACTTATTTAAAAATATAGATTCAATAACATGCACCTCCTCCCAAAAGAAAA
TACCACACACAACTTAAATACTAAAAATTGTTAGAAAAACAGTCTGCTAG

>NF11217-F-Insertion-5
CCCAACAGAAACCACAGTGTTCTTTGACACTATTGTGAAAGCTAACCTCAGTTTCAGACAACTTCAAGTTTTCGAGG
GTATGTCTCGTGAAGAGCTTTTCTTGTGGTTACCTGTTAAAGATATCTTTGTTGTGGATCCATCTTCTGGTGTAATT
CTCATTGATATTGGTTTTGCTTTGAAGTTTCTCGCCTTCTCTCGTTTTGATGAGCCACCAATTTGTAGATCTCATCT
TGGTACCGTCTTTTCTCTCTCTTTTCTAACCCCTCAGTTATGGGTTTTAAATTTATTTAAGTAAATAATAAAAAAAA
TAAACAGGGTTTTGTTATAACATAGTTCTGATTTTTGAAATTGAATGTTTGATTACAGGTCTTTCATTTCGTATGGG
TGGAAGTAAAGGCACTGCATTGGAAGATCAAAGATAATAAGATTATTCATGATCTGTATGAAAGGATGGCTTCACCC
TTTTTAAATTCTGGGGGTTTTGTTGTTCAGAAAAATTACATTCATGTACAAAAATTTGCAACTCATATAAATTATAA
AGTATAATAATGCCATCAATAGTTGAATGATAATCAATACTAATATCTACCTTCAATTGTCACCTAAAAAAAGAAAA

AGTATCTACGAAGAAATTACCCCTAATTTCTTAAGAAGGATTTCGATTTTTGAGTAAGGATCCATTTTTGTCATCCA
ATAATTAAATGATAATCAATACTAGTATCTAGCTCAATTGTAACCTAATGAATAAGTTCTTCCTTCCATTCAAGAGT
AGAAACTACACCTAAGTACTTAGAAAGGATTTCGATAATTGAGTAAAGGTTCCATTTTAATGCTCGATAGTGTTTGT
CACATACTTAGTAACATTAAATTTTGTAATTTTTTTTCTCTCTCAAGTTAGGAA

>NF11217-F-Insertion-6
CCCAACAGCAATTACGAATATGTGCGGCAATTTCTAGTGGCAATGTCGAGGAGAACTTAGAAATGAGAATTTATGCT
TTGCCTTTTTTTTATGGGAAGCAACTTGTGTAAGAACATGCCCTTATAAATAACTTATGATCATAAAGATATTGCTT
GTCTCCATGTCCATGAGTGCGCTTTCATCTCCCACAAAAAATGTAACCAGCAGCAGCATATGTATTTTTTTTTTTTT
TTGAACAAGTATATGTATTTGTTTTTTTTTTTTTGACAAAAATATATGATATTCGTTCATTCAAATTGATATAGTGC
ATCGATACAATACAAATCCAAATTCACTAAAAACAAAAATGATGAATCTGTAAACAAACTCACAGCATCCATGTTAA
TAGCATAAAACGGCAAAGTATATGCTTACAAATATGAATATATTCATGTCTCCAAAATACTCATGTGTCCAGATCTG
CAACGTTGACGACTCCAAAGTCATTGTCATTGATCGGATCTACAGTTGCTCAAAGCTAATCTTTCAACCTGAATCAA
ATAAACACCGCACTAAGACGGGAAATCAAAATCACACTGCACAAAGACGGGAAATCAAAACATACAGAGTCGTTCAA
AGACGACAAAATCACGAAAACAAACGAAAGAAAATAGAAAATATGTGAAATCACTTATTCAATTAAGATAGAAAGAA
AAACAATTTGGAGGGGTGATTTTTGGGTCAAAATTGATCCTAAATCATCCATCTGAAAAGAAGAAAAAGAAGAAAGG
GGTGACTGCTAGGATTTGGGAAGGAGAGAAGAGAAGTCCTAAAAGGCTTGACAACTCCATATGTATTTTGTTTTAAA
GTGTCATAGATACTATATTTTTTAACTCTCTTTAGCATATGTATA

>NF11217-F-Insertion-7
CCCAACAGTGGTTGCACATTGTTTTGATTAAGAAAAATGTATAAGATTTGATAAATAAAAAATGTATGTTGGAGAGC
GCAAGGGAGCTGCAGCCGAGGATCCGTATTGATCCACTGGTTAGAAAAAACATAACCAATAGTTATACAATTTCTTT
TTAACCAAGTGATTGAGATAAAGTGGTTAATGAACTTCACCAAGAATGAATTGTTTCGGAGCAGTGACGGAGCCCTT
CATGAGCTGGGGTGGGCCACGGTCCACCCGGAAAAATTAAAAAATCAACGATTATAGATATATTTTAAGATATTTTA
TGCAATTTTGTGTCGGTTTTATGTTTTTGTTGATCCAAATTCCTGCAAAGTGGTGTAGTGGCCCAACCGAAAATTTA
AATAATTCAATTATAGGTCTAATTTGCGAGATCTTATACAATTTTATGACGGTTTTAGGTTTCGGTTGATCCAAATT
CCCGCAAATTGGTGTAGTGGCTCACCCGAAAAAGTTTAAATAATTCAATTATAGGTCTAATTTGTGAAACTTTAAAC
AATTTTTCAATAGTTAATTTTAGTGGCCCACCCGAACA

>NF11217-F-Insertion-8
CCCAACAGTCGGAAACCAAACATCTCTCGTTGTTTTCCTTTTCATGTTGTTGTTTTTCTGTTGCATCACATAGCACC
GTGTTGTTGAATGCTTTCCTGAAAATGCCAACACAAACATCCGTAATGTGAACGGATTTAATTCATATACAATGTCA
AGATAGATATTTTTTTACGTTGTCGAAGAGACTTGTAACCACGAGCTAACCAGATTTGTACATTTATTCCGGGCTCA
TTGTACAGAACATGTCATATAATGTGGCTCAGAAGAGTTTATATGATGGTGTTAGTTTTATAAAGAACGGTCTATAT
AACTATATGTTGAGAATGTAATGTATCAAAATTAAACTCAATGTGAAAATAAAAATAATAATAATAGAAAATGTGAA
TTACCAAAAAGATACTCTGGCATTGGATCCTTCGCATTCGATGTAACGATTTACAATAATGTCACCCAAAGAATGTG
TTTGTTGTTGTTGTGATGGTTGAAATTTATGCTGGTGTGCCTCATGTGATTGATAAAAATGTTGATATTCTTCGACA
TCCGAATCACTAGTGAATTCGCGGCCGCCTGCAGGTCGACCATATGGGAGAGCTCCCAACGCGTTGGATGCATAGCT
TGAGTATTCTATAGTGTCACCTAAATAGCTTGGCGTAATCATGGTCATAGCTGTTTCCTGTGTGAAATTGTTATCCG
CTCACAATTCCACACAACATACGAGCCGGAAGCATAAAGTGTAAAGCCTGGGGTGCCTAATGAGTGAGCTAACTCAC
ATTAATTGCGTTGCGCTCACTGCCCGCTTTCCAGTCGGGAAACCTGTCGT

>NF11217-F-Insertion-9
CCCAACAATCTGTTCATCACTAAACCTTCTCTTGTTTTCTATCTTCTTGTTTTTCTTCTTTGAATTCTGAGGTGTCT
CCAAGCAACTCATGCTTTCAACATTTTTTCCTTTAAACCCTTCATGAATATTCTCTTCTTCCTCCATCATATTATAT
ATGGCTAAGGCCTAAGTTTTGAATTTTCGGAATTTGAAGGAAGATTTGTTTAATAATAAAAAAGATTAAAATTTGGT
GAGGGGGAATAGGATGTAGAATATATATAGCTAAAAAAGAATGTATGTCAAGGTTATTTGTATCACGTTATTGGAAC
TTGTAAAGGAAGGATCATAGTGACTTATGGTACTAGAAAACGTGTGGTGCTTACTATCTTTTTATTTGCTAGGTAGA
AAAAACGTAGACCGTTACTTTTCTCACTCTACGAGTTTCTCATGCGTCCTATTCTAATTTTAACATTCAAAATATTT
CAAACGCGTTTTAAGAATTTTTTATAGGCTGTCTGTATTGTTCGGATTATATAACCTAAACTGCATAAAACACACGA
GAAGCTCATAGAGTTGGAAAAGAAGCACTCAAGAAATTATCTTTGTTAGTTTACTTGTTGTAGGACATCTAATGGCC
CTATCTATTTCATTTGTAGAAGGAACTCTTTACTTGTCACGAGTTGTCACGTTCATGCCACACG

>NF11217-F-Insertion-10
CCCAACAGTCTTTTTCATTGATAATGCAGGGGTAATGGCCTTTTCCGGTTTCTTTTCTTCTACAGCTTGGTCATCTT
TAGATCTAAAAGAACCTAAACATGATGCAGACAATGTTTAATAAACTTGATTTTGTTTTTGTTTTTTTACTTAAAAA
TGGTAATTATGATTCATAAATGGAGTGGTATGTGCTCATCTTTACCTGATAATCGCTTGGATATACTACTGAGAAGG

GACTTTGAAGATTTTGAGCTGTCTACCTTGCTTTTGACCTTGGTCAATGAGGCCTCCGGGGTCTCACTCTTCTGCTC
AGAGGTCTCTTGCAAATTAATTGAGAATGCAGCAGCAGCTACTGCTGCACCATGCACCATATCTATGTAGTCGCAAT
CACGACCTGTTTTACTTGAAAGCTGTTTTTGTAACCAACTCTGGCCCTTTTTCTTCTCTGCTGATAATGGTCAAGAC
AGTTTTTACACAAGGTCAAAATGAAATTTTATCCCTCGTTTTTCTGAACTTACTAGGCTGCTGATTGCATGCTCTTA
TGTGTAATTGTCTAGACTTCATTTCTAAAAACTCTTCAAATAAACCATGCATGGCATAAAGTTTTTTTTTTTTTTTT
TTTTTTATC

>NF11217-F-Insertion-11
CCCAACATGCACCTTGTTTGGAGCAACATCTTCAAGTGTTTCCTTCTCATTTCTATATATTCCTTTTTCCAAAACAG
ACTCTCACCCTAAACTTCTCTTCTTCTTCTGCAAAGTTCATGTCCATAAACTTGAAGTATAATGCTTTCGTCGGAAA
CCTTCTACGCCATTCACGGCTACCCCTTGTCACCCTTCCTAACTCAGTTAATTTGTACGATGACATTTAGTTTCGAC
GATGGTTTGTGTGAAAATTTGAATTTTAAGTGTGGAGAGATTTTATGGTTTTTAACATTAGTTTTTTTTATTTTTGA
AGGAAAGGTTTTTTAACATTAGTTTTTAAGGTTCTTATTGATTCTCTAATGATTTAATTAATTTACTGAGAGTTTGA
TTAAACTTGTTTTGAATCAAATATTCAATGAATTGAAATAATCTTTGTCTAAATTGCGTTGAAATTTGTTTGGTGAT
TAT

>NF11217-F-Insertion-12
CCCAACAACACATTGAGTCAACTTCTTCCTTTAAGGAGACAAGAGACAACAAGGTTTGTTAGTTTTTTGCTCAAGAA
AGGGAAGGAGAATGAGGTTATTGATGTTGGAAGAGAGCTTTTGAAGTTGTCGAATAACGTTATTTCGAGGATGATTA
TGAGCCAAACTTGTAGTGAAAATGATGGTGAAGCTGAAGAGGTGAGGAAGTTGGTGCAAGACACTGTACATCTTACG
GGGAAGTTTAATATTTCTGATTTTATTTGGTTTTTTAAGAACTGGGATGTCCAGGGGTTTAGCAAAGGGTTAAAGGA
AATTAGGGACAGGTTTGATTCTATGATGGAGAGGATTATCAAGGAGCATCAAGAAGTAAGGAGGAGGAGAAAGGAAG
TTGGTGGAGGAGAAGGTCAAATTAAGGATCTACTTGATATTTTATTGGAT

>NF11217-F-Insertion-13
CCCAACAAATTTTACTGTTTATCAAGGAATATATGTTTTAATTCTACTTCTTTATTGTTAATATTACTTGTTAATTT
TAGTGAACTCTGTATATTTCAGCAATGAAATTCAGTAATTGTATGTGTTTAATTCTAATTCTTTACACTTTTTATAG
TTATTTTATGAAAGTTAGCTAAATTGATTGGATTTAGCACAGAATTTTCCTCGGTCTTCTTTTCACTCAAGTGTTTT
GGATCAGTACTATGCCAATTGTTATATCTCAAGTTAGAGATTAATTTCTTTCTATTCAATCAAATTACACCAAAGTG
TCACTTTTATAGGGTAGTTTTTTTAAATATCTCCACAAATATC

>NF11217-F-Insertion-14
CCCAACAGAATCAACTTACTGGAAATCTTGGAGCAGATAATATGGCCACACAACTATCAAGGTGCATATTCTTTGTT
GGAATGGGAAGCAATGACTACCTAAACAACTACCTTATGCCTAATTATAACACCAAAAATCAGTACAATGGACAACA
GTATGCTGATCTCTTGGTTCAAACATATAACCACCAACTCACTGTAAGATCTTTTGTTTCTCTTACTCACTCTAAGT
ACCTAGAACTACATTTTTGTCAAAGACTGTATGAAACACGGTCACTCCTCGGATTAGGCGTGTTTCAGTGTCGGACA
CCGTGTCCGTGTTTGTA

>NF11217-F-Insertion-15
CCCAACACTATTCAAGAAAAATAAGTGTATGTTTGGATTCATTTTAGGATAGGCAAAAGTGATTATGAAAACTTAAA
ATAGATTTTAGTATGTTTGTGTTGTTTTTTTTATTGGAAATGGTTGTCATTCTAACTGATACTAGGATTTGTAGCTG
TAGCCTGCAAACTTTACTTTTCACCCCATATGAGTTCCTCGCATGTTTTATTAGGATTCTAAAATCCGAAATATTTC
AACA

>NF11217-F-Insertion-16
CCCAACACACTTTTCTATCTCCTGCACGCATTCCAAGGCAAATCTAGAAAGGCATTTTCCAACAACATTCTGAAGTA
TGTGCAAATGACACTACACATGGAGAAAGCCCAAAAATTCATCTGACACGCATTAAATAAACCCAGGAACACATCAT
ACATTCATGTTTGATGAACCTAGGTACAATGTCATGATGAACAGAACAACATTCTCCA

>NF11217-F-Insertion-17
CCCAACAAAAAGCTCCCTAAATAGTTTACAATTTGTAAGTTTTAGCATGTTAAATAATGAGCTTAATATAAAAGGTG
GATTTTCCATGCCAACTTGGTCCCTCAACTAGTTCAGAAACAAGAAGCCCACAACTTTGCCTAAACTTGTGAGCATG
ATCAACCCCACCTTATCCAATTGTTCATCTGTTGACAGTGTTCTGAATCTAA

>NF11217-F-Insertion-18

CCCAACAAAGGATTAGGGAAGTCTATAGTTGCTTAAATAGGAAGAGACAGTGTAGAAAGAGTTGAATATCAGAGTGC
CAAAATTGAAAGAGGATTAGTCAACCTTAATCAATCAGTGTGTATTGCGTATTTATAGATACGTTGCTGAATGTTGA
TGATGAGAAGGGTATTTTACCTTGTGTATGTCTGCGCGCGT

>NF11217-F-Insertion-19
CCCAACAGCACAGTATTCAACATCAGAAGAATATCTAGAAACAGTAATTTGTTTCTTGGTTCTCCATGAAATCAAAT
AATTGCTTAAAAAGAAGCATTGTCCAGTTATAGAGCGCCAAGTATCAATACATCCAGA

>NF11217-F-Insertion-20
CCCAACAGGATTGGCAATTTCCAAAGAACTTGGACTGAGGTTGAGGGATTTGGCAATTTCCCATATTCATTGATCCT
TGATCAACTCATTATTTCTACACATAGGAATTCATGTCAGCTTGAATGTCCTATTGACTTTCTTATAT

>NF11217-F-Insertion-21
CCCAACAGAACTCGCATGCGAAAGACAAGAACTCAAACGTGGCATTTGTGCAAAGAAATTAGAAACAATTTTATATC
TACTTGAGTGGTTAGCTAAGTTCCATAT

>NF11217-F-Insertion-22
CCCAACAGAGGATAAATTGCCATCAACCTGTAGAAATGAAAGGGAGTGAGATTGAATTTGAAATTAATGAAGATGAT
TAAGGAGAAGGAGAAGGGAGTGGGGGTT

>NF11217-F-Insertion-23
CCCAACAACTGTATGGTTTGTGTTATGTTGTGAGGTTATTTTTGCTTAATGCTACCTGATTGCTTTGCTTTGTCTA

>NF11217-R-Insertion-1
CATCATCATGTAACTCACCATGACAGAGCTAGTGTGTGGCATATTAATTTAGTGACCATGTTTTCTGTCTCTAAAAA
ATATTTCTTCTTTCACTAATCTCCTACAGTGAGGTAAATATAATTTCCTCTTGGACCAAGCATAATCCCCTTATGTT
AAATTTTTTCTTATAACAAAAAAATTAAATCAAAGAATGGCAAAGTGGAAGATGAAAGGAAAAAAGCAGGAAAGAAC
AAGGGAACAGTTAGCTACTATACATTTATGTTAAAGTGGTGAGCTTTCTTCCTGCTGTTCCCTAGTTATGTAGGCTT
CAATATGAAAAATATGATGTGTCTCTCTCAACTCAACCATCTAGAATCTAGCTTTGTCCAGTGCTATGAGTACTTTG
CTGGATTGAGATTTAAATGTAGTGGACCATTAGTTTAATTCTTCCCCTATGTCTAACTCCAAGCTTATTCATATACG
GGTAGCATTTTGGTGATATAGAATTGGTGATATTGGTGACATTTGAGGTTGGCCTGTGATTGGTTTGGGGATCTTGG
AGTTTGCTCCTCTCAATGTCTCAGGTTCAATTCTCTCTAGTGCTAATTTCGAGGGAAAAGTTGCTCTGGCTTAGAAC
AGAGCCCCTATGGACGGTGGAATTGGTCCCCTCAGATTAGTCAGTCCTTGGGATCGGACATCAAATTTTCCAAAAAA
AGAAAGAAAAAAAATAATAATTACCAATTACGGTTGACTAAAACTTAGAAGGAGGACAACTGTTTCTTGATCTCAAG
GGGGTAATATATAAATTTCAGTTAAAATTAAATTATTCTGGAAGTATCTGGAGTTTAGTCCCAACTCCAAAGAAACA
GGCTGGCCTTAAAGAATCGAATTTTCTTCTTGAGATCGAAAGGTTTTTCTTTTTTA

>NF11217-R-Insertion-2
CATCATCAGTTGATAGAGTGTTCATCGAGGCCACAGAAGAAATCAACTGCACATCACATGAAGACGAGGCCCAGAAA
ATCATCACTGTCAGATAGGAATCGGAAACCAACAGAGTATGCTCCGTTGCCACCACTGCCTCCTGATTTTACAATTG
TCATTTCTGCTGATGCATCTTCAACGGCTACTGTTGTTCCTCCACCCCCTACTCCTTCTACTTGATTCGGTACTTTT
TTTTATTTTATTTTATTTTTAAAGTATCTGTGTTGTTGTTGTTGTTTTATTTATTGGACTATGATTGTACTGTCAAA
ATATACTCGCTACTTGTTTCAGTTTTATCTTTCAATTCAATGTGATTTTCATGTTTACATATGATGATGACAAAATG
TCCACCATGCTATTATGTTATGGAAACGCGTTTTACAGCAACCCACATAATTATTGAAGCTCTTTCTTCAATTGTTT
TGACGTTAGGTATAAATTATAGGTTACCAATTTCCTTTTTGTCATTCCTTTCAAAATATCATGATGATATTCAGCAA
CGTGCATCATACTTTAGGTGCAATCGTTGGGAGTGCCAAAAAATTCGGTACAATAGAAGCTAATCAAGTTTGCTCAC
TATTTAGCAAAATATACATTTTTCTATTTCTTCTTATGATGCTTGGATTGAAGAAACACCTTGTATTGATGCAACCG
TGG

>NF11217-R-Insertion-3
CATCATCAGAATCTTTTTTATCTTGTAAAAAATTGTAAAGTGAATTCTCAAAAGTGTCTTTCATGAGAACATGTTCT
GGGTGAATAAAAGAATCGTTGAAAAATAAAACTTACAAAAATAAAAATATTTTATATTTACTTATAATGTAATAGAT
CAGAAGTAACTTTTAGGGTGTGTTTGGTTGTGATGAGAAGAAAGTAAAAAGAGAGAAATAGGTGAGAGAGAGTGTGG
AAAGAGAGAAAAAATGTGAGAAATAGAGTAAATTTGATGTATTATTTGGTATAAGAGAAAGAAGTGTGTCTGATTTTT
GAAATGACAAAAATATCTTTATACTATATATACACATATATGTGTATTTTTATTTTATTTTAATTAAATTAAGGTCA
AATAAATTCACATTCCACTAAATAAAAAAATTCACATTCCACTGTATAAAAAAGAAAATAATTTATTCCACAAAAAA
AGAATTACGTCAATTAAACGATTGCAAAGTAGTAATAATAATAATAACAACTAAGTGAAATACTATAATGTAATATA

TCCTTTTTTTATCCAAATATATATCCTATGAGATGAGAAAATAAAAATATCTTGCAAACCATCACAGGTTCTGCTCG
TTCACTGCC

>NF11217-R-Insertion-4
CATCATCATATATTTTAGATCGAATTTACAATATAAAATTCTTCTACTATGGAGAATTGGAAAATATTATAATCAAT
CTATAATATCACATAGATATATTAAAAAATATAACTGACCATTTCCATTGATCACCATTGAGACTTATATACTTCAT
CTATTATTTCATAATACAACTTGCAATGTCATGCATAATAGAATTTAGGCTTAAATAAGAAAAAGGTTCATGTAAGT
TCGCGCATTTTTTGTTTTAGTTATTGTATCTTTTTTTGTTGCGAAACAACCCCTGTATTTACTTAATATTTTGACAT
TCGTCCCTGCCGTCCATTTCCGTAAAAAAAAAACGCTTCTCTGGCTAACGGACTAATATGTGGCATATGATGAGCTG
ACAATTGGCTATTAGAGGGACTAAAACCAAAAATGCAAATTTACATAGGGACGAAAATAAAAAACATAATCATCAAA
ATTTGAATTTTAAAACATATTATTTCCACCTTCTTCCTCACTATGTTATTTCTCTCTTC

>NF11217-R-Insertion-5
CATCATCAATAGCCAGGGGCGGTTCTATGGCCTGGCGACCCCGGGCACTCGCCCGGGCTCCGGGCCAATTTTTTTGC
AATTTCTTGTGTAAATCCTTATGAATTTCTTACATAAACACCTATAAATTGGACAATTTCTTTACATTTTTTATTAT
TTGCTTGCAATTTCTTATGTACACCCTATAAATGTGTTGCAAATTTTTTTTTGCCCGGGCTTTACAATATTCCTGGC
TCCGCCACTGGCTATAGCTCTATACATGTCAGATTTCTTGTGGTTTTAACTCTGAATTGGAATGATCTTTACCATTT
GACAGTTACTGTGAATGAGGTTGAGGCTTTACTTGACTTGTTTAAGAAACTAAGTAGTTCAATTATTGATGATGGCT
GCATTCACAAGGTAAATTTATTTTAGTGCGAGTAGTACAAATCAACTAAAGACTGATTCCAAATATTTTCCTCTATA
TGAACTGATATTGCA

>NF11217-R-Insertion-6
CATCATCAACCACAAGATTTATGCGCAAATGGCTACTGCACTCCATCTAAAGAGACAAATTAATATCCGATTTCAAT
TCTTAACTATATTTTATTTAGCTTTTGCTCGAACAAAGATTAGTCTAGTGACCCCTTTTCTCAAAAAAAAAATTAGG
ACAATGTGCAATTGATGATGGGAAAATAATAAACGTACCAGAGCAAGTCTTGCTAACGGTGAGCTTGTATACAAGGA
CTATAAGGCCAATAATATGGATTGATCCAGAGGCTATGTAGAAAAAATGAGGGTCTGTGATAGTATGCTTTAGAATC
ACCACAGCACAAAAAGCAAGCAATATTCCCAACAAGATCTTCACTTTCATTGACCTCTTACTCAACCATCCAATCAA
AACATTCACTGCAAAATCCCTCTTTGATCCCA

>NF11217-R-Insertion-7
CATCATCACATAGCACCCCCACACACAAGAAATCTAGTGCGCATAATGTTGCGCCAAACATAGGTGGGGTTGTGCCC
AACAATGACCGTGAGATAGGAGCTAGACGGGAAATAGTGAGCTTTAAAAATTCGAGCTACCAAAGAGTCCCGATCAG
TGATATATTGTTGCAGTGCGTAGACACACTCCATGATTCATCTACACCACAGAGTAAAAAAGTCCACCTTCCTCGTT
ATTGCATCTAAATACTCTTGTTCCACTTAACCGTAAGACTTCTCAAAATCAAATTTATACAACAATAATTCTTCTTT
TGACTTTGATTGA

>NF11217-R-Insertion-8
CATCATCATCCTGCTGGTATAGGATTGACACATCACGTGTGTACGTCGTCTTATAATTGTGTAAAGCGAGAGGAAAT
ATTGTAATGAAATGTGAGTAATTTTTTTTTTTGGTGAAGTAGCTGTTAAATCACACATTTAAGAGGAGTAATAAATC
TAGGATTTGAATCTTAATCTCTACGTTAGTTCCTTTTTAAGGAATTTATGTTTATCAACTTAGTTACATTTAACAAG
ATAATAAGGGTAAATTTTTTTTTTTTCATTCTATTAGGCGCCCCACTCCGTGCTG

>NF11217-R-Insertion-9
CATCATCATGTTTGAGCCCATATCTCAATGTTAAAAGTAAGAACTTAAGGTGTTAGCAGCCAACACTCGGTGGATTT
AGTCCCACATCGGATAGATAGGATTCTTGAGAAGAGTTTATAAGGAGTATGAGTTAGGCCTGGATTTACGTGACATA
AGGAACCTACAAATTATATGTGTGTGTCCCCACAAGACAGAACACTATCCTATATATCTAAAGAACTTAGCAAACTT
TGCCGGAAACTTTACGCCAAGCTTTAATTGAG

>NF11217-R-Insertion-10
CATCATCATCTAATGTTTAAGATGTCATTGTTGCGAATAAATTTAGATTACGGGAGTGGGTTATAATTGGAGGAGAA
GATTATTTGTTTGTGAAGAAGAAAGTCTTTTGGAATGTATGCATTGCTTGTTAATGTGATTTTGCA

>NF11217-R-Insertion-11
CATCATCAACCCTCACCTGTGCCGGCCCCTGAAGCAGCACCGCCTTCAAATGCACCATGGGCTGCTAGCGTGCAGAC
ATCAGAGTTAACTTGGTCTCCGGTGCCCTCGCCCTCACCT

>NF11217-R-Insertion-12

CATCATCAACTCTAACTTCCACCTGGCCTTTTTCATCAAAATAAATATGTAAATAAATTGATCACTATCTCTCTCTT
CATTATTGAC

>NF11217-R-Insertion-13
CATCATCATGAATTCTTATGATTGTAGCTGATATTAAAAATTTTAAAAATCATTAAAGAAAACAAAACCACTTTACC
ACCCCA

>NF11217-R-Insertion-14
CATCATCAGATCTGCCATGTCCTTTCGCCGTGTTTATTCATTTTTGCCGTCAGCTCAACTGCTACTGTGA

>NF11217-R-Insertion-15
CATCATCATAATACTCGCGCCCAAGAGCTCAACCACCACCCTCTATCACGCTTCAT

>NF11217-R-Insertion-16
CATCATCAAAGACTTCATCATTCACTGGAAAAAAGACTGACACCA

III. TAIL-PCR FSTs obtained from two different Nod+Fix- NF11217 *Tnt1* mutant plants in BC_1F_2 generation

NF11217-6-Insertion-1

CATCATCAAAGAGTTGGTTAAATTATTCCAAAAAGGAATGATTGGTTTCAAACTTGCCATTTTTATTTGTATATGAT
ATGATCAGTTTCCCTCATTTACTCCACAAACGACAGTAACCTACAATTATCCATGGCAATGCAATCACCTTCCTCTT
CATTTTCCTATGGATTCACTTACCAAGTGTTCCTCAGTTTCAGAGGAAGTGACACTCGGTATGGTTTCACTGGAAAT
CTCTACAAGGCTCTTACTAACAAGGGAATCCACACCTTCATTGATGATAATCATCTTCCAAGAGGAAGCGAAATCAC
ACCATCACTCATCAAGGCCATTGAAGAATCTAGAATTTTTATTCCAATATTTTCTACCAACTATGCATCTTCTTCAT
TTTGTTTGGACGAACTTGTCCACATGTCATTCACTGCTACGAGACAAAGGGTCGCCTCGTTTTGCCTGTTTTTGTTG
GTGTGGATCCTACTGATGTGCGACATCATACAGGTAGTTATGGTGAAGCATTGGCTGACCATGAAAAAAGGTTTTCA
AAATGATAAGGATAACATGGGAGAGGTTGCAGCGATGGGAAAATGGGCTATGAGGCAAGTTGCTAACTTTGTCCGGC
TACCATTTTTAGTCCCTTGGGTATCCCACTTTTACTTAAATCATATTTTCCTTTTTTATATTTTCCTTTCTTTATTT
CAAATAAATTTCCCAATAATTCTCGCATGGTTGGCTTTAAATGTTGGTGGGTAAATTGGTAGTGTAATTTCCACTAA
CCTTATCCTAATGGGGGCATGTGATTAGGAAACCGAAATTACGAAGTTTTTAATTGGGGGAAGAATAGGCTCGCAGA
TAT

NF11217-6-Insertion-2

CATCATCAAAGACTTCATCATTCACTGGAAAAAAAAACTGACACCACAACACCAGAAGAGACTACAGGGAAACCTGAC
CCTCAAAGAGAGACTACAACAGGATCAAACAATGGGGAGACAAGTGCATATGTATTGGATGAAACTTTGATCGACAA
GATCAAACAATGGGCAAATATACAGGAGAGAACTGAGCCCGACAAGATCAAACAAAGGTACTTGGAAGCAGGATTAC
ATTGAATTTCTAACAAAGAAAACACTCTCAAACATAACTAGAATAAATATTATCATCTCTAGAATCAAGCATTACAT
GCAGTTTATATACTAATGAAAAACATTGTCAGCTGGTATGAGAAGTCTAAAGAGTTGATAGATTCCTGGGAAGACAA
GAAGAAGATAAAaGCCAAaCGCGAGCTAaTGAAGCAACAGGTTTCTCTCAGACACTCTTTATTAGGTTTCATAGATC
TAATTCATTTTTGCTTTCTTTAAGCAGGTCATGAAATGCTTCCTTACCTAAAATTAGATAATGTTCTAGGTCTTGTT
AATTACCC

NF11217-6-Insertion-3

CATCATCAGTACATACCTTACGTACAACTCAGTGCAGGGGATTTTCATACCATTGTTTCGTTGAGGAGATGAATCTG
AAGATGGGTTTGGTTGTGGAATTCCACCAGCAGCAGCACCAAAACCTGGTCCCATTTGACTACTACTCATGCTTGCA
AATAAACCACCAATAGGATAAGGGTACCCTTGTTGTTCTTCATGATGGTTATTCCATTGAGAACCTAAATTGGAGAT
TGAGAAAAAATTGTTTCTTTTGTCATCAAAGGGATTGTTTTTGTGATTCCTTTGAAACCCATTATCAAAAGTGTGAT
AGATTAAACCCTTTTGGTGTTTGAAAAGGTTGGCGGAAATGCAGAGGAAGGAAGAGATGAGGAGAAGAAAGGTTGTG
AGTGATTGTGTTTCTGTGAACACACATACACAACCATCTCGAGAGATGACAAGGTGGTTTATTG

NF11217-6-Insertion-4

CATCATCATGTTTGAGCGTCGTTTCTAACAGTGACATGTTTTCTGATGTTTGGATAATGAATGAATACGGGAATGAA
AATTCTTGGACTATATTGTTACGGGTTCCTCACATGAGAGATTTTGGTTTTATTGGCTATCAAAGGGTATTATATAT
TTCAGAGGATAACCAAGTGCTAaTGGAGTTTATATCTTCGGGGAAATTTAGTTTGGTTATCTATGATTCCATaAATA
ATACATTTAAGATTCTACAaTAATtCAAAaCAACAaCAaTGATAAAaTGGCaCCACCaCAAGtCTACGTTGAGAGTT
TGATATCaCCTCAaCTGCAGCCTAgAGACTCGTCTTTTTCTTCgATGaAATGATCTTGTTAATTACC

NF11217-6-Insertion-5

CATCATCACCTCCGTCCCTTAATAAATAACCTAGTTGATAATATGCATTATTGACTTGATATACTTTGACCATACTT
TTCTACTAATTCACAAAGATAAATTATATCATGTAAGATGTTGTTGGATTCGTCTCGATGAGTATTTTTAAAATATT
AAACTTTTATAATTTTTTTTAGTAGAGAATTGAAGATATCAAAGATAAAACATATGCTTGGTATGTGTGTCAGAGTC
AACTAGGTCTTTATTAGGGACAAGGGATATTGGGAAGGAAaTGGAACTGCCTTTGACATGATATGCTACAAAAATGA
CATCTTGACTCTGTAATAAATCGGAATATAATTGTTTTTGTATTACGATT

NF11217XR108 9a-Insertion-1

CATCATCACCTCCGTCCCTTAATAAATAACCTAGTTGATAATATGCATTATTGACTTGATATACTTTGACCATACTT
TTCTACTAATTCACAAAGATAAATTATATCATGTAAGATGTTGTTGGATTCGTCTCGATGAGTATTTTTAAAATATT
AAACTTTTATAATTTTTTTTAGTAGAGAATTGAAGATATCAAAGATAAAACATATGCTTGGTATGTGTGTCAGAGTC
AACTAGGTCTTTATTAGGGACAAGGGATATTGGGAAGGAAaTGGAACTGCCTTTGACATGATATGCTACAAAAATGA
CATCTTGACTCTGTAATAAATCGGAATATAATTGTTTTTGTATTACGATT

NF11217XR108 9a-Insertion-2

CATCATCAGAACATACAGGGAGAGACAGATGGAACCTGTACATAGTCCTAGTCCACTCCCACGACCACACTTGTATC
TCCTCCACCACTTCTTCAATAGACTCAACACCATCATTGAAAATTCTGAGTTAATCAAAGTCAGACTCTCTCTAATT
GACAATTGTCTACTTTTCCAACAAGTAGACAACATTTTCATCAACGACGACACAATAGGCTTCCAAGTATCAACTCT
CCTTAGATTGGCACCCATCAAAATACCCAACTTAATAGGAAATTGATCAATATAACAATGTAAAAAaTCCAAAGGGC
ACCATCAAAAAaTCATCGTTAATATTCAAaCCAAAAATATTACTTTCCCGAAaTCAaTGCAAAGAtTCTAGTAACAG
AtTTAAGACACCATAAATtCTCTCAaTTCCcTTCACAAaTAAAAAAaGtATCATCCGCAATCGTAAAaTAGgaAAAG
ATCGATGGAGTtATCATGAAACACCTCTTCAAAGAGCCGATTTCCACCATTTTTGCTACCAAACTCGTCAATATCCT
TCCTCTAATGAAATCTCACTACCAAGGTGTTTACAAAGTGTTTTCCAACCCAATAACGTTAGCCTCTAAAATTACTA
AAAGTTTCCTCCTTTGGTTTGACAAGATTAAACACACAA

NF11217XR108 9a-Insertion-3

CATCATCAGTACATACCTTACGTACAACTCAGTGCAGGGGATTTTCATACCATTGTTTCGTTGAGGAGATGAATCTG
AAGATGGGTTTGGTTGTGGAATTCCACCAGCAGCAGCACCAAAACCTGGTCCCATTTGACTACTACTCATGCTTGCA
AATAAACCACCAATAGGATAAGGGTACCCTTGTTGTTCTTCATGATGGTTATTCCATTGAGAACCTAAATTGGAGAT
TGAGAAAAAAATTGTTTCTTTTGTCATCAAAGGGATTGTTTTTGTGATTCCTTTGAAACCCATTATCAAAAGTGTGAT
AGATTAAACCCTTTTGGTGTTTGAAAAGGTTGGCGGAAATGCAGAGGAAGGAAGAGATGAGGAGAAGAAAGGTTGTG
AGTGATTGTGTTTCTGTGAACACACATACACAACCATCTCGAGAGATGACAAGGTGGTTTATTG

NF11217XR108 9a-Insertion-4

CATCATCATGTTTGAGCGTCGTTTCTAACAGTGACATGTTTTCTGATGTTTGGATAATGAATGAATACGGGAATGAA
AATTCTTGGACTATATTGTTACGGGTTCCTCACATGAGAGATTTTGGTTTTATTGGCTATCAAAGGGTATTATATAT
TTCAGAGGATAACCAAGTGCTAaTGGAGTTTATATCTTCGGGGAAATTTAGTTTGGTTATCTATGATTCCATaAATA
ATACATTTAAGATTCTACAaTAATtCAAAaCAACAaCAaTGATAAAaTGGCaCCACCaCAAGtCTACGTTGAGAGTT
TGATATCaCCTCAaCTGCAGCCTAgAGACTCGTCTTTTTCTTCgATGaAATGATCTTGTTAATTACC

NF11217XR108 9a-Insertion-5

CATCATCACCATCTGCCCAACAAGTCTCCGTTGCTACTAATCCACCTGTTGCCATATGACAAGTACCCCTGTTGTGG
CTTTTTCCATCTACTATATGCCTCTTGTTAATCTCCAAATTCTAAGTATTTTGCTGCTTTTTCTTTTGCCATTTCAA
AACAACAGGGAACTAAATAGCTTGTTGGATTGTTGCCAGGCATATTCCAAAGCTGCTTACTTCTTCTCTTCCATATA
TTCCACAACACCATAGTTAAACCATTCATAATCATTGTTGTGCAGTTGTTGATGACAGTAAAAATAAACTCTGCACA

TGATGAAAATGTCCCCTGCATGCGTTTGAATTTGAGGTCATAGGTCACTATTCCCCTAAGCTGTTGTAGTTTCTTGC
CATTCAAAGAAGATATGCCTTAGGAGGTGCTGTGTGTAGGAAGGGAAAGAGTGACTTCTGTCCCTTTTTGCAATCCA
CTTGTGTGCTTTTAATTCTCTAACGGTCACAGGTTCTGCTCGTTCACTGTCTTGTT

NF11217XR108 9a-Insertion-6

CATCATCATTAAGCTGCGTCATGGAACCAAACCATGAGGGAACATTTCCAGTGAGTTGATTGTACAACAAGATAATT
TCTTGAAGATTGGTGCAATTGGTAAGCTCAATAGGAATCTTACCATGAAAATTGTTCTTGCTCAAGTCAAGAACTTG
CAACCTCTTCAGAAGACCAACTTCTTTTGGAATTTCACCATGCAAATTGATGTTGGAAAGTTTGAGTATTCTCAGAA
ATGTTAGATTTCCCAACGGTGGTTGAAGAGTACCACCCCAATTTGATTTTCCAATGCAAGACAGAGACTCTCATGTG
GCGTCGACCGCATGTACACCCTCCTTCACAGAATGAGAGATCTTCCTGATGGAGATATCAGTATACATGGTAGTTTT
CTTCAAGAAGCAAGCTGTTACAGCACTGACTCAACTCAAGCAGACACTGTGTGACGCTGAGACACAGCATGGGATGA
GTCTCATTTGCAGTAGGGATGGGATATGGAACACATATCTGGGTGTTGTGATAATAAGAA

NF11217XR108 9a-Insertion-7

CATCATCAGTATGATAAATGATAAATATGAAACATAAAAGAGAAAAGTGTTGTTTTAATTAGAAGTTAGGTGACGGC
AGTGACGACGACGGAGGGAGGAAGAGGAAGAGGAAGAGGAGGATTTGAGTCATCTCAATGAAGACCCAGCAGAATAC
AAAGATGTGAGAACTAAAAGTTTTATTTTTTGTGTGCGACAAAATTGAAATGATGATCTCAATATTATATTTTGTAA
AAGTAATAAACTGTTTTGTAAATGCTATTCCCAAAATTAATTTGTTAATTTTGATCTGAAGAATAGAAATGTTTGGA
GTCTTTATGATCAAGATTCATTTTATACTTATATTTTATTTTTTTTAATAATTTTTATTTAAGTTCTGCAATTCTTT
GACCTATGTTGGACTTATCTCTATTAATAAGGAATTTTTTTAAAAAAGAATAAGGTTAATGTTTTTCTTGAAGGGAG
TATTTTTTTGGAGGTTAAGGAAATTAA

NF11217XR108 9a-Insertion-8

CATCATCAAATCCCTCAACACTTGGCCAATTATTCTCCGTGTATATTTTCCATACTCCTTCCTCATTCCCCAACTTT
TCTGAAAAATGTTCTCTTAGGCCTATAAAAACTACCTTTTGATCAATCAGATCCATATTCTCAGTTCACTAACCAAA
ACGCCATTCTTCTTTTGTTCTCTCCTCTCaAATTAAGTGCAATGGCAACAAGAAAAAGCAAAGCCATAGGCATTGAC
CTTGGTACAAGCTACAGCTGCATTGCAGTGTGGCGAAACAATCGTGTTGAGATCATTCCAAACGACCAAGGGAACCG
TGTCACCCCTTCTTACGTTGCCTTCACCGACACTGAAAGATTAATAGGCGATGCTGCCAAAACCAATTAGCCAAAAA
TCCCCACAACACTGTTTTTGATGCCAAACGTCTGATTGGCCGTCGATTCTCTGACCAAACAGTCC

NF11217XR108 9a-Insertion-9

CATCATCACATTTCAGTTTTACTGTTTCTGAACCCCACAGTGTTCAGTTCAGAGACAACAGTGATTCTAAACATTGG
CATTGGATTTGGACCATCAGATTTGCTTCAAACCAATTAAATCCAATCCACCATCAGTACTGTACATACCTATCAGT
ATTCGTGTATATGAACGTGTGTGGTATGGAATTCAGCAAAAATATATATAAATAAAAACAACAAATATGTATAGTAT
TTCATGCACACTATAGACTTGCAATTGAGATAGTGTATATGCAATCTTTGTAAAAAAAAGAATAGTTGTTATACATC
TAACTGAAAGAGAAGTTATTAAGTAGTCGATAAAATAAAAGACAACTAAAATTAAATACTACTTCTCCTCCCCAAGA
ACAATAAAGGCAAAAAAAAAAAACTCTGGGCGTCCTAAACCTCCAACAAAACTCT

NF11217XR108 9a-Insertion-10

CATCATCAGTTGATAGAGTGTTCATCGAGGCCACAGAAGAAATCAACTGCACATCACATGAAGACGAGGCCCAGAAA
ATCATCACTGTCAGATAGGAATCGGAAACCAACAGAGTATGCTCCGTTGCCACCACTGCCTCCTGATTTTACAATTG
TCATTTCTGCTGATGCATCTTCAACGGCTACTGTTGTTCCTCCACCCCCTACTCCTTCTACTTGATTCGGACTTTTT
TTTATTTTATTTTATTTTTAAGTATCTGTGTTGTTGTTGTGTTTATTTATTGGACTATGATTGTACTGTAAATATAC
TCGCTACTTGTTTCAGTTTATCTTCAATCATGTGATTTCATGTTACATTGATGTGACAAATGTCCACATGCTATTAT
GTATGAAACGCGTTTTCT

NF11217XR108 9a-Insertion-11

CATCATCAACAATTAAATTTAAAATGTTGAAAATTAAAGAACAAATTCTCATTTTTCCTTCTCACACCCCCTTCCTT
GAAGACCACACCCTCCCTCTCTCCCACCTCGACACCGACCGCAACCTCAATACAACCATACGCTACCTCCGCGCTTA
CACAGCCACCACCACCCACCACCATGATCCCTTCACCGTCATCTCCTCCTCCCTCTCAAAAACCCTCCCTCATTACT
ACCCACTCGCCGCCACACTCCGCTACCACAACCACCGCCTCGAACTCTTCTGCTCCAAAAGCCAAGACAGCGTTCCT

CTCATTCACGCCACCGTAGACATCACTCTCGAATCCGTAAACTACCTCGACGACGACCCATCTTCCCATTTTTGTTT
GA

NF11217XR108 9a-Insertion-12

CATCATCAGGTGTGATGCTCATGTGAGCAAGGTTTCCGTCAGATATATCATTATCATAAAAATGAGAAGTGTTATGA
ACACTCTCTTTTAAATACGATATCTAACACTTTTTTATTGGATAAAATTAATTTAAATCTCACACTTTTTAAATGGT
GCCTACATAGAGTGGAGTAACCCATATGAGTTTCATCTAACAAGAATGTGAATGTTAGATTGATGTATCCATGTAAT
ATGGTAGATTAAATATGGGTTATTTCGAATTTGATGATACAAAGAATTTATCTCAAGTTTCAAATGGCAATTTATAC
ATCAATGAATCAAATACAATGTGTTGGGGTTAAATTGTGTCACAAGAATA

NF11217XR108 9a-Insertion-13

CATCATCACATACATCTATGGAAGAACTAATTTCGTACAACTATAtTTTTTTATAGAAAAAATTGAGTGGATCATTC
ATTTTTTTTTTGAAGGATCATTCATAGCATGCAGAGATTACTTGTTTAAAATaCTTTCACATTCTTACCTTGCAGAA
ATCATTCTCTTATATCTAGTGTTTATGTGTCGaTTTCAAaCATCAGTTGGTGACaTCTTcTATTCTTGTTAATTACC

NF11217XR108 9a-Insertion-14

CATCATCAGATCTGCCATGTCCTTTCGCCGTGTTTATTCATTTTTGCCGTCAGCTCAACTGCTACTGTGATTTTCTT
GATTGATACTCATACATTCTTTATCAAAGTAGAGTCATTTAAAATCTTATTATCTGCATATGATTTGTATTTTTATC
TACATGTATTAACACTTGAGTTTCCCCATAAAAACAATCTC

NF11217XR108 9a-Insertion-15

CATCATCATGTGCCTCAGCTTCTGCTACATGTGAACTGCAATGACTTCTCCACTTTCTCAAAGATCTCCATGTGCAA
TGCACCAAGCTTCCAGTTTAGGTTTGTGACAAGTAAAGAGATCTTCACATGGCTGCTAATCTAGTCTGTCAGGAAAG
GATCAAACACTGGAAATTGATTGCC

NF11217XR108 9a-Insertion-16

CATCATCAACCCTCACCTGTGCCGGCCCCTGAAGCAGCACCGCCTTCAAATGCACCATGGGCTGCTAGCGTGCAGAC
ATCAGAGTTAACTTGG

NF11217XR108 9a-Insertion-17

CATCATCACTGAAAACTCTCATTTGTTTCACAGGTTCTGCTCGTTCACTGtCTTGTtAATTACC
