# Supplementary material for: Rapid identification of causative insertions underlying Medicago truncatula Tnt1 mutants defective in symbiotic nitrogen fixation from a forward genetic screen by whole genome sequencing
Source: BMC Genomics. 2016 Feb 27;17:141. doi: 10.1186/s12864-016-2452-5 (PMC4769575; doi:10.1186/s12864-016-2452-5)
Supplement: Additional file 3: — List of sequences of all NF10547 flanking sequence tags (FSTs) obtained from R0 and R2 generations by TAIL-PCR. (PDF 42 kb) [file 12864_2016_2452_MOESM3_ESM.pdf]

Additional file 3: List of sequences of all NF10547 flanking sequence tags (FSTs) obtained from R_0 and R_2 Generations by TAIL-PCR.
(Veerappan et al. 2015, submitted to BMC Genomics)

*The signature refers to the *Tnt1* border sequence flanking *M. truncatula* genomic sequence and its location at the 5' or 3' end of the individual sequence, if listed.

I. NF10547 TAIL-PCR FST sequences (R_0 Generation) obtained from *Tnt1* mutants database (http://medicago-mutant.noble.org/mutant/)

>NF10547_high_1   signature: TGAGATGGACATCATCA 5' end
TGAGATGGACATCATCAGGATGCATATTAGGATAATTTTAATCATAATATTCTATACTTATTAAGTTTGT
TTGTGATTTTCAAATAGACCCGCGTCGCACGGGTCAAATCTTAGTAATTAATTGGTTTATAGCACAAGGG
TTTCAAATGAATTAGGAGCTGGTAATCCATTTGAAGCACGTGTTGCCGTGTTAGTTGCTATGTCACTTGC
ACTCACTGTAACAAGTATAGTGAGCGCAACCCTCTTTGCATGCTGCCATGTTTATGGCTACATTTTCAGT
AGTGATACAGAGGTTGTTAAATACATTACTGTCTTGGCTCTTCTGGTTTCTATATCTGTTATACTCGATA
GCATACAAGGCGTCTTCACAGGTTCTGCT

>NF10547_high_10   signature: No signature
CTGCTGCTTCAAGTCTCTTTCTTCCTCTCATTCAAACCTTATCAGCCCAGGAAAATTGATTTTCCAAAAC
CATTCCCTTGTCATCGTCAGTTCAACTTCAATGGCAAAATTTCACTATTAAAATGAAGAACCGTGTATGA
GGTAGTAGATCCCCCCATACACGATAAAAAAAATAATTCATACAAAAATTCCATTGAGTTTGTTGCTTC

>NF10547_high_2   signature: No signature
GGCAGCAGATCCAGCAGCACGGAGGGGAAGGAGTTTGGCGGAAGTGAAAAGAGGAGGGGAAGCAATCCAT
CTGGCAATGGAATTTCCAATGTCGACGCTGGGACCCTCAGGACCCAAGGAATTACCAGTACCTAAAGTAA
TAGAAGCTGCAATAGCCTTTAAGAAAGGGTTGGAATTGGAATTGGAAAGAAGGTTGAGAAGAGAAACAAG
AAGACCTCCAAAAGCGGGAACAAGTATTACACTTTTCCATGTTTCTTGAATAGGAGCTTCTCTCAACCAT
GAGGCACCTGATGATGTCCATCTCATTGCGC

>NF10547_high_3   signature: TGATGATGTCCATC   3' end
ATTTATTAGTAAATTCACCACCTGCATAGATCAACACAATGCTTATGTATTTTTTCTCTTTTAATAGCCA
AAATTTTATTAGAAACTCAAACCAGTGCATCAATAATACACCAACTATATTAACTGCGCGAATTTGACAG
AATAACAGTTTGCCACGTCTTGGTTTCGGCAAAAACTACACTTTGAATCTTAAACAAAACCTCGATGCCA
TCGTGATTTTGTCAAATATATTGATTTTTTGGTCGGAGTACCCTTGATACTATTGTTAATTATGATGATG
TCCATC

>NF10547_high_4   signature: TGATGATGTCCATCTC 3' end
AGAGGAGAAGCAAAGGAACGATGGCTGCTTCGTTTTTGTCATCAAGTTTCTTAACCAGGAACTTAAAATT
TGGTGATACTAGTTACCGAAATCAAAGTATAGTTGGAAAAGATTCGTTCAATCATTTCAATTATGGTGGG
AAGAACTGGAGCGTTGGATCTACAGGCAAGTCTAACATTTTGCCTAACGTTACTTTTCTTCAATCAAGA
TTAGATCAACTGAAGTGGGAACAGGAACTGCGAAGAAAGGAAGGTTAGATATGATGATGTCCATCTC

>NF10547_high_5   signature: TGATGATGTCCATCTCAT   3' end
ATGTCCATCTCATTGACGGATCGTAATAATAATAACAAAAACAACAATAATAATAACTTTGAAGGTCCTT
AGCTTGAGCTTGATATCAAATCACCGATAGTCTTGTTAGCTGCATATTGTACATAACATTTTGTTTGGGG
TTGTCTCCAATGGAGAATTTGTCCAATCACTTTATCATGTTCATTTTCTATGCATATTATATTTGTTGAT
GAAATGCAAATCCTTAATGCCAGTTTTTGGTCGAGCATCTGTGTTGATGATGTCCATCTCAT

>NF10547_high_6   signature: TGATGATGTCCATCT   3' end
ACACGGATTGCCCGTCTAAACACTGATATTTGTTTTTTTAAAGGAAAACACTAGTATATTTATAATTCAA
GCACAACAACAACGTAGATTTTTTTAAAATATATATAACAATGTAGAAATTGTTTCAAAAAAATATTTGT
TATTGTTGTGAACTTTGAGATTTTTTTGGGTGTACCGTTAATGTTGAGATTTATTTTCACTTCCCTAAGT
TTTTTTTAAGTACTTTTATTATGTTAAAGAAAAATGATGATGTCCATCT

>NF10547_high_7 signature: No signature
TTATGGATCTTCCAATATTTGGATTGATCATCTTTCACTCTCTGAATGTGAGGATGGTCTTGTTGATGTT
ATCCAAGGTTCTACTGGTATCACAATCTCAAATTGTCACATGACAAAACATAATGATGTAAGTAACTTTG
GTTTGTTTAAAATGAAAAAATGAAATTTTATATGATATTTAATTTCTGCAAAATGTATATACATGGCCTA
CATTACTTAAAATTTCTGGGTCCGTCTCTGCTTCTCCACT
>NF10547_high_8 signature: No signature
TGGTGAATTTACTAATAAATGCATTTTCTTCTTCTTGATCTTTGTAGGTGGTCAATTTACAAGCACAGCT
AGCTTTTCTGAGAGAGCAAACAGCTCAAAGGTGTATCAATGCCCCAAATTCTGCAAACCCTAATGAGAAG
AACTTTGGAAAACCTACTAATATTCTCCCTCAAGATCTTCAAAGTTGGTTCCAGATGGAAAATTCCAACA
TGTGTTCTCAATTTCTTCCCGATTTCTCTGCTACTCCACT

>NF10547_high_9 signature: TGATGATGTCCATCTCA 3' end
AAAATAAAGATAGTGGGAAACAATTTATAAAGACACAGATAATTTATAAAGATGAAACAAGAAATAGAAA
ACAAAGACGTGTTAATCACAAAAACATAAAGAAGGCAACAAGTGTACTTGCTGAAGATCAAATACACCAC
TAACCTTCAAGTAAAAAATTATACACCTAACAAGAAAGTACAAATTTTCATTTGGGTATCGACGAGTTTT
ATGGAGGCCTTTTGATGATGTCCATCTCA

>NF10547_low_1 signature: ATGAGATGGACATCATCA 5' end
ATGAGATGGACATCATCACTTCATATGTATATTCCAATGGTAATTACAATGGAGTAGCTGATTGAATTAT
CATTTGATTGTAACATTATTTGAACATTTATGCCCGTATCAGTGCCTTTTTTCTTTATATATATCATTTG
TTTCTGTCCTCACTTTGCTTGTATTTGTTATACACAGATTTCAAAGTCGTTTATGGAGGGCAGAATTTCT
CTAGAAAAATACATATCTTCTTTGAAGTCTACTGTTGGAATAAACGTTCTTGTGGAAGCAGTAGGGATTG
GTAACAAGAAGGGAGACCTTACTAGGCTTGGTGTCGAACCTGGGAAGAACAGTCGGACGTTTTCAGCACC
AACTTGCAAATCTTTGTCTTCTCTTGGACCGAGTGATATATTACAATCTCTGCTTCTCCACT

>NF10547_low_10 signature: AATGAGATGGACATCATCA 5' end
AATGAGATGGACATCATCAGAGCTCAAGGGATGTTTGGTTTGGCATCTTTGCATGACCGTGGAATGGTTA
TGTTAATTAATCTTGCTTTCACTGAAGGTGATTTTGCTGGTAGTACTCTTAGTATGCTTGGAAGGAATCC
TGTCCAAGACACGGTTAGAGAACTGCCTATTGTCGGCGGCACCGGCTTGTTTAGGTTTGCTAGGGGATAT
GCTATTGCTAAGAGTGTTTGGGAGATTTCAACATCTGAGCATTTTGTTGTTGAGTATGATATCTCTGCTT
CTCCACT

>NF10547_low_11 signature: TGATGATGTCCATC 3' end
ATTTATTAGTAAATTCACCACCTGCATAGATCAACACAATGCTTATGTATTTTTTCTCTTTTAATAGCCA
AAATTTTATTAGAAACTCAAACCAGTGCATCAATAATACACCAACTATATTAACTGCGCGAATTTGACAG
AATAACAGTTTGCCACGTCTTGGTTTCGGCAAAAACTACACTTTGAATCTTAAACAAAACCTCGATGCCA
TCGTGATTTTGTCAAATATATTGATTTTTTGGTCGGAGTACCCTTGATACTATTGTTAATTATGATGATG
TCCATC

>NF10547_low_12 signature: TGATGATGTCCATCTC 3' end
AGAGGAGAAGCAAAGGAACGATGGCTGCTTCGTTTTTGTCATCAAGTTTCTTAACCAGGAACTTAAAATT
TGGTGATACTAGTTACCGAAATCAAAGTATAGTTGGAAAAGATTCGTTCAATCATTTCAATTATGGTGGG
AAGAACTGGAGCGTTGGATCTACAGGCAAGTCTAACATTTTGCCTAACGTTACTTTTTCTTCAATCAAGA
TTAGATCAACTGAAGTGGGAACAGGAACTGCGAAGAAAGGAAGGTTAGATATGATGATGTCCATCTC

>NF10547_low_13 signature: TGATGATGTCCATCTCAT 3' end
ATGTCCATCTCATTGACGGATCGTAATAATAATAACAAAAACAACAATAATAATAACTTTGAAGGTCCTT
AGCTTGAGCTTGATATCAAATCACCGATAGTCTTGTTAGCTGCATATTGTACATAACATTTTGTTTGGGG
TTGTCTCCAATGGAGAATTTGTCCAATCACTTTATCATGTTCATTTTCTATGCATATTATATTTGTTGAT
GAAATGCAAATCCTTAATGCCAGTTTTTGGTCGAGCATCTGTGTTGATGATGTCCATCTCAT

>NF10547_low_14 signature: TGATGATGTCCATCT 3' end
ACACGGATTGCCCGTCTAAACACTGATATTTGTTTTTTTAAAGGAAAACACTAGTATATTTATAATTCAA
GCACAACAACAACGTAGATTTTTTTAAAATATATATAACAATGTAGAAATTGTTTCAAAAAAATATTTGT
TATTGTTGTGAACTTTGAGATTTTTTTGGGTGTACCGTTAATGTTGAGATTTATTTTCACTTCCCTAAGT
TTTTTTTAAGTACTTTTATTATGTTAAAGAAAAATGATGATGTCCATCT

>NF10547_low_15 signature: No signature
TTATGGATCTTCCAATATTTGGATTGATCATCTTTCACTCTCTGAATGTGAGGATGGTCTTGTTGATGTT
ATCCAAGGTTCTACTGGTATCACAATCTCAAATTGTCACATGACAAAACATAATGATGTAAGTAACTTTG
GTTTGTTTAAAATGAAAAAATGAAATTTTATATGATATTTAATTTCTGCAAAATGTATATACATGGCCTA
CATTACTTAAAATTTCTGGGTCCGTCTCTGCTTCTCCACT

>NF10547_low_16 signature: No signature
TGGTGAATTTACTAATAAATGCATTTTCTTCTTCTTGATCTTTGTAGGTGGTCAATTTACAAGCACAGCT
AGCTTTTCTGAGAGAGCAAACAGCTCAAAGGTGTATCAATGCCCCAAATTCTGCAAACCCTAATGAGAAG
AACTTTGGAAAACCTACTAATATTCTCCCTCAAGATCTTCAAAGTTGGTTCCAGATGGAAAATTCCAACA
TGTGTTCTCAATTTCTTCCCGATTTCTCTGCTACTCCACT

>NF10547_low_17 signature: AATGAGATGGACATCATCA 5' end
AATGAGATGGACATCATCA CACCAAACCTCAGTTACGGTTAATTCAACCGTTGAGGTTATGTCATTTCTG
CATCGAATCGGAAACTTTAGGCTGAATACCGAGAGGACATTTCAATTTCGCAGATGTTACACTCACCTGC
TTCATGCTTCTTTCTCAAATTCTATTCCCAAACCTAAAACTCTCCCTACTGTTATGCCATATTCGCATTC
GCTTTTCCATAATACTAGACCCATAAATGTTT

>NF10547_low_18 signature: TGATGATGTCCATCTCA 3' end
AAAATAAAGATAGTGGGAAACAATTTATAAAGACACAGATAATTTATAAAGATGAAACAAGAAATAGAAA
ACAAAGACGTGTTAATCACAAAAACATAAAGAAGGCAACAAGTGTACTTGCTGAAGATCAAATACACCAC
TAACCTTCAAGTAAAAAATTATACACCTAACAAGAAAGTACAAATTTTCATTTGGGTATCGACGAGTTTT
ATGGAGGCCTTTTGATGATGTCCATCTCA

>NF10547_low_19 signature: No signature
CTGCTGCTTCAAGTCTCTTTCTTCCTCTCATTCAAACCTTATCAGCCCAGGAAAATTGATTTTCCAAAAC
CATTCCCTTGTCATCGTCAGTTCAACTTCAATGGCAAAATTTCACTATTAAAATGAAGAACCGTGTATGA
GGTAGTAGATCCCCCCATACACGATAAAAAAAATAATTCATACAAAAATTCCATTGAGTTTGTTGCTTC

>NF10547_low_2 signature: TGAGATGGACATCATCA 5' end
TGAGATGGACATCATCATAAAGAAACCTTAAGATTGCACCCACCTAGTCCTCTACTACTACCAAGAGAGT
GTATTGAAACTTGTGAGATTAATGGATACACAATATCATCTGGAACACAAGTGTTTGTGAATGGATGGGC
AATAGGAAGAGACCAAAAGTATTGGAGGGAAGGAGAAAAGTTTTATCCTGAGAGGTTCATGGATTGTCTA
GTTGATTACAAAGGGTCTAATTTGAGTACATACCTTTTGGTGCAGGAAGGAGAATATGTCCAGGAATCAC
ATTTGCTGAACCTAATTTAGAATTTCCTCTTGCTCAATTGTTGTACTATTTTGATTGGGGACTTCCCTAT
GGAATTACTCATGAAAATTTGGATATGACTGAAGTCTCTGCTCCTCCTCA

>NF10547_low_3 signature: TGAGATGGACATCATCA 5' end
TGAGATGGACATCATCAGGATGCATATTAGGATAATTTTAATCATAATATTCTATACTTATTAAGTTTGT
TTGTGATTTTCAAATAGACCCGCGTCGCACGGGTCAAATCTTAGTAATTAATTGGTTTATAGCACAAGGG
TTTCAAATGAATTAGGAGCTGGTAATCCATTTGAAGCACGTGTTGCCGTGTTAGTTGCTATGTCACTTGC
ACTCACTGTAACAAGTATAGTGAGCGCAACCCTCTTTGCATGCTGCCATGTTTATGGCTACATTTTCAGT
AGTGATACAGAGGTTGTTAAATACATTACTGTCTTGGCTCTTCTGGTTTCTATATCTGTTATACTCGATA
GCATACAAGGCGTCTTCACAGGTTCTGCT

>NF10547_low_4 signature: No signature
TAAATGTTTCTCCAATTGTTTTAAACAGCGTGAGGTGTTATGCTGCACCCGTTCAGGTAATTTACAAACT
TCTAGTAGTTGTAACTAGGGCTTAATTATGAACAAAGAAACCTTAATGCATGAATGTTCCTTAGAAACAA
TCTCTTTTCAACACTATCAAAATTGTCATCTTTGCTATTGGGAATATGTTCCATGGTGATTTTGGTATTG
TGACAATCTGCGTCTCTTGGTTTTATTCATGTCGCGCATTTTGTGTTTGTGCCTTCAGTAAGCTTGAGCA
TTATCCTTCACGTTATGTGTTTGGTAGCTTTGCTATGTATTGGTGGTGTTGGTTGATGATGTTTTTCTGT
GTTTTCTCTTTCATCTCACTCGACC

>NF10547_low_5 signature: TGAGATGGACATCATCA 5' end
TGAGATGGACATCATCAATTTCTAATTTTATAACAAATTTTCAGGACAACAATACAAATTGAGTATCAAC
AACTATTTTTATTAATATATTTATTGCTCGTTTTCCATTTTAAATATAAAAACCTTAGAGATATTTTATA

ACTAATTGCAAGTTCTCGTGGTGTAATCTAATGGTTTGTTTCCTATGTATAATGTTCCGAACACCAAAGC
AACATTTATTACACAAACAGATTCTTCACCACTTTCACCTCAAGACTGGCGAAAAGATAGTGTACTTGGT
GAATCAATCCTCACAATAATATCTTCTTCAATAGCTGAATCATCACTTGAATCACCTCTTCTATCTCTGC
TTCTCCACT

>NF10547_low_6 signature: ATGAGATGGACATCATCA 5' end
ATGAGATGGACATCATCAACACCTTCATCAAGTTGCTCCCCCTGCTCAATAACCTCACCAGGTTGCTCCC
CCTGCTCGGAAACCTCGTCGGTCGTACTTTCTGCACTTGTGGGATTGTTAGAAGTAGAAGGAATAGTAAC
AAAGTTAGGAATTATACCATTCTTCGCCTTTTCTGACATATCAGCAGCAGTTCTAACTTCACTTTCTCGG
AAGACTACATCTCTACTTCTGATGACCTTCTTCTTTACAGGATCCCACAGTCTGTACCCGAACTCTTCAT
CTCCATATCCGATAAATATGCAGGGAATAGATTTATCATCCAGCTTTGTTCTCTGCTCCTCCTCAAGAT

>NF10547_low_7 signature: TGATGATGTCCATCTCATT 3' end
GATCTAGAGGAGAAGCATAGGACCCATGAGATGATATGAAAATATTAAGAGGTAGGAACTGTGGAACATA
CCTGAAATGGGAACACAACTTGTCTATCATATTGTGACTTCTTCATTGATATATATGAAAGGGATGTGAG
CAATTTTGGACCAATGTTCCTTCCTTTTATACCTTTCTACTAACAATGGACATCGCACAATGGACAGTTG
CTTAAGAGAGTCAGGGAGGCTGTCTTCTGGTAATGACTCAAGCTTTTCACATTTCCACAATTGAAGTAAT
TTCAGCGAGGAAGGGAGTGATGATGTCCATCTCATT

>NF10547_low_8 signature: TGATGATGTCCATCTCATTGCGC 3' end
GGCAGCAGATCCAGCAGCACGGAGGGGAAGGAGTTTGGCGGAAGTGAAAAGAGGAGGGGAAGCAATCCAT
CTGGCAATGGAATTTCCAATGTCGACGCTGGGACCCTCAGGACCCAAGGAATTACCAGTACCTAAAGTAA
TAGAAGCTGCAATAGCCTTTAAGAAAGGGTTGGAATTGGAATTGGAAAGAAGGTTGAGAAGAGAAACAAG
AAGACCTCCAAAAGCGGGAACAAGTATTACACTTTTCCATGTTTCTTGAATAGGAGCTTCTCTCAACCAT
GAGGCACCTGATGATGTCCATCTCATTGCGC

>NF10547_low_9 signature: TGATGATGTCCATCTCATT 3' end
AGTGGAGAAGCAGAGACTGTCCATCAAAACCCTGAGACATGGCTGCCGAATAGTTGGCACCCCATTAAAA
TCCGAAGGTAGCAGAAACTGCTTGTATGTCTCTGTTGAAAGGTCAAGTGAAACAATCGAAAATCGCTCAA
TAGACTTGCGAGTCAACCAATTAACAGTGCCATTCAAATGCACACCATCATTATAGTCATGGTATCGATA
ATAATTATTGAACCATTTAACAGGAACAGTATTAAAGCTCTGAATATTTCTCCAACTATTATTACCTACG
CTGTGATGATGTCCATCTCATT

II. NF10547 FST sequences obtained by TAIL-PCR from R_1 generation

NF10547-F-1
CCAACAAATTAATTAATTTAAATTCTTCTTTGCAATTATGAAGAGAAAAAAAATGTAAGCGTATACCATCT
AGGGTTTTCAGTTTAAGATCACGAGAGATGAACAAACCTGTTGTTGGAGTGCAAAAATATGGGCAACACAT
CCATAAATTGGATCTTGAAGTCTAGCTTGAGCTTCATAAGAGATTGTGACTGCAGCTTCACAACGATCGGT
CACAGGGAGGTGTGCAAGAAGCTTTGAAACATTACTTGCACCAAAGACCTTATGAATAGCTGCAAAATGGG
TAGCACCTTGTTCATGGCAAAAGTAAGGTGCAAAAACACAACCTCTAACACATTTTCTCCTCAAGAATTTG
CAGGCTCCACAAGGAGAACCAGAACCAGTCATCATGGCCGTAAGAATTTTTGGTTGTTTCTGGCTCTCTTG
TGCTAATCATAATCATTTATCACCATCTTTATAGACAAAAAAAGAATTAAACAAGAGGAAAGGGAAGATTA
TTAGAACGATGAATATTTTAATTACATAAGGTTCCCTTAATGGCCTACATGTTAATTATTTTATTTCATAT
AAAACTATATCACCAAATTTTTATTTGTTAGAAAATGAGATTCTTATTTAAAAACATCCTTATGTGACAAA
ATTAAGAGATAGCATTAACCAAACCCATTTTGTTTGATGATTGGATCTAGCTATTGGCTCTTGTCTTTTCT
GTTTCTCAAGAATTATGTATATAGGGTACCTCATATATGATAGCGGGCAACGGTTTTCAATTACTACTTTT
TTTATTTATTTCTTTATATAGATGCACTTTGACCAAGAGAAATTAATATGGGCAATGGAATTAAATAAAGC
AAAGTAGGTTAAATAATAATTGTATACCCAAGGCACCTCTTAAAAAAAATGGTAAACC

NF10547-F-2
GAAATTTAAAATTTCCGATTTTATTTTCCAGGAAATAATTTTGGCCACAATGCAGTGTTTTGGAAAATCAT
CAGTCTAAAAAACACATCACAAAATTACAAACAAACAAACATATTGTTCCCTATATATGGTTGTATCCACT
TGTTTATTTTTGCTAAGGGAAGACAATCACTGTTGTCCAAGTGAAGGACCGTAGAAAACATGACTTTGTGC
AATTGCAATGCGTTTTTGTGGATCATTCAGATTTTCATCCCAATGTGTTGACGGTTCCCTAAAAACCAAAC
ATATCTATCGTCATGACAATTTTTTTAAACATTTTTTAATTCAAAAAATATACATTTTCCTATCGTCAATG
TTACTTTTCAAACAAAAAAAAAAAAACTTGGGTATCTAGTGGTCATTTGCATCATTCAATTCTAGACATGAA

AAGGCCTCGAGACGTGAAAATGAAAAGATACTTCAAATAAACATATTCGATGTTAAAATGAGTTAAACTAA
ACGAGTTAAAATTGCGACTAATTATAAATAAAAAAGATAAACTACAATATTTCTTAATCACAAAATCCCAT
GTGATTTATCAACTACAAATTAGCCGATCATAAATTAAAAATAAAATAAAAATTAACGTAATATTTCTTGA
TCACAAAATCCAAATTGAACTCTCACAGTTCATATTTCATTTTCTTAGCAGTCTCGTTGAGCACCTCCTGT
TCTTCAGGTTGATTATCACCACTCCATGCACTCTTTGAATATGGAGCTCATTAGAATCAGACACTTTTCAC
ACAAACTTAGAAACAAATTTTGCAACATTGGGATCAGTCTTATCACCAGGAAAACTTCAAAATCATCCCCT
TGAAGTGGTTCTCAATGTTTTCTTTTCTAACCGGATCATTCAAGTACG

NF10547-F-3
CCCAACACGCTGAAAACTTTAACCACACTTTTCCACGAAGCTTCATCAACACGAATAACTACAACCTTATA
AGTTCTCGTTGAAGCATCATAGCCAAAGGCTAACTTAGAATCGTAGTAAACAGAACCGTTGGGTGGAGTGA
GCCCGAACTCTTTAGATCTTGTTCTGGTGGCAGGGTTCCAGAAATGGAGCCAATACTTTAAAAGAGCCGAT
GGGTATAATGTTATAGAAGTGAAAAGCAAACAGAGTGGTCCGTTACAAGAACCAACAATCGTGCAATGTTT
ATTGAAACGTTGGAAATTGTGGCTGTAAACGTTGATGGATTGATTTTTGAGTAAACCATGCACAGGGAAGG
GTAGGACATTAACACCCTCTTTGCGTTGGTTCCAAAATAAGGTGAGGTGTGGGTTTTGAGACGACTTCTTA
AGGTGCTTTTCTATGAAGGATGGATCAGATATGAGAGCATTCCATGGCTTGCTCAGGCATTTGAGTCGAAC
AATGTTTTTAACCCTAAGCAAAGATAGGATTTCTACGATGAGTTCGTCTGGGAGGGAACAACTTCGGGATA
TGTTGCATTGGATCGACTGGGTCGGCGTCGTCGTTGCTTCTTAAATTGGAGGGGCGTCCATCAGAGTATGG
TGAAAGTTGAAGAAGTTGGAAAAAAAAAAACCCCAACACATGCAACTATCAAACGCAAATCAATTTCTTTC
TGCAAAATGCGGTTGAGAAAAGTTGTTTTATACGTACCCAAACTAAAGTTTGTGCAATGCCTAATATATAA
CTATTAATAAAATGGGGCCCAAGCTTTGGCCAGTGTTGCGGGCCCCCAGGGTTGTGTTGCGAAGAAGAAAT
TTTTGGGGCGTTTTTTCTTTTAAGGGCCACCGGAATAT

NF10547-F-4
CCCAACACATCCAACAACATTTAAAAACAAAAAGAATATATGATATTGTTACATGATTACCATTTCTTTTC
TTTTTTTGCTAATACATGTTTAACATTTCTAAAGATACTTTTAAATTAAAATAATATAGGATAAGTTCACT
CAATCAAACTTATATCAAAGTAATATAGAATTTATTAGTTCATAAATTATAATTTTATTAATAACTTCGAA
ACTCGGCATCGCATTGAGAATATTCTACTAGTTTCTATGGGCAAAAGTTATGGTGCATAAGCCATAACTTA
TGCACCATGCATAAGGTCTTCACAGCCATCATATCAATTGGACACTTGTAATAATCCTACGGTCTTCAGCT
CAGATCCCACATTCAGACCCCACATTCATTGATTCAGCTGTGCCCGACGAAACCAAACCTCACAACCTCGC
CGGAAACCACCGTGATTCAGTTGATTCATTCATTGTTTGTCTTCATTACCGGCGCTTTCTAACCTCGCCGG
AAACCACCGTGATTCAGTTGATTCATTCATTGTTTGTCTTCATCACCGTAATTCAGTTGATTCAATTGAAG
AGGTAAATTATGCACAACTTAACAACTCTATATTCACTTCTGAAACGTTGTGTTGTGATGAATGTTATGAA
TCATGATTATGTTAGTTTTTTCCAATTTCTACACTCTATTCCTAGCTTCTGTTACTTCCCCTTTTATTACT
CAAACTAGTAATAATGCAGCATGGTATTTCTGGTTACTAGGATTCACATATTTGTGTAAATTGGGATTGTA
ATTGTAATTCAATTTCTTGGGTTTGACCCAATGTGGTTTTCCGGCTACTTCACGGCGTGATT

NF10547-F-5
CCCAACACATCCAACAACATTTAAAAACAAAAAGAATATATGATATTGTTACATGATTACCATTTCTTTTC
TTTTTTTGCTAATACATGTTTAACATTTCTAAAGATACTTTTAAATTAAAATAATATAGGATAAGTTCACT
CAATCAAACTTATATCAAAGTAATATAGAATTTATTAGTTCATAAATTATAATTTTATTAATAACTTCGAA
ACTCGGCATCGCATTGAGAATATTCTACTAGTTTCTATGGGCAAAAGTTATGGTGCATAAGCCATAACTTA
TGCACCATGCATAAGGTCTTCACAGCCATCATATCAATTGGACACTTGTAATAATCCTACGGTCTTCAGCT
CAGATCCCACATTCAGACCCCACATTCATTGATTCAGCTGTGCCCGACGAAACCAAACCTCACAACCTCGC
CGGAAACCACCGTGATTCAGTTGATTCATTCATTGTTTGTCTTCATTACCGGCGCTTTCTAACCTCGCCGG
AAACCACCGTGATTCAGTTGATTCATTCATTGTTTGTCTTCATCACCGTAATTCAGTTGATTCAATTGAAG
AGGTAAATTATGCACAACTTAACAACTCTATATTCACTTCTGAAACGTTGTGTTGTGATGAATGTTATGAA
TCATGATTATGTTAGTTTTTTCCAATTTCTACACTCTATTCCTAGCTTCTGTTACTTCCCCTTTTATTACT
CAAACTAGTAATAATGCAGCATGGTATTTCTGGTTACTAGGATTCACATATTTGTGTAAATTGGGATTGTA
ATTGTAATTCAATTTCTTGGGTTTGACCCAATGTGGTTTTCCGGCTACTTCACGGCGTGATT

NF10547-F-6
CCCAACAGGGAGGCAGTTTTCTGGCAATGTCTCAAGTTTATGACAATACCAAAAACTGGAACACAACTTGT
TCTCATGATATTGTGACTTCGTCATTTATATCTATGACAGGGATGTGAGCAATTTTGGACCAATGTTCCTT
CCTTTTATACCTTTCTTCTAACAATGGACATTCCCTAATTCTCAGTTCCTTAAGAGAGTCAGGGAGGCTGT
CTTCTGGTAATGACTCAAGCTTTTCACATTTCCACAATTCAAGTAATTTTAGCGAGGAAGGGAGGCAGTTT
TCTGGCAATGTCTCAAGTTGATGACAATTAAAAAACCAGAGATTTTGGAGAGAGGAGAGGTGTCGGAGCCC

ATTTCCATCAAAGGACTTCATTTCAGAGAGATAATTGATATCCAGAAACACAAGGGAGATGGGTAGCAATG
ACTCCTTCATCAAGGTGTTAAATATATCATCACCCTTTACAATATTCAATCTTGAAAGTGCTGTCAAATAT
TGGAGACCCCATTCCGTAACCGGCGGTGTTGTTTTTTGGGTAGAAATGTCAATTGATTGTAATTTGGGAGG
TAGACAAACTCCTTCAGAAAATGACAGCTTTTGACATTTCATATGCAACTTTTCAAGAGCGCTGAGCATGT
CCATCTTAAACTTGACTTCAAATAATTCAATTGAATCATGGGATGTGATAGTAAGTGATTCGAGGCTCGAC
GACCGAGGTGAAGACCTTTCTGAAATATAAATGGAATCCAGACTCCTACAATCACGTATGGTAAGTGTTTG
GAGCGCAGGGAAACCATCAAGTGTGAAGGATGTAAGTT

NF10547-F-7
CCCAACATCACGTTCACTCACGTGTTTATAAGAAGAATAGTAGTAATTGATTAATGTCAAATGTAACCGGG
CTGGTTGTTAGCACTTAGCATGGCTTTGTTGGATTTGGAGCATTTCCCTGAGAAGAAGATAACGCAAGTTG
GAAAAGAACATCTTGGGAAACCAAAACCTACTACCTGTTTGTCTTGGGACAGAGTAGTGTGGGAATGGATC
TAAGCTTAGTACTAGTAGTAGCTGTGTCATATCATATGCATGCAATTACACTTTTGGATAACATACCTAAA
AACATGTCACGACAATGTTTAATGTTGTGTTGTGTTGTGATGTGATTAATTAGTGGTACTTATATGGGAAT
CGCGTTGGATTCACGTTATTCAACAAAACCAATTTATTTTTGCATATATATAGTCATTTAGTAGGTGGTAT
TGGAGCAACCCACATTTCCTTTGCTATTTCTATATGCTATACTCTTTGATACTACTAGTAGTGAAAAAAAA
TGCTCTTCAAGTCATTATTGTAATGGTTGAGGGTGAGTTTTCAGAATTCATATATGCTTTCTAGTGTCTAG
TGGGTGTTATGAAAGCACTCTCTAACCATATTTAAGCATCATATTCTGTTGCAGTCCTCACCTATGATTCT
TTCTTTTCTGACCAAAGCCCTGTTTGATCTAAGTAACTACTACTTTGTGTTGTTTCTGTCATATTTGTATG
TAAATAACAAGGATTTTGCTTTTTTGTTAATTGGATTAAGATTCCCTTTGGTTTGGGAATTCAATTCTTAT
TCTTCCCTTAGTTTTGGGGG

NF10547-F-8
CCCAACACTACCATCAGCAGCTGCAGGAGGATCTTGATACTAAGCAATTACTGCTACATTTCAACAACAAC
AATAATCAAATCCTACCTCCAAGACCAACTGTGGCCTGGAACAACAACAATTCTTCATCATCAACACTCCT
AGAAAGGGGTGCTTTCAGAGTGAAGGCAACTTTCGCGGATGAAAAAATCAGGTTTAGCTTGCAAGCAATGT
GGGGATTTAGAGATTTGCAGCTTGAGATTGCAAGGCGATTTAACCTAACAGATATGAACAACTTGGTTCTA
AAATATTTGGATGATGAAGGAGAATGGGTTGTGTTATCATGTGATGCTGATCTTGAAGAATGTAAAGACTT
GCACACATCATCTCACACACGTACCATTAGACTCTCTCTTTTTCAAGCTTCCCCTCTCAATCTTCCAAACA
CTTTCCGCAACAGCAGCAGCAGCAGTCCATCCTCCTAGCTAGCTTACAACTTCTCATCTGAATGTGTTGTG
TCTGTCTGTATATTGTATAGCATCCAAATTCCCAAGCAAACATAACATCAATGTTAGCTCATATACCTTTA
CTGCTTTTCATTTAATTACTCGTTAAGTTAGATTCTGCGGCTTTATTTTGTTAATTACACATCATATGTTT
AATTAGAGTTTTAGTTAACAAAGCATCATACCAAACATGTAGTCTTCGTGATTACATAAACTATTTTG

NF10547-F-9
CCCAACAGTCGAGAGTTCCAAGTTTGATTAGAGATCAGTGTCATAAAAGTTGGGGAAAAGGGGTCACGAGA
AGCTTGGGTTTGTTTTGGAAAATTTATTCCAAAAGACTTGGCAATGAATCTTGCATGGTTCAAATGACAAC
TTGGGGAGTTCATTTGTTGGGGCAATAGTTGCACTCACTTCAAGTTGAACATAAATATATGATGATGTGTG
TGTTTGTGGAATAGTATACGACCTCATGTTTAAATTTGAATGTATTATTTTATCTTGGATGTTGTTAGAAT
TGTTTTTTCTTTCACCAGATGAATTTAATCAAAGAGGGGTGATTTAATGTCAATTTTTTTACCCCAAACCA
TCCCTCTTAATCGTTTTTCATTACCTTTTATTTAAATAAGTGATTTTTACATATATTTAGGTTTTTGATTT
GTGATTTCTTCGTCTTACTGCAACGTTGTTTTGTTC

NF10547-F-10
CCCAACAAGATGGTTCTGCAAGAAGAAAACAAAAGCAATATAGAATTAAACCTAAAACGAAAACAAGCAAA
AAGAAGCATATATTGTTTCAGGGAAACGTACGTACGTATATAGCAACGAGTAATCGTCAGCAGTGAACTGA
GGAGTCTCATGAAAGCCTTCAAAGCCTTCTAGATTGTTCTTGAGTTTCTTGATACCTTGCTGGATAAAACC
CCATCCTTCTTCAAAGTTGATAATCTTTGACATGTTGGTGAATTGAGAGCTGCAATGAAATATTGATTAAA
AGAATTTCCAAAGAACTGAAGAAAATAGAATTGAGAAGAATCGCATGAAAAGAAGTAGAAGGGTGAACCTT
GGAATCGCAGAGTGAAGAAAGTTTGTTTGATGCAAAGCGAAGTTAACCTATAGCTATGCATGCCTTTTGAT
CGTTTTATA

NF10547-F-11
CCCAACAGACTTAACAAGGTCCTTGGTGTTGATAAAACGATCCTCGTTAAATGGGTTTCCCTCAAGTCGTA
TAGAACGTTTCCGTGCTCATCCACACAGGTATAAGGCGTTTCCTCTGCCTAAACAAGCCTTCGCTTGCTGC
TTTACTATGGATGCTGTCAAAATACTTGCCTCTGGCTTTGATTGTCCATGTTTGCATGTTCCAATACTCTT
AGGCGTGTCGCAAGTTGCTTTTTGAAGGGTATTTTAATTTTTTTTAAAAGAATAACATGATATTATATTCT

CATATATGTATCATATTGTTCTTTTAATATTTTGAAAAATATCAAATATATATTAAAATATAGACTTAACA
CCCTCCAAAACCCTCTAAAACCCTACTCACAAACACACCCTTAGGCA

NF10547-F-12
CCCAACATCAATCTCAATTCTCAACATATTCCAGATATGTATTCTTCAAATAGTTCCCTCAATGGAAATAA
CCTAGCATTTTCCTCTAAATCACCTTTTTGTTCCTTTGAAAGCAACTCAACTTCATCAAAAGATATTGAAA
ACAGTCATTCCCCCTTTCCTCTTCCCAGTCCTTTCTCTTTCTTTCAATTCCCTAATTATCCTGATGATGAT
ATTGATCCCTTTCAAGACAGCCATCAAATCTTCCTCCAACAACATGATGTTGATTTCCAACTTCATCATCC
ACCTGTTGTCATGAATAACAACAGTACTGA

NF10547-F-13
CCCAACAATCTACTATTCAGAACCTCGATATTGAGTTTCAATACTTCAGAAGAATAATAATAATGAAAGAG
TATTGAGCTGAGTTCCTAGAATTTGGCAAAATATTCAAATTTCATTTTGGATGTTAAAAATTATCTTGTAA
GAAAATAATCTAACATATGATTTATCAAACAATAACTAGAAACATGTCCGGTTTCAATGACCTGTATGTTT
AAGTGATTTATTACTCAAAAGAGGAGAGATGTTAAGAACAACACCTAAAGGTAGAAGTTCTGTTAATCTTC
CATGATCCTTTTCTG

NF10547-F-14
CCCAACATCAATCTCAATTCTCAACATATTCCAGATATGTATTCTTCAAGTAATGCATAAACATTATATCA
CATCATAATCAACAACAACATATCTTATACAGCTTCACAGATTATAATTAACCTCATTGTTAAGTCTTAAT
CCTACCCAAAGGTTCAACTCTCGACAACTCGT

NF10547-R-1
CATCATCAATCTAGACATGTGAACAATAATGATAATAACAGTAATATTCTTTCTAAATAATGTAACTAGAA
ATCAACTTTGTGTACCCATTACAGGAAAATTGCATTACATGATATCATTTAAACTATTAAAATCTCCACCA
ATAATCTCTTCGAGTCATGGAAACGTAACCTCCAAAAACCATAAGTAATTATTCTATTCGCATGGCAAAAT
TGTGATATCATTTCCAATCCTAACCCTATGTGATTTCCCTACTCGTATTCCATGTTCATCGTATTCCATAA
TCATCATTATTTATATATCTTTCTTATAATCCTCATGTTCATAATTATCAATGTTCTCATTATTCCATAAG
GCATTTTAAACATATAAAGTACGTAAATCATGTTATGAAAAAGGAAATATATATCATTTCTAACATATATC
ATATAAAAAATGATTTTATGCATTTTCCTACTATCACAAACACATGCAAAAAAGTAAAACAACACAAACAA
ATAAAAAAAATCAATATTTTGTTCAATCTTGTGAGAAAACCTATAATGAAGACATATCTCTCAAACAAAGA
TACTAAGAAATCACAAGATAATCCTTGGATATTATTTATTTATTCCATCCATAGTAGGGCTTCAGTGACCC
TTCACATATTATTTGAAATAAAATTTACCTAACTATTCATAACATGTATTGAATATTTTTCCCACTTGAGA
ATAGTACATGAATGTGTTGGAACATTAGTGAGTTCTTGTGGAAAGTTGGAAAGAATAACACATAGAAAGAA
CTAGTGTTGGTATGACTTAAAAATGTCTCACATTGGAGAGGTACATACTACCTAGTAAGGTGTTTACATAG
TGAGCGGTGACTCGAAGG

NF10547-R-2
CATCATCAAATTTTACATCCATAAAAGCTTACAATTTGTTTAATGGTTGCATTATCATTGTGTTCATCTTA
AAAATCTATTAAAATGCTTGAAAAATCAAGTCTTTATGTCTTTCTAGCATTGGTGAATAACATGGATTTAT
ATCAATATCACAATTGCATTTGCTTACTAATCTATCAGTAATGGTTTGTATTATTTGTAGATTTCATTTAT
ATTGAACTTGCTACCAATTCCAGGTTATTGATTTACGAGTATGTTAACAATTGGAATTTAGAGCAATGGCT
TCACGGAGCAATGCGGCAGCATGGCTATCTTACTTGGGAAGCTTGGATGAAAATTCTTCTGGGGCTGGGAA
CAGCCAAAGCGTATGCTCGTTTTTTAACTAGTTTCCAGAGTTTATTTTGTTGAATGAGTTTCCTTTTTATC
TATGATTAATTTGATTGATTATTGTATCTTGTAAGGTTGGCTTACTTGCACGAGGCGATTGAGCCAAAAGT
TGTACATCGAGATATTAATTCGAGCAATATTCTAATTGATGATAGCTTTAATGCTAAAATTTCTGACTTTG
GGCTTGCTAAGTTACTTGGTGCCGGGTACTTTCGGGTAAGCTTCATGTTCCACATTTAGAATTTGTTTCCT
TTTTAAGATTTAGTTTCTTTAGAAATTCTTATTTCATTAGACACGGTGACGTGCATTTTTTCCATATTGCA
AATTAAGAAAGTATATAATTATTCACAGTCTAGTCATAATTACAATATATCCATATTAAGTACTATATTTT
TCCTTTAACAATCTGGAATATTTCTGCCAAAACCATTGCATTTA

NF10547-R-3
CATCATCATGAGGAATATGAGCTATCTTGTCCCAATCCTCCCCTGTTCCCTCTTTGCATCGTTCCTCTAAT
GTTGGGCAACCAATGATATTAAGAAGCTCAAGAGAAGTGAGGCGTTGAATACCCTCCGGCAAGCATCGCAA
TCCTTCACAATCCCAAATCGTTAGAATCCTAAGGGATTGAAGACCTTCCCTAATTTGCTCTGGTAAGGACT
CCAACTTATTACAATAATTGATACACAGATGCGTCAAAGCTGGATTAAAGGGTTCATTTGGTAACTCCTTC
AGTTCTCGGAAATCACGTACCACCAGATATTGAAGAGAAGTAAGGTTCTTGAACATCTCCTCTGGTAAGGA

TGTTATTCCTTCACCGCCAAATAGATAAAGCAGAGTAAGACCACGCAAGGTAGAGATTGACCTCAGTAACT
CATTGTTACATTCTTTGACACTGAGGTCTTTAAGAGATGGAAGACATGGCAGTCCAAGTTTAGGGCATTCG
ATGATTAGCAACTTAGAAAGACAAGGAAACATCTCCCCTCTTTCCACTTTCAATAACCCCTCTATGTTTGG
TAGGAATTCTAATACGAGTACCTCCAGAGATGGGAAAACCCTCACCTCCATACCATCCTCAGATTCATCAT
CATCCAAGTATTTCAGATTATCCATATCAGATAATTCCAGTTTTTTTTAGAGATGGTAGTTTTCCAAGTAA
CGGAAGCCGCACAATTTTGTTGCAATCCTCAAGTTTAAGAGAAATTAAATTACTAAGAAGGCTTATCCACA
TGGTAAAGATAATCCGGTGATAGCACTTTAT

NF10547-R-4
CATCATCAATATACATCCTTCTTAATTCCATTTTTCATTCATTTTCCTTCATCAAGCAATTAACAGTTTTC
TTTGGAGATCTTCTTCACATCTCTTTCTATTTGCCATGATAATCTCCTTGACGGGATGCTAGGATCACATA
TCCTCTGCTTGGTAATTGCTCCTTGTTGGCCTCCTAAATAAAGGGATAAACGAAATTCTAATTCCAAGGTT
GGTCAATAATCTGTTAAGAAAGGATTGTTGAGCAAAAAGCCGATCTTTATGGTTGTTTTCAAAAAAAAAAT
TTCTTTCAAAAAACATGTTAAAAAAATATAATTGATCTCGGAAGCCTCGATTAATTTCTCTATATTGTTGG
ACGCAAAAGTTTTTGTGTGTAGGTCCCATAAAAAAACAACCTTTCTTTCTTCTGTGACAGTTCCAAACAGA
GGAATCACACTTTTCAAGAAACTTTTCATCCTTCTTACTATCTTTTCACTTTCTTTCTTTTGTATAAGTTA
ACATGAACCTTTAATTAATTGTAGTAACTTATTCATAATTTTTCAAAAATATCCTATAAATTATCGTAAAA
TATTTTGTTTAAATCATGATAGAGAAAACATGTTGTTGACGGCATTGTAAGAAAATGGCGCCACTGAACTC
TCTTTCGAAAGAGTATCCACTCATCGACTCTAACTTCCAAACTTTCTGCGCTTCCCACGCCATTTATTCTG
GTCGTTCCTCTCTTCCTCATTTTCCCCTTTTTCATCTTTCAATTTCATCTAACACCATCATTATTCATTTT

NF10547-R-5
CATCATCAGTTCATTCTATTCTTCATAAGATGATGAGATACTTTGGTCCGCAAACTAGACTATCTTCTACT
TTAATGTTGAGATGTTGTTGAACATTTTACTAGTAGGTATTATTTAAAGAGTAGAGAACAACAAAATTTAA
TATTTTAATACTTTAAATTAATAGTATAATCGAAAGAGAAAAAAATTACACAAAAACTAGCTATCAAATCT
AGATTTCTCATTTGAAGAAGTAGTTGATGGATTTTTCTTTTCTAGTTCTATTCTTTTTTTAAATTAGTAAC
AAATGGAAGAAAATTTTCAGAAGTGATTATTTTGTTTAAAACAAACTATAATCAACAACATGTATGTTTTG
TATTATGGTGACAATAATTGATTTTGATTGAATTTTTTATGTAAAATTGATGGTGACAACTAGGGTATTCA
TGGAAGAATGATGGGTAATTTACCCCAACCAATACATATTAGCCACATAAGCACATTTTTAAGTGGTATCC
ATGAACTATCTTAACCCCACCAACAAATTGCTCATTTTCATTGGTTGGTGGGTAAATTACCCACCATTCTT
CAATGGTGATCTAGAAAAACACAAAATGGATTTCTATAAGAGGAACACTATATAGTACGGAAAATCTACAG
TACGGAGTGACACGATGCATGCGGATGGTGTTGTTATATTTTTAAAACAAATAAAGTTGAAATGTTTTACG
ATCTAAAATTAT

NF10547-R-6
CATCATCAGTATATGGTAAAAAAAAACTCTTTTTGGCACACGTAACTTTAACTTTTGTCCAAAAACTGTGA
AAAAATGCAGGCCATTGCTATATATAGCGAAGAAATATCCAACGATTTTGTCCCGAACTGAGAAAAGCTGT
TAAAAAGCACCCCACTAATAACCATACATGAGGGTGGTGTTATTAGTAAACTAATTACTTATTGTACATAG
GAACAGCATTTTCCGTGAAAAAAATTGAAAACACAATTTAGAGCCTAAAACTAAAGTTGATAGTATATTTA
GAAGGATTAAAAACATATTTAACTTATAAATAAATAAATAAAAGTATTGACCTGGCTCATCTAAATGCCAT
TAAATGCGTAAATATGCTTGCTCCATCTTCCTTACATTCCAAAATCTGCATCTAACTCAAATCACAAAGTA
ATTAGAAAATGAAGAATCACTCCTTAGAGTTGTTTCTTCTTCTAACTCTCTTCCTTCATGCAAATGCAGGA
GACTCCAATGTAAGTATTTTAATTACACTCTTCACTTCATTTAATTACACTAACCTCATTTAATTTCCATT
TGATCTTGTAACTCTAATGATCAGCATATTCATGTAGTAATAATCATTATCATTTGTTTGGATTAAACAAT
ACTCACATTATTACATCCAAAAAACCTCACTCCATTTTGATAGGAGCCAGATCCAATACGACCTTATTGAA

NF10547-R-7
CATCATCAAAAAGAACAAGGGTTATGATGAATAGCATGAACACCAACATCAACATCAACGTTTCTTCTCAT
TCTCATGATGTTATTGTTCCATCATCACTCATGTCTCCTCTTCAGAATCAATCACTTTTTAACCCTCATTC
AAATGGATTTCCTTTTTGGAGCAGCGATGAGATTATCAACAACAATGATCATCATCATCAATTGTCTCTTG
ATCTTTCTGGTACGACCCTTTTGGATAACAACAATGAGCAACAAACAAGAGAAGCTAGTAATGAGATGATG
ATGATGTTGTTAATGGTTGTTGTTTCTTAATTTTTTTCTGGATTTTTTTATAAATATTCATGAAGATGATG
AAAAAAGTGGAAGGAAGAAGATGAAGAAAGCAAAAAAAAAAAAAAAAAAAAAAGTCATTTAACAGCCAAAAC
AAACGGAGGGGGGTAATTGACTAACGTTTGTCAATTTCAGGGGTTTTTTTGAAGTTTCCTAAAAATCAGGG
GGATTTTTGACGAAAGCTACAATTTCAGGGAGGTTTTTGCTTATTTACTCCATAAATTTCTAAGCCTAGGA
CTGTTTAAAACGTGAAATGTTATTTCAACAATTATTATTGAAATAATTTACGGAGGAACAACTAAAGATAT

ATAAAAAAAATTAAAAATTGATATGAAAACTA

NF10547-R-8
CATCATCACATCCAATCATATTCATTCACTTAGTTATATTAGTTCCTAAAATATCTATGTGGTGAAATATG
ATTGGATACATGTATAAAAAAAATTACACTGACAGTGTATACCAATTAAAGTCTATTCTATTTCATGTGAT
GTAAACATTTATACTTTGTTGTAGACTTTAAAATTCCTTCAAATGGATTGTCTGCTGGAGCAATTGCTGGA
ATTGTGATCGGATCATTGGCATTTGTCATGCTGATACTCTTTGTCCTTTGGAAGATGGGTTACCTTTGTGG
AAAAGATCTAACAGACAAAGGTAAGATTGAAGTCCTAAATAATCATAACAGTTACTGCCCTTATGATTTGA
TAATTTTACAATAACGATCCAAAAATTGGATAATTTTAACGGTCAAATATGCTTTTCGTCCCTACAAAGGT
TGACTTGGTTTGTATTATATAATTACATTTGCTATTATATCTTTCCTTTTATGGTCACTTCTTGTTTCTGA
GTAAAATACCTATCTTATTGCAGAGCTCCTAGAATTAAAAACAGGCTATTACAGTTTGAGACAAATTAAAG
TTGCTACTAATAACTTTGATCCACAAAATAAGATAGGTGAAGGAGGATTTGGTCCAGTTTACAAGGTAAAT
ATGGATAAAGTAGTTGTAAATTTTACTTCA

NF10547-R-9
CATCATCAGATGTAGCTGAATATTTGATGAACTGCTTCTGCTATGTCATTTGCTGTGCCTAGCTTACAATC
TTCTTCAATCTGTATCACCCATATATACAGCTCATTATTGATTTATTTATCAAATAATAATAAATAGAACT
AATAAAAAACCAGAAAATTTGTCAAAAAAAAAAAAACTTAAAATGTGTTCAATGTAGCATGTATTCCAATC
TGAAAACAAGCTGATGATGTGGTCGTCTTAGGTAATTTATGGACCACCTCATTTGCTTGTGTCCCTACAAA
CTCAACACTAAAGTTGACATAATATTGTTCAAAAATAATTTTATAATTATGAATAATCATCCCAAAATCTA
TAATATCCTGATGTGCAGGTAAATAAATAATTCAAAAATTTAAATGATCCGACTAAATTATTTTAGTTAAA
TTTTTTTATTTATGTGTATGGCCGAATACGTGTTGTGACCAAATAAAACTTGCAACAATAATCATAGTTCC
TCTTTCAAATAGTGTGTTCTTTTTTTTTTTGGTCAATATCAAATAGTGTGTTCTAAAAACAACATTAGAGA
AATAAAAAATTATTATCAATTAAAGTAACATTAAAAACTAGTTAATATTAATTAGTTTATATGAAAATTTG
AACTCT

NF10547-R-10
CATCATCACGACGGACTATGGGCCAGCCCTGCTTCGTCGTTGCACGAACTGTGCATGTTTATTGGCGGAGA
AAGCATTGTCGGAGTCCATTCACTATTTTTCTAAGGAATGTTTATATTCAGAATTATAAGAAAAAAGTTAA
TTTCATGATAATTAAGAAAGTTAGTTTCTTCAAAAAAAAAAAAAAAAAGTTAGTTAAGTGCAATTAATTTT
TTAAATTTTGTGTAAAATATACTTTCAGTCGCTTTTAGGCCACACACCTTGCTATTAATTTACTGAACTAC
CTACCTTTGTTAATATACGATAACAAAGTTCACTAATATCCTTATGTATATCTAAGTTGGTAAAGATATTG
CTTTTTATAAGTATGAATCGAGATTTAAATTCTAAATTCGTCACTTATTCATCTTAAGATGAAATTTCAAC
CACTATGTTACTTTGAGAAAAATATAAAAGCACTCAACTATTTCTATGCTAAAAACATCATCTTTAAATAG
TTTTGATGCAGTTAACTTTTTACACCAATTAAATATTTTAAAATCACATTTGATTGGTGTTTTTTGACCGC
GTAAAACTGTTTAAGTGAATAGTAGTTATACTAGCATTTTCGTATTAAGCTTTCCCCACCTTATTTTTTCC
AT

NF10547-R-11
CATCATCAAACCGTTCTCATCATGCTCGTCCTAAGTCTAAAAGTGGTGAAGGAGTGTCTGCGTTTTTGTTA
ATGGTTGGCATAATGGTTGTCATTTATGTTGTATGTCGTTGTACAGAAAACAATGATACTCCAGTTACTGT
ACTCTAGCCTCAGGTTTGTTAACAAAAACATCTATTCTTGTCTGTGCAATGACAGTGTGTACTGTAATATC
TCTAAGACATTGTAAATGTTTTTGTGTCTGTTGTTTAAGGTTGGAATGTTGGATGAGATGGGATGCACATT
ACAAACGGATCTAGCTAGGATTGCATAAGCTGCTAATACATCCTCCAAGGAAGGTGTGCGCTGTTTATTGA
AAGGTGAGTGAGTTGCTTTTGATTCTAATATTTTTAGAGTTTAATTTATATACATCTTTAGTGTACAAAAT
TTTTACACATGCATCCAATAATGCTTTGCCAAATCAGTTGATGAGGTATGTCAAATGTGTTTTTAACTCAT
TAAATAATGTGACAACATGCCATTGAAACGATTCAAAAACTAAAATGACTTTGTTTGGGACTTTTTGAAAC
TTAAAACTACTTTGGGAAAAAACGTAAACTTAA

NF10547-R-12
CATCATCAGGTGCTTCAATGGTCATTAATTGGTGAATATTGTTTTTTCCTTCCTCTTCATGATGTTAGCTA
CAAAAATAGTACAGTGGTCAAAATAGAAACAAAATTGTATTATCCAGCTTGCAACGACGCTCGTACGGTAC
TATTAATTACTCCAAAAATCTGCATGAATATATTCTTAAATCAAAGATCACAATGTATCTCTTAAGATTAG
TGTATAACAATCAACTAGTTGAGGTTTGTTTAAACTTTAAAGTAGACGTTAACATTAACTCAAGCATATCA
AATGAACAATTAGTGATGGGAGTTAGTTTGAATAAACTTCTCTCTAAACACTTAAGAAAAATTTGTATACC
ATTTTCCTTATTACTTTGATTAAAAATAAAAAATAAAAACTAATGATAAAACAGTAATAGAATTGTGGATA
TACCGATCCCCACAATGCAGGTGCAAATAGTTCCTTCAAATTGATTTGTCGCTTAATATTTTTAGATGTTT

CATAATGTTTG

NF10547-R-13
CATCATCAATCTGAATCACCAACCTTCCTAACTTTATACGAACCATTCAAATTTGGATTTGAAGAGGTTTC
TTTCTACACCAAAAACAACAAATTCATCCCAGACAATAGCTGAAACTCATGAGGAAAAGTCTCATGATTGT
TTCCCTAAATTTTTCAAACAATTATTACATTTTTTTTTGAATAAAACAACCAACAATTATTACATATAATG
TCTTTAATTTATATACAGAAAAAATGATACAACAAGAAAATAACGTTAATATAATAAGAACAAATGATATG
AGAACAAACATATAATTTATGCTAAAATAAATAATCACCTTTTCTTAAATTCTACAGCTTGATGAATACAA
GGATTTACATAAATTCTGGTGA

NF10547-R-14
CATCATCAAGACTCTCTTGTGAATTGCAGTGGTAGCAATA
