# Supplementary material for: Rapid identification of causative insertions underlying Medicago truncatula Tnt1 mutants defective in symbiotic nitrogen fixation from a forward genetic screen by whole genome sequencing
Source: BMC Genomics. 2016 Feb 27;17:141. doi: 10.1186/s12864-016-2452-5 (PMC4769575; doi:10.1186/s12864-016-2452-5)
Supplement: Additional file 11: Figure S1. — Comparison between nodule phenotypes of NF11217 (dnf2-5), NF0217 (dnf2-2) and R108 (WT). (PDF 673 kb) [file 12864_2016_2452_MOESM11_ESM.pdf]

Additional File 11: Figure S1

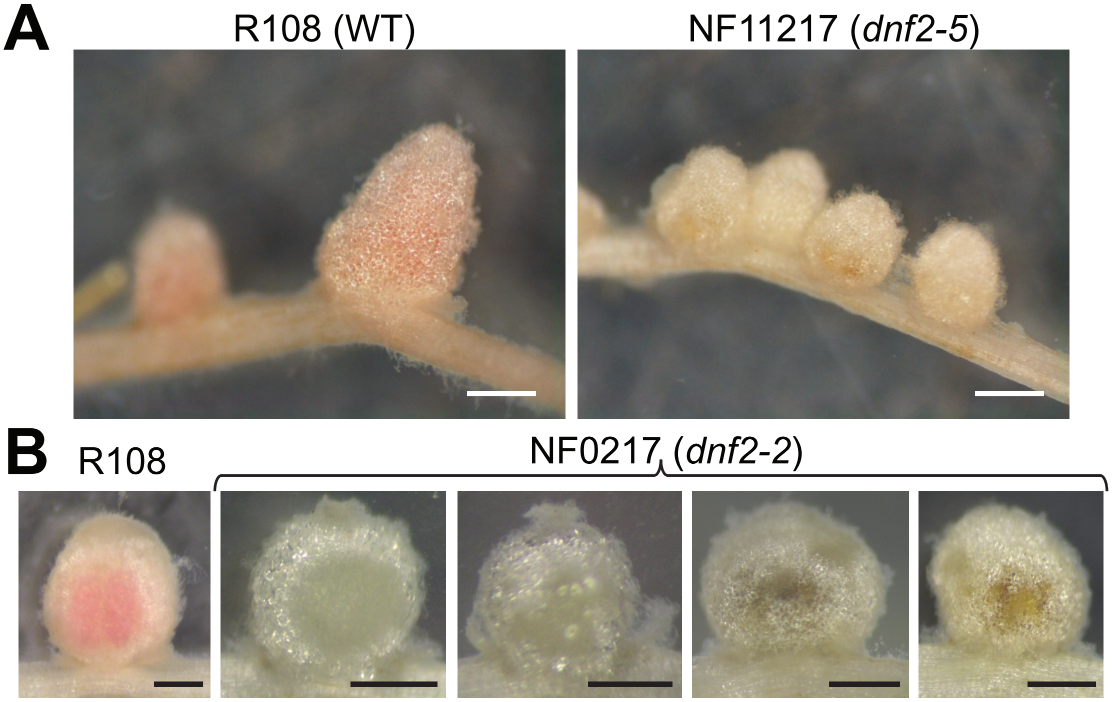

Additional File 11: Figure S1: Comparison between nodule phenotypes of NF11217 (*dnf2-5*), NF0217 (*dnf2-2*) and R108 (WT). NF11217, NF0217 and R108 were grown in aeroponic chambers and inoculated with *S. meliloti*. At 15 days post-inoculation, nodules were photographed. **(A)** NF11217 and R108 were grown side-by-side, inoculated and imaged. **(B)** R108 and NF0217 were studied in a separate experiment by Drs. C.I. Pislariu and R. Dickstein. Images in (B) are courtesy of Dr. C.I. Pislariu, current address: Department of Biology & Chemistry, Texas A&M International University, Laredo, TX 78041. Scale bar=0.5 mm.
