# Supplementary material for: Rapid identification of causative insertions underlying Medicago truncatula Tnt1 mutants defective in symbiotic nitrogen fixation from a forward genetic screen by whole genome sequencing
Source: BMC Genomics. 2016 Feb 27;17:141. doi: 10.1186/s12864-016-2452-5 (PMC4769575; doi:10.1186/s12864-016-2452-5)
Supplement: Additional file 12: Table S9. — Co-segregation analysis of NF11217 Nod + Fix- phenotype with Tnt1 insertion in PLC-like gene. (PDF 41 kb) [file 12864_2016_2452_MOESM12_ESM.pdf]

Additional File 12: Table S9. Co-segregation analysis of NF11217 Nod+Fix- phenotype with *Tnt1* insertion in *PLC-like* gene.
(Veerappan et al. 2015, submitted to BMC Genomics)

**Plant line**	**Phenotype**	***PLC***
1	Mutant	Defective
2	Mutant	Defective
3	Mutant	Defective
4	Mutant	Defective
5	Mutant	Defective
6	Mutant	Defective
7	Mutant	Defective
8	Mutant	Defective
9	Mutant	Defective
10	Mutant	Defective
11	Mutant	Defective
12	Mutant	Defective
13	Mutant	Defective
14	Mutant	Defective
15	Mutant	Defective
16	Mutant	Defective
17	Mutant	Defective
19	Mutant	Defective
20	Mutant	Defective
21	Mutant	Defective
22	Mutant	Defective
23	Mutant	Defective
24	Mutant	Defective
25	Mutant	Defective
26	Mutant	Defective
27	Mutant	Defective
28	Mutant	Defective
29	Mutant	Defective
30	Mutant	Defective
31	Mutant	Defective
32	Mutant	Defective
33	Mutant	Defective
34	Mutant	Defective
35	Mutant	Defective
36	Mutant	Defective
37	Mutant	Defective

38	Mutant	Defective
39	Mutant	Defective
40	Mutant	Defective
41	Mutant	Defective
42	Mutant	Defective
43	Mutant	Defective
44	Mutant	Defective
45	Mutant	Defective
46	Mutant	Defective
47	Mutant	Defective
48	Mutant	Defective
49	Mutant	Defective
50	Mutant	Defective
51	Mutant	Defective
52	Mutant	Defective
53	Mutant	Defective
54	Mutant	Defective
55	Mutant	Defective
56	Mutant	Defective
60	WT-L	Het
61	WT-L	WT
62	WT-L	Het
63	WT-L	Het
64	WT-L	Het
65	WT-L	Het
66	WT-L	Het
67	WT-L	Het
68	WT-L	Het
69	WT-L	Het
70	WT-L	WT
71	WT-L	WT
72	WT-L	Het
73	WT-L	Het
74	WT-L	Het
75	WT-L	Het
76	WT-L	Het
77	WT-L	Het
78	WT-L	WT
79	WT-L	Het
80	WT-L	Het
81	WT-L	Het

82	WT-L	WT
83	WT-L	Het
84	WT-L	Het
85	WT-L	Het

To test for the co-segregation of Nod+Fix- phenotype in NF11217 *Tnt1* insertion line, wild-type like (WT-L) or Nod+Fix- (mutant) plants from BC_1F_2 population obtained from NF11217 X R108 crosses were genotyped using either *PLC* (Medtr4g085800) gene specific primers Medtr4g085800-1F and Medtr4g085800-1R (WT locus) or *PLC* gene specific primer Medtr4g085800-1F with *Tnt1* specific primer *Tnt1-F1* (mutant locus). Genotyping primers are listed in Additional file 15: Table S11.
