# Supplementary material for: Rapid identification of causative insertions underlying Medicago truncatula Tnt1 mutants defective in symbiotic nitrogen fixation from a forward genetic screen by whole genome sequencing
Source: BMC Genomics. 2016 Feb 27;17:141. doi: 10.1186/s12864-016-2452-5 (PMC4769575; doi:10.1186/s12864-016-2452-5)
Supplement: Additional file 13: Table S10. — Co-segregation analysis of NF10547 Nod + Fix- phenotype with Tnt1 insertion in NIN gene. (PDF 42 kb) [file 12864_2016_2452_MOESM13_ESM.pdf]

Additional file 13: Table S10. Co-segregation analysis of NF10547 Nod+Fix- phenotype with *Tnt1* insertion in *NIN* gene.
(Veerappan et al. 2015, submitted to BMC Genomics)

Plant line	Phenotype	*NIN*
1	Mutant	Defective
2	Mutant	Defective
3	Mutant	Defective
4	Mutant	Defective
5	Mutant	Defective
6	Mutant	Defective
7	Mutant	Defective
8	Mutant	Defective
9	Mutant	Defective
10	Mutant	Defective
11	Mutant	Defective
12	Mutant	Defective
13	Mutant	Defective
14	Mutant	Defective
15	Mutant	Defective
16	Mutant	Defective
17	Mutant	Defective
19	Mutant	Defective
20	Mutant	Defective
21	Mutant	Defective
22	Mutant	Defective
23	Mutant	Defective
24	Mutant	Defective
25	Mutant	Defective
26	Mutant	Defective
27	Mutant	Defective
28	Mutant	Defective
29	Mutant	Defective
30	Mutant	Defective
31	Mutant	Defective
32	Mutant	Defective
33	Mutant	Defective
34	Mutant	Defective
35	Mutant	Defective
36	Mutant	Defective
37	Mutant	Defective

38	Mutant	Defective
39	Mutant	Defective
40	Mutant	Defective
41	WT-L	Het
42	WT-L	Het
43	WT-L	Het
44	WT-L	WT
45	WT-L	Het
46	WT-L	WT
47	WT-L	Het
48	WT-L	Het
49	WT-L	WT
50	WT-L	WT
51	WT-L	Het
52	WT-L	Het
53	WT-L	Het
54	WT-L	Het
55	WT-L	Het
56	WT-L	Het
57	WT-L	WT
58	WT-L	Het
59	WT-L	WT
60	WT-L	Het
61	WT-L	WT
62	WT-L	WT
63	WT-L	WT
64	WT-L	Het
65	WT-L	WT
66	WT-L	Het
67	WT-L	WT
68	WT-L	WT
69	WT-L	Het
70	WT-L	WT
71	WT-L	Het
72	WT-L	Het
73	WT-L	Het
74	WT-L	Het
75	WT-L	Het
76	WT-L	Het
77	WT-L	Het
78	WT-L	Het

79	WT-L	WT
80	WT-L	WT

Co-segregation analysis was performed using Nod+Fix- mutant plants (purple leaves and white nodules) and wild-type like (WT-L) plants (green leaves and pink nodules) obtained from R108 X NF10547 BC_1F_2 population. *NIN* (Medtr5g099060) gene specific primers Medtr5g099060-1F and Medtr5g099060-1R were used to genotype the wild-type locus whereas Medtr5g099060-1F and *Tnt1*-R1 primers were used to genotype the *Tnt1* insertion in *NIN*. Genotyping primers can be found in Additional file 15: Table S11.
