# Supplementary material for: Rapid identification of causative insertions underlying Medicago truncatula Tnt1 mutants defective in symbiotic nitrogen fixation from a forward genetic screen by whole genome sequencing
Source: BMC Genomics. 2016 Feb 27;17:141. doi: 10.1186/s12864-016-2452-5 (PMC4769575; doi:10.1186/s12864-016-2452-5)
Supplement: Additional file 15: Table S11. — Sequences of primers used in this study. (PDF 7 kb) [file 12864_2016_2452_MOESM15_ESM.pdf]

Additional File 15: Table S11. Sequences of primers used in this study
(Veerappan et al. 2015, submitted to BMC Genomics)

Primer Name	Primer sequences (5’-3’)	Comments
Medtr5g099060-1F<br>Medtr5g099060-1R	TGCTAATGGTGGTGATGGTAAT<br>GGTTAAATCGCCTTGCAATCTC	NF10547 co-segregation analysis genomic primers
Medtr4g085800-1F<br>Medtr4g085800-1R	GCACAATATTTACCGCCTTTGT<br>ACAATGACCTCTTCCCATTCC	NF11217 co-segregation analysis genomic primers
Tnt1-F1<br>*Tnt1*-R1	TCCTTGTTGGATTGGTAGCCAACTTTGTTG<br>TGTAGCACCGAGATACGGTAATTAACAAGA	*Tnt1* primers for used for co-segregation analysis
AD1<br>AD2<br>AD3<br>AD5<br>AD6	NTCGA(G/C)T(A/T)T(G/C)G(A/T)GTT<br>NGTCGA(G/C)(A/T)GANA(A/T)GAA<br>(A/T)GTGNAG(A/T)ANCANAGA<br>(G/C)(G/C)TGG(G/C)STANAT(A/T)AT(A/T)CT<br>CG(G/C)AT(G/C)TC(G/C)AANAA(A/T)AT	Arbitrary degenerate (AD) primers used in TAIL-PCR reactions
Tnt1-F<br>*Tnt1*-R	ACAGTGCTACCTCCTCTGGATG<br>CAGTGAACGAGCAGAACCTGTG	*Tnt1* primers used in TAIL-PCR reactions
