# Supplementary material for: Rapid identification of causative insertions underlying Medicago truncatula Tnt1 mutants defective in symbiotic nitrogen fixation from a forward genetic screen by whole genome sequencing
Source: BMC Genomics. 2016 Feb 27;17:141. doi: 10.1186/s12864-016-2452-5 (PMC4769575; doi:10.1186/s12864-016-2452-5)
Supplement: Additional file 14: Figure S2. — Comparison of Mtnin-16′s encoded sequence with that of Lotus japonicus NIN and M. truncatula NIN. (PDF 797 kb) [file 12864_2016_2452_MOESM14_ESM.pdf]

```
                                                         Block I                                                  Block II
LjNIN      MEYGSLLVQQQQQQDCNSAYGSLSNLSSDCGSVTTAEADHHIIEELLVQGCWVEVSGVGVR-----------------------EG-----------------------ELQLQQDESSF   74
MtNIN      MEYGGGLVADGG------VFGPMV---G-----GGGGDQADIIEELLGEGCWIEASENSLMAMQQTTPQSQYMSNNNNIPMGMGEGDHFNHHHHHHPHPHHQMECTAPAANHDDQQESGF  106
Mtnin-16   MEYGGGLVADGG------VFGPMV---G-----GGGGDQADIIEELLGEGCWIEASENSLMAMQQTTPQSQYMSNNNNIPMGMGEGDHFNHHHHHHPHPHHQMECTAPAANHDDQQESGF  106
           ****. ** :         .:* :     .        .  : .****** :***:*.*   .:                        **                           : :*:**.*

LjNIN      VVGKRWWIGPAAAVAGSCNSSVKERLVIAVGYLKDYTRNS--NVLIQIWVPLRRGILHDHDYHTNYLLSNNPPPQPEAAADHESVSLGFPMPAAPNSNLYSNVHVRFFRSHEYPRVQAQ-  191
MtNIN      VVGKRWWIGPRANPG--PTTSVKERLVVAVGYLKEYTKNSSNNVLIQIWVPMRRRSALIHTQN-HYLQQES---------------SSAPV----SVNPNMNVHVRFFRSHDYPRHQQQQ  204
Mtnin-16   VVGKRWWIGPRANPG--PTTSVKERLVVAVGYLKEYTKNSSNNVLIQIWVPMRRRSALIHTQN-HYLQQES---------------SSAPV----SVNPNMNVHVRFFRSHDYPRHQQQQ  204
           ********** *   .  .:*******:******:**:**  *********:**      *  . .** .:.             . *:     . *   **********:*** * *

                         Block II                                              Block III
LjNIN      QYGS-LALPVFERGTGTCLGVLEIVITNQTTINYNVSN----ALDQAVDFRSSQ-SFIPPAIKVYDELYQAAVNEIIEVMTSVCKTHNLPLALTWAPCIQQGK---------CGCGVSSE  296
MtNIN      QYGSLLALPVFERGSGTCLGVIEFVISNQTLINYRPQLDHLSNALEAVDFRSSHNMNIPQAVKVFEELYEAAVNEIMEVLASVCKTHNLPLALTWAPCLQQQQGGGKGSSGASGCGVS--  322
Mtnin-16   QYGSLLALPVFERGSGTCLGVIEFVISNQTLINYRPQLDHLSNALEAVDFRSSHNMNIPQAVKVFEELYEAAVNEIMEVLASVCKTHNLPLALTWAPCLQQQQGGGKGSSGASGCGVS--  322
           **** *********:******:*:**:*** ***. .           :*******:   ** *:**::***:******:**::*****************:** :           .*****

                                                                  Block IV
LjNIN      NYMWCVSTVDSACFVGDLDILGFQEACSEYHLFRGQGIVGTAFTTSKPCFAIDITAFSKAEYPLAHHANMFGLHAAVAIPLRSVYTGSAADFVLEFFLPKDCHDSEEQKQLLNSLSMVVQ  416
MtNIN      TMSCCISTVDSACYVGDMDVLGFQEACSEYHLFNGQGIVGTAFTTTKPCFAIDITAFSKSEYPLAHHANMFGLHAAVAIPLRSVYTGSAADFVLEFFLPKDCRDTEQQKQMLNSLSLVVQ  442
Mtnin-16   TMSCCISTVDSACYVGDMDVLGFQEACSEYHLFNGQGIVGTAFTTTKPCFAIDITAFSKSEYPLAHHANMFGLHAAVAIPLRSVYTGSAADFVLEFFLPKDCRDTEQQKQMLNSLSLVVQ  442
           .   *:*******:***:*:*************.***********:*************:***************************************:*:*:***:*****:***

            Block IV                                                                  Block L
LjNIN      QACRSLHVVLVEDEYTL-PMPSHTSKEELEEEEITITNNHE--Q--------KLFVSPSSHESECSKESSWIAHMMEAQQKGKGVSVSLEYLEEPKEEFKVTT-NWDSSTDHDQQAQVFS  524
MtNIN      QACRSLHLHVVMDDNNNNNMNDNNSSADHDHDQFTFPTTNSYMPSSASEPLSQVDAVSGCSTKDTSSSCSWIAHMMEAQNKGKGVSVSLEYLQEPKEEFKVTTCNWDREREDNV-----F  557
Mtnin-16   QACRSLHLHVVMDDNNNNNMNDNNSSADHDHDQFTFPTTNSYMPSSASEPLSQVDAVSGCSTKDTSSSCSWIAHMMEAQNKGKGVSVSLEYLQEPKEEFKVTTCNWDREREDNV-----F  557
           *******: :* *: .    * ...*. : :.:::*: ....                :: .  ..  .: *...**********:************:********** *** . :.:

                                                                                           Block V = RWP-RKP Domain
LjNIN      SDFGQMSS--------GFK--ASTVEGGDQESSYTFGSRRSSSGGRKSGEKRRTKAEKTISLQVLRQYFAGSLKDAAKSIGVCPTTLKRICRQHGITRWPSRKIKKVGHSLKKLQLVIDS  634
MtNIN      SEFGQVLQQQQHDQSSNSRASVVSVEAGEESPGACGRRSSSSSSGRKSGDKRRTKAEKTISLPVLRQYFAGSLKDAAKSIGVCPTTLKRICRQHGITRWPSRKIKKVGHSLKKLQLVIDS  677
Mtnin-16   SEFGQVLQQQQHDQSSNSRASVVSVEAGEESPGACGRRSSSSSSGRKSGDKRRTKAEKTISLPVLRQYFAGSLKDAAKSIGVCPTTLKRICRQHGITRWPSRKIKKVGHSLKKLQLVIDS  677
           *:***: .              :  . :**.*::. .          ***.*****:************ ***********************************************

         Block V = RWP-RKP Domain
LjNIN      VQGAEGAIQIGSFYASFPELSSSDFSASCRSDSSKKMHNYPDQNNTLYGHGDHGGVVTSLKSPPSACSQTFAGNQPC----TIINNGDVLMTESPPVPEALLS-RRDHCEEAELLNNASI  749
MtNIN      VQGAEGAIQIGSFYASFPELSNATANGGDG---------NDNSNNSFYNNNHGDGIVTSLKSPPSACSQTHAGNKLPMTTTTAINHHHVVMTENPTGAPLGVDHAFMHASNINIQDYHQL  788
Mtnin-16   VQGAEGAIQIGSFYASFPELSNATANGGDG---------NDNSNNSFYNNNHGDGIVTSLKSPPSACSQTHAGNKLPMTTTTAINHHHVVMTENPTGAPLGVDHAFMHASNINIQDYLMM  788
           ********************.:   ...               :.**::*  .  *:**************.***:       * **. .*:***.*        :.     *..: :: :  .:

                                                                                     Block VI = PB1 Domain
LjNIN      QEDT--KRF---SRPKSQTLPPL-------SDSSGWNSLETGAFRVKATFADEKIRFSLQPIWGFSDLQLEIARRFNLNDVTNILLKYLDDDGEWVVLACDGDLEECKDIHRSSQSRTIR  857
MtNIN      QEDLDTKQLLLHFNNNNQILPPRPTVAWNNNNSSSSTLLERGAFRVKATFADEKIRFSLQAMWGFRDLQLEIARRFNLTDMNNLVLKYLDDEGEWVVLSCDADLEECKDLHTSSHTRTIR  908
Mtnin-16   SISLKKY-----------------------------------------------------------------------------------------------------------------  795
           ***   *::    . :.* ***        .:**. . ** ****************** :*** ************.*:.*::******:******:**.*******:* **::****

         Block VI = PB1 Domain
LjNIN      LSLFQASPLNLANTFRNSSPS----                                                                                                878
MtNIN      LSLFQASPLNLPNTFRNSSSSSPSS                                                                                                933
Mtnin-16   -------------------------                                                                                                795
           *********** ******* *
```

Additional File 14: Figure S2: Comparison of *Mtnin-16*'s encoded sequence with that of *Lotus japonicus NIN* and *M. truncatula NIN*. The LjNIN sequence is Genbank accession CAB61243, the MtNIN sequence is Genbank accession ACN58567 and the Mtnin-16 sequence is from this work. The seven blocks of sequence shaded in gray correspond to the six blocks of sequence conserved in NIN and NIN-like proteins and block L that is conserved in NINs, NLP1 and NLP2 (Schauser et al. J Mol Evol (2005) 60:229–237). The ten amino acid residues highlighted in yellow in Mtnin-16 correspond to those encoded by *Tnt1* in the Mtnin-16 hybrid protein. Mtnin-16 contains the first six conserved blocks of sequence found in NIN proteins, but lacks the seventh block corresponding to the PB1 domain that is important for hetero-dimerization.
